# Identification and characterization of a new potent inhibitor targeting CtBP1/BARS in melanoma cells

**DOI:** 10.1186/s13046-024-03044-5

**Published:** 2024-05-06

**Authors:** Angela Filograna, Stefano De Tito, Matteo Lo Monte, Rosario Oliva, Francesca Bruzzese, Maria Serena Roca, Antonella Zannetti, Adelaide Greco, Daniela Spano, Inmaculada Ayala, Assunta Liberti, Luigi Petraccone, Nina Dathan, Giuliana Catara, Laura Schembri, Antonino Colanzi, Alfredo Budillon, Andrea Rosario Beccari, Pompea Del Vecchio, Alberto Luini, Daniela Corda, Carmen Valente

**Affiliations:** 1 Institute of Experimental Endocrinology and Oncology “G. Salvatore“(IEOS), National Research Council (CNR), 80131 Naples, Italy; 2 Molecular Cell Biology of Autophagy, The Francis Crick Institute, London, UK. The Study Has Been Previously Performed at IEOS-CNR, Naples, Italy; 3 Department of Chemical Sciences, University of Naples Federico II, 80126 Naples, Italy; 4 Animal Facility Unit, Istituto Nazionale Tumori-IRCCS-Fondazione G. Pascale, 80131 Naples, Italy; 5 Experimental Pharmacology Unit, Istituto Nazionale Tumori-IRCCS-Fondazione G. Pascale, Naples, 80131 Italy; 6 Institute of Biostructures and Bioimaging (IBB), National Research Council (CNR), Naples, 80145 Italy; 7 Interdepartmental Service Center of Veterinary Radiology, University of Naples Federico II, 80137 Naples, Italy; 8 National Research Council (CNR), Piazzale Aldo Moro, 700185 Rome, Italy; 9 Biology and Evolution of Marine Organisms (BEOM), Stazione Zoologica Anton Dohrn, Naples, Italy; 10 Institute of Biochemistry and Cell Biology, National Research Council (CNR), 80131 Naples, Italy; 11 Department of Pharmacy, University of Naples Federico II, 80131 Naples, Italy; 12 Scientific Directorate, Istituto Nazionale Tumori-IRCCS-Fondazione G. Pascale, 80131 Naples, Italy; 13 EXSCALATE, Dompé Farmaceutici S.P.A, Naples, Italy; 14 Present address: Dompé Farmaceutici S.P.A, L’Aquila, Italy

**Keywords:** C-terminal Binding Protein (CtBP), Rossmann fold, Benzenesulfonamide, CtBP inhibitor, Melanoma

## Abstract

**Background:**

The C-terminal-binding protein 1/brefeldin A ADP-ribosylation substrate (CtBP1/BARS) acts both as an oncogenic transcriptional co-repressor and as a fission inducing protein required for membrane trafficking and Golgi complex partitioning during mitosis, hence for mitotic entry. CtBP1/BARS overexpression, in multiple cancers, has pro-tumorigenic functions regulating gene networks associated with “cancer hallmarks” and malignant behavior including: increased cell survival, proliferation, migration/invasion, epithelial-mesenchymal transition (EMT).

Structurally, CtBP1/BARS belongs to the hydroxyacid-dehydrogenase family and possesses a NAD(H)-binding Rossmann fold, which, depending on ligands bound, controls the oligomerization of CtBP1/BARS and, in turn, its cellular functions.

Here, we proposed to target the CtBP1/BARS Rossmann fold with small molecules as selective inhibitors of mitotic entry and pro-tumoral transcriptional activities.

**Methods:**

Structured-based screening of drug databases at different development stages was applied to discover novel ligands targeting the Rossmann fold. Among these identified ligands, *N-(3,4-dichlorophenyl)-4-{[(4-nitrophenyl)carbamoyl]amino}benzenesulfonamide,* called Comp.11, was selected for further analysis. Fluorescence spectroscopy, isothermal calorimetry, computational modelling and site-directed mutagenesis were employed to define the binding of Comp.11 to the Rossmann fold. Effects of Comp.11 on the oligomerization state, protein partners binding and pro-tumoral activities were evaluated by size-exclusion chromatography, pull-down, membrane transport and mitotic entry assays, Flow cytometry, quantitative real-time PCR, motility/invasion, and colony assays in A375MM and B16F10 melanoma cell lines. Effects of Comp.11 on tumor growth in vivo were analyzed in mouse tumor model.

**Results:**

We identify Comp.11 as a new, potent and selective inhibitor of CtBP1/BARS (but not CtBP2). Comp.11 directly binds to the CtBP1/BARS Rossmann fold affecting the oligomerization state of the protein (unlike other known CtBPs inhibitors), which, in turn, hinders interactions with relevant partners, resulting in the inhibition of both CtBP1/BARS cellular functions: *i)* membrane fission, with block of mitotic entry and cellular secretion; and *ii)* transcriptional pro-tumoral effects with significantly hampered proliferation, EMT, migration/invasion, and colony-forming capabilities. The combination of these effects impairs melanoma tumor growth in mouse models.

**Conclusions:**

This study identifies a potent and selective inhibitor of CtBP1/BARS active in cellular and melanoma animal models revealing new opportunities to study the role of CtBP1/BARS in tumor biology and to develop novel melanoma treatments.

**Supplementary Information:**

The online version contains supplementary material available at 10.1186/s13046-024-03044-5.

## Background

Melanoma, a tumor that develops from an uncontrolled proliferation of melanocytes, is the most malignant form of skin cancer [1–4] and its worldwide incidence and mortality has been risen rapidly [5–7]. Melanoma contributes to more than 80% of skin cancer fatalities and, according to the latest SEER data, melanoma is the fifth most common cancer diagnosis in the US (excluding nonmelanoma skin cancers), with 97,610 estimated new cases in 2023 (National Cancer Institute Melanoma of the Skin-Cancer Stat Facts. https://seer.cancer.gov/statfacts/html/melan.html). Currently, it is the third most common tumor in adolescents and young adults [8]. When melanoma is diagnosed early, surgical resection of the tumor is associated with favorable survival outcomes, although, unfortunately, it is an aggressive tumor that tends to metastasize [6, 9, 10]. Once metastasized, resection is no longer sufficient, the malignancy is more difficult to treat, and long-term prognosis is poor [6, 9, 11, 12]. Recently, immunotherapy with inhibitors of immune checkpoints has proven to be particularly effective in the treatment of melanoma. Ipilimumab (marketed as Yervoy), an anti-CTLA-4 monoclonal antibody, was first approved by the FDA in 2011 for unresectable stage III and stage IV melanoma and has since gained approval for various other cancer types when used in combination therapies. Additionally, anti-PD-1 monoclonal antibodies, namely pembrolizumab and nivolumab (sold as Keytruda and Opdivo, respectively), have been approved for stage III and IV melanoma either as single agents or, in the case of nivolumab, also in association with ipilimumab. Furthermore, in 2020, a combination therapy consisting of the anti-PDL-1 antibody atezolizumab (branded as Tecentriq) along with the BRAF inhibitor vemurafenib (marketed as Zelboraf) and the MEK inhibitor cobimetinib (sold under the name Cotellic) received approval for the treatment of BRAF V600 mutation-positive unresectable or metastatic melanoma [7, 9, 13–17]. Despite the development of combinatorial approaches, their treatment efficiency faces several challenges due to the patient and tumor heterogeneity, and different resistance mechanisms. To overcome these resistance mechanisms and achieve better clinical benefits, it is necessary to explore the early steps of the biological determinants involved in melanoma development and progression.

Changes in transcriptional regulation represent an early event in tumorigenesis either controlled by mutations or modifications in the cell itself or by the tumor microenvironment. Numerous genetic events have been linked to melanoma’s pathogenesis [18]. To date the *CDKN2A* gene, which encodes for p16INK4a and p14ARF tumor suppressor proteins [5, 6, 19], is one of the major melanoma susceptibility genes identified [20]. The *CDKN2A* gene sequences is mutated, deleted, or methylated in 40–70% of sporadic melanoma and in the germline of 40% of families with cutaneous melanoma predisposition [19, 21, 22]. The p16^INK4a^ protein controls the melanoma cell-cycle progression [23–28], and its transcriptional induction is under the control of the C-terminal binding protein 1 (CtBP1) [29].

CtBP is overexpressed in many human cancers including melanomas where its expression level is associated with aggressive tumor features, worse clinical outcomes and poor survival [30–32]. In mammals, the CtBP family comprises five splicing variants of the two genes CtBP1 and CtBP2. The *CtBP1* gene codes for two splicing variants, CtBP1-L (long) and CtBP1-S/BARS (short). The *CtBP2* gene codes for three splicing variants, CtBP2-L, CtBP2-S, and RIBEYE [33–35]. Here, we will refer to the CtBP1-L and S variants as CtBP1/BARS and to CtBP2-L and S as CtBP2. Both CtBPs are recognized players in the initiation and progression of cancer, where they exert overlapping but not identical functions.

CtBP1/BARS is a dual-function protein localized to both the cytosol and the nucleus where it is involved in the fission of intracellular membranes and in transcriptional repression, respectively. CtBP2 localizes exclusively to the nucleus and presumably operates only in transcription [34–36].

As transcriptional co-repressors, the CtBPs act mainly by interacting with transcription factors and recruiting chromatin-remodeling proteins, to assemble in a transcription repression complex [34, 37–43]. The transcriptional activity of CtBP1/BARS promotes multiple pro-oncogenic activities and neoplastic phenotypes such as: *i*) epithelial to mesenchymal transition (EMT) (by repressing several epithelial genes *e.g., E-cadherin*, *keratin-8*; [44–46]); *ii*) cell migration/invasion [47–49]; *iii*) abnormal cell survival through repression of apoptotic genes (*e.g., Bik*, *Puma*, *Noxa*, *PARP*, *p21*, *ARF*; [40, 44]) and of tumor suppressor genes (*PTEN*, *p16*^*INK4a*^ and *p15*^*INK4b*^; [19, 29, 49–51]). Moreover, CtBP1/BARS transcriptional activity promotes disordered cellular metabolism [52, 53] and cancer stem cell phenotype [54, 55]. Reduced transcription of these genes is associated with aggressive tumor development [30].

CtBP1/BARS is highly expressed in metastatic melanoma cell lines, but rarely detected in normal melanocytes. This higher CtBP1/BARS protein level commits cell proliferation and genome instability endorsing melanoma initiation and progression [29] by CtBP1/BARS-mediated transcriptional repression of *CDKN2A* gene and *Brca1* gene [29]. CtBP1/BARS was initially identified as protein that interacts with adenovirus E1a protein [56, 57]. The E1a–CtBP1/BARS interaction is required for expression of epithelial gene involved in intercellular adhesion, *e.g., desmoglein 2*, *plakoglobin* and *E-cadherin*, as shown in melanoma cells [58].

As a membrane fission controller, CtBP1/BARS acts at different intracellular trafficking steps as well as in the fragmentation and partitioning of the Golgi complex during mitosis [35, 36, 59]. This latter effect is relevant in tumor progression, since Golgi fragmentation is necessary for mitotic entry [59–61], and inhibition of CtBP1/BARS causes a potent cell cycle arrest in the G2 phase [59, 62, 63] followed by apoptosis (reminiscent of the effects of anti-cancer drugs).

Structurally, CtBP1/BARS possesses a NAD(H) binding domain (nucleotide-binding domain, NBD) hosting a Rossmann fold, a tertiary fold found in proteins that bind nucleotides. The Rossmann fold and its ligands [NAD(H) or acyl-CoA, or similar molecules] can regulate the conformation as well as the cellular functions of the protein: NAD(H) binding promotes the nuclear “transcriptional-active dimeric conformation” while acyl-CoA binding promotes the cytoplasmic “fission-prone monomeric conformation” [35, 39, 63–69].

Furthermore, CtBP1/BARS acts as sensor of the metabolic status of cells based on its high NADH-binding affinity [54, 70]. Targeting the Rossmann fold of CtBP1/BARS with small molecules has been proposed as a potential anti-cancer therapy, aiming to disrupt its interaction with transcription partners and protein complexes involved in mitotic entry and transcriptional activities.

The first-time proof of principle study that small molecules could be used and developed as new pharmacological therapy for human cancers specifically controlled by CtBP activities has been provided by the anti-tumorigenic effects of MTOB (4-methylthio-2-oxobutanoic acid) [51, 54]. To date, three classes of small molecules that act as CtBP1/BARS inhibitors are recognized: *i)* MTOB [51, 71] and HIPP derivatives [71, 72] inhibit dehydrogenase activity; *ii)* cyclic peptide CP61 inhibits homo/hetero-dimerization of CtBP1 and CtBP2 [73]; *iii)* NSC95397 inhibits CtBP1 interaction with partners such as E1A [74]. High concentrations (10 mM) of MTOB, an intermediate in the methionine salvage pathway [75], are required to antogonize the CtBP-regulated activities in colon and breast cancer, while NSC95397 has been shown to be a weaker CtBP inhibitor than MTOB and also not being selective for CtBP1 activities [76–79].

Here, we report the identification of N-(3,4-dichlorophenyl)-4-{[(4 nitrophenyl)carbamoyl] amino} benzenesulfonamide, called Compound 11 (Comp.11), as an active small molecule able to inhibit the CtBP1/BARS protein-partners interaction, which affects the two cellular functions of CtBP1/BARS: transcriptional repression and membrane fission. Two cancer cell lines, human A375MM and murine B16F10 melanoma cell lines were used to test the activity of this molecule. Effects of Comp.11 on CtBP1 transcriptional activity results in reduced mesenchymal melanoma gene expression signatures, cancer cell proliferation, migration and invasion, and cell colony formation while effects of Comp.11 on CtBP1/BARS membrane fission activity results in mitotic Golgi-checkpoint block and exocytosis impairment. Altogether, this Comp.11-mediated control of CtBP1/BARS cellular activities impairs melanoma tumor growth in mouse models.

Conversely, MTOB, HIPP and PPγ are not able to antagonize the CtBP1/BARS-regulated activities in melanoma.

Therefore, Comp.11 may be a promising candidate for the development of new anticancer agents for the treatment of melanoma.

This paper is dedicated to the memory of Stefania Spanò, who suddenly and unexpectedly passed away on September 3rd, 2019.

## Methods

### Antibodies and reagents

The mouse monoclonal anti-GM130 (610823, 1:400) was from BD Biosciences (San Jose, CA, USA). The sheep polyclonal anti-TGN46 (AHP500GT, 1:400) was from Bio-Rad (Hercules, CA, USA). The rabbit polyclonal anti-CtBP1/BARS (1:20 for IF use) was raised against His–BARS by Covalab (Auvergne-Rhone-Alpes, France). The mouse monoclonal anti-CtBP1/BARS (BC3 clone, 1:200 for WB use) as reported in [64]. The rabbit polyclonal antibodies: anti-E-cadherin (ab15148, 1:1000), NRAS (ab167136, 1:500), LPAATδ (ab188002), anti-β actin (ab8227, 1:5000) and the rabbit monoclonal antibodies: anti-CtBP2 (ab128871, 1:1000), STAT3 (ab68153, 1:1000), MMP2 (ab92536, 1:500) were from Abcam (Cambridge, UK). The rabbit monoclonal anti-vimentin (#5741, 1:1000), anti-Snail (#3879, 1:1000), anti-NFkB (#8242, 1:1000), anti–ZO-1 (#13663, 1:1000) and anti-Akt (#4691, 1:1000) were from Cell Signaling Technology (Beverly, MA, USA). The mouse monoclonal anti-GAPDH (MCA4740, 1:50000) was from AbD Serotech. The mouse monoclonal antibodies: M2-anti-Flag (F1804, 1:5000) and the P5D4 Cy3-conjugated anti-VSVG (SAB42000695, 1: 400), anti-GST (8–326) (MA4-004, 1:1000) and the HRP-conjugated secondary antibodies were from Merck (Darmstadt, Germany). The mouse monoclonal anti-N-cadherin (33–3900, 1:1000), anti-penta-His (P21315, 1:5000) and Alexa 488, 568 and 647 conjugated secondary antibodies were from Thermo Fisher Scientific (Waltham, MA, USA). N-(3,4-dichlorophenyl)-4-{[(4 nitrophenyl)carbamoyl] amino} benzenesulfonamide (Comp.11) has been synthesized by Merck (Darmstadt, Germany). MTOB (HY-135046) and PPγ (HY-W012530) were from MedChemExpress while HIPP (H955540) was from Toronto Research Chemicals. NAD^+^ (N0632), arachidonoyl coenzyme A lithium salt (A5837), DMSO (D2438), thymidine (T1895), bovine serum albumin (BSA, A4503), propidium iodide (P4864) and Matrigel Basement Membrane Matrix (CLS354234) were from Merck (Darmstadt, Germany). Hoechst nuclear dye (62249), MTT reagent (L11939) and RNAse A (12091021) were purchased from Thermo Fisher Scientific (Waltham, MA, USA). Ni–NTA agarose resin (30230). QuantiTect Reverse Transcription kit and RNeasy Mini Kit were from Qiagen (Hilden, Germany). Glutathione Sepharose 4B GST-tagged protein purification resin (17075605) and Amersham ECL Western Blotting Detection Reagent were from GE Healthcare (Chicago, IL, USA). Protease inhibitor cocktail tablets (11836170001) and SYBR Green PCR Master Mix (04887352001) were from Roche (Basel, Switzerland). Total protein concentration was measured by the Bradford method (5000006, Bio-Rad, Hercules, CA, USA). Nitrocellulose membranes (HATF00010) were from Millipore (Burlington, MA, USA). The Oleoyl Coenzyme A, [oleoyl-1-^14^C] (NEC799005UC) and the L-α-DiPalmitoyl-Phosphatidylcholine, [DiPalmitoyl-1-14C] (NEC682010UC) were from PerkinElmer (Waltham, MA, USA). C18:1 LPA (857130) and Arachidoyl Coenzyme A (ammonium salt, 870720) were from Avanti Polar Lipids (Alabaster, AL, USA).

### Protein expression and purification

Recombinant purified His-CtBP1/BARS^wt^, GST and GST-CtBP1/BARS proteins were produced as described previously [80]. The same procedures were used for His-CtBP1/BARS^H304L^, His-CtBP1/BARS^G172E^, His-CtBP1/BARS^H66A^, His-CtBP1/BARS^R255A^, His-CtBP1/BARS^C226A^, His-CtBP2, GST–14–3-3γ and GST-E1A.

### Isothermal titration calorimetry

The binding of Comp.11 to CtBP1/BARS^wt^ and its mutants (CtBP1/BARS^H304L^, CtBP1/BARS^R86A^, CtBP1/BARS^G172E^, CtBP1/BARS^H66A^, CtBP1/BARS^R255A^, CtBP1/BARS^C226A^) were performed using a Nano-ITC III calorimeter (TA Instruments, New Castle, DE, USA) at 25 °C. The protein solutions (typically 20 µM) were added to the sample cell (1 ml), and the ligand solution (typically 100 µM) into the syringe compartment (250 µl). After temperature equilibration, the protein solution was titrated by adding 10 μl aliquots of the ligand solution at 400 s intervals between the individual injections. The rotating micro-syringe ensured the homogenization during injection of the whole mixed solution at a speed of 300 rpm. The heat of dilution from the blank titration of the ligand into the buffer was measured, and the dilution heats were subtracted from the raw data. Raw data are integrated, corrected for nonspecific heats, normalized for concentration, and analyzed assuming a single binding site by using the Nano-Analyze software supplied with the instrument and plotted using the Origin software package. Thermodynamic parameters, including the binding constant (K_b_), the enthalpy change (ΔH) and stoichiometry (n), were estimated by iterative curve fitting of the binding isotherms. The standard Gibbs energy and entropy changes were obtained by using the equations: ΔG = − RT ln K_b_ (R = 8.314 J mol^−1^ K^−1^ is the universal gas constant and T = 298 K is temperature) and TΔS = ΔH − ΔG.

### Steady-state fluorescence spectroscopy

Steady-state fluorescence spectra have been acquired on a Fluoromax-4 spectrofluorometer (Horiba, Edison, NJ, USA) using a 1 cm path length quartz cuvette and at the fixed temperature of 10 °C. The excitation wavelength was set to 280 nm and the emission spectra were recorded in the range of 300–540 nm. The slits for the excitation and emission monochromators were set to 2 nm and 6 nm, respectively. Two μM of CtBP1/BARS was titrated with a solution of Comp. 11 ranging from 0 up to ~ 15 μM. The protein fluorescence was found to quench in the presence of the ligands. The binding curve was obtained by plotting *F/F*_*0*_ values versus ligand concentration, as described in details in [81].

The Comp.11 absorption at the excitation wavelength (280 nm) and at the emission maximum (344 nm) was not neglected. So, the integrated areas of the emission spectra were corrected using the following formula [82]:

$${F}_{abs}={F}_{corr}\times {10}^{\left(\frac{{A}_{ex }\times d}{2}+\frac{{A}_{em }\times d}{2}\right)}$$

where F_obs_ is the integrated area of the observed emission spectra, F_corr_ is the corrected integrated area, A_ex_ is the absorbance of Comp.11 at the excitation wavelength, A_em_ is the absorbance of Comp.11 at the emission maximum, and d is the cuvette path length.

### Circular Dichroism (CD)

CD spectra were performed on a Jasco J-1500 spectropolarimeter equipped with a Peltier temperature control system. Far UV-CD spectra were recorded at 20 °C using a 0.1 cm optical pathlength cell (Hellma, USA) with the following parameters: time constant of 2 s, 2 nm bandwidth, and a scan rate of 20 nm min^−1^. The final spectra are the result of three scan accumulations. The CD spectrum of the reference buffer was recorded and subtracted to the protein spectrum.

### [In silico calculations]

The structure of CtBP1/BARS has been refined with the Protein Preparation module embedded in Maestro suite, using the standard settings. The energetic minimization has been performed with the Macromodel Minimization module, using OPLS3e as force field; a value of 100 of force constant was set as constraints on all backbone atoms; Polak-Ribier Conjugate Gradient was selected as minimization method and the convergence threshold was set at 0.05.

The box for docking simulations was defined with the Receptor Grid Generation module, as embedded in Maestro suite, using the standard settings. Molecular docking calculations were performed with Glide Ligand Docking module; the first run of calculation using the Standard Precision mode, while the second one was performed in Extra Precision mode. For each docked compound, a post-docking minimization was carried out as previously described.

### Pull-down assay

Pull-down assays were carried out as described [64], with modifications.

For histidine pull-down with GST-CtBP1/BARS, 5 μg of GST–CtBP1/BARS was initially incubated with DMSO or increasing concentration of Comp.11 (5, 15 or 25 µ M) or 100 µ M acyl-CoA or 100 µ M NAD^+^ in His incubation buffer (50 mM Tris–HCl, pH 8.0, 150 mM NaCl, 20 mM imidazole, 0.2% Triton X-100, protease inhibitors) for 1 h, at 4 °C (rotating wheel). Then, equimolar amount of purified His–CtBP1/BARS (7.5 μg) was added for further 2 h, at 4 °C (rotating wheel). The samples were incubated with 50 μl Ni–NTA agarose beads (Qiagen) for 1 h, at 4 °C (rotating wheel). The beads were then washed three times with incubation buffer and eluted with elution buffer (50 mM Tris–HCl, pH 8.0, 150 mM NaCl, 250 mM imidazole, pH 8.0). Forty per cent of the eluted proteins were separated by 10% SDS–PAGE and analyzed by western blotting.

For histidine pull-down with LPAATδ, 5 μg of His–CtBP1/BARS was initially incubated with DMSO or 25 µ M Comp.11 or 100 µ M acyl-CoA or 100 µ M NAD^+^ in His incubation buffer for 1 h, at 4 °C (rotating wheel). Then, equimolar amount of immunoprecipitated LPAATδ was added for further 2 h, at 4 °C (rotating wheel). The samples were processed as described above.

For GST pull-down, 5 μg of His–CtBP1/BARS was initially incubated with DMSO or 25 µ M Comp. 11 or 100 µ M acyl-CoA or 100 µ M NAD^+^ or 5 mM MTOB or 50 μM HIPP or 5 μM PPγ in GST incubation buffer (20 mM Tris (pH 8.0), 1 mM EDTA, 0.2% Triton X-100, 100 mM KCl) for 1 h at 4 °C (rotating wheel). Equimolar amounts of GST-E1A (3 μg) or GST–14–3-3γ (5.5 μg) were added for further 2 h at 4 °C (rotating wheel). Then, 40 μl glutathione Sepharose beads were added for a further incubation for 1 h at 4 °C with gentle shaking. The beads were washed five times with GST incubation buffer and eluted from the glutathione Sepharose beads with GST elution buffer (100 mM Tris, pH 8.0, 20 mM glutathione, 5 mM dithiothreitol). Forty per cent of the eluted proteins were separated by 10% SDS–PAGE, and subjected to western blotting analysis.

### Size-exclusion chromatography

One milligram (1.5 mg/ml) of purified CtBP1/BARS dialyzed in gel filtration buffer (25 mM Tris, pH 7.4, 150 mM NaCl, 1 mM EDTA, 5% glycerol, 1 mM dithiothreitol) was incubated with DMSO, or 25 µ M of Comp.11, or 100 µ M of NAD^+^ (2 h, 4 °C, rotating wheel), or pre-incubated for 1 h with 100 µ M NAD^+^ and then for a further 2 h with 25 µ M Comp.11 at 4 °C (rotating wheel). The protein solution was then applied to a Sephacryl S-200 High Resolution HiPrep 16/60 (Amersham Pharmacia) gel filtration column equilibrated in gel filtration buffer. Fractions of 1 ml were collected using an AKTA FPLC system, applying a flow rate of 0.3 ml/min (Amersham Pharmacia). The eluted protein was detected by monitoring absorbance at 280 nm. Thirty-five microlitres of each fraction was separated on 10% SDS–PAGE gels and analysed by western blotting. One milligram of amylase (200 kDa) and alcohol dehydrogenase (158 kDa) were applied to the same Sephacryl S-200 column as molecular weight standards (Bio-Rad, Hercules, CA, USA).

### Cell Culture and Cell Cycle Synchronization

HeLa, A375MM and NRK cell lines were obtained from the American Type Culture Collection (ATCC). B16F10 cells were kindly provided by Prof. Fabio Mammano (from the Institute of Cell Biology and Neurobiology-CNR, Rome, Italy). NRK cells were cultured as indicated by the manufacturer. HeLa, A375MM and B16F10 cells were routinely cultured in MEM (supplemented with 100 μM MEM Non-Essential Amino Acids Solution), DMEM/F12 and RPMI1640, respectively, supplemented with 10% FBS and 2 mmol/L glutamine at 37 °C in a humidified 5% CO_2_ atmosphere. All cell culture reagents were from Thermo Fisher Scientific. The cultures p0-p10 were tested for *Mycoplasma* infection once a month and used in the following experiments.

NRK and HeLa cells were treated twice overnight with 2 mM thymidine; after each incubation with thymidine, the cells were washed once in sterile PBS and grown in the complete medium for 10 h (for NRK cells) and 14 h (for HeLa cells). The cells, were then fixed and stained with 2 μg/ml Hoechst. The mitotic index was evaluated under confocal microscope (Zeiss-LSM700). More than 200 cells were analyzed for each condition. Data are means ± SD of three independent experiments.

### Transport protocols, immunofluorescence and confocal microscopy

Immunofluorescence, VSVG or hGH-based assays were performed as previously described [64, 68, 83]. A375MM and B16F10 cells, treated as indicated, were grown on glass coverslips. The cells were then fixed in 4% paraformaldehyde for 10 min at RT, washed three times in PBS, and incubated for 30 min at RT in blocking solution (0.5% BSA, 50 mM NH_4_Cl in PBS, pH 7.4, 0.1% saponin and 0.02% sodium azide). Cells were subsequently incubated with the indicated antibodies diluted in blocking solution for 2 h at RT. Then, cells were washed three times in PBS and incubated with a fluorescent-probe-conjugated secondary antibody (1:400 in blocking solution) for 30 min at RT.

For the TGN-exit assay of VSVG, Comp.11 (15 μM) or MTOB (5 mM) or HIPP (50 μM) or PPγ (5 μM) treatments were performed during the VSVG TGN-exit assay for 1 h before the 32 °C temperature-release block and during the 32 °C temperature-release block. The cells were fixed at 0 and 30 min after the shift at 32 °C and then labelled with the anti-VSVG-Cy3 antibody.

For the hGH–GFP–FM transport assay, HeLa cells stably transfected with hGH-FM–GFP were treated with the DD-solubilizer at 37 °C to release the protein from the ER towards the PM. The treatment with Comp. 11 was performed 2 h before the addition of the DD solubilizer and during the protein release from the ER to the PM.

Following immunostaining, the cells were washed three times in PBS and twice in sterile water. The coverslips were then mounted on glass-microscope slides with Mowiol. Images were acquired using a Zeiss-LSM 700 confocal microscope with optical confocal sections at 1 Air Unit. The image analysis was performed using the open-source image processing software ImageJ2 (version 2.9.0, National Institute of Health, Bethesda, Maryland)),

### Transfections with siRNAs

A375MM or B16F10 cells were plated in six-well plates (0.5 × 10^5^/well) and grown for 24 h at 37 °C with 5% CO_2_. Then cells, at 60% confluence, were transfected with a non-targeting siRNA or with 150 nm of siGENOME SMARTpool of CtBP1 (M-008609–02, Dharmacon RNA Technologies) or CtBP2 (M-008962–03, Dharmacon RNA Technologies) or LPAATδ (M-009283–01, Dharmacon RNA Technologies) siRNAs using the RNAiMAX reagent, according to the manufacturer’s instructions. Forty-eight hours after transfection, cells were processed for total RNA or protein extraction. The efficiency of interference was assessed by Western blot or RT-PCR.

### *In vitro* acyltransferase assay

*In vitro* acyltransferase assays were carried out as previously described [68]. A374MM cells (1.5 × 10^6^) in 10-cm Petri dishes were transiently transfected with 6 µ g plasmid DNA encoding Flag-LPAATδ^wt^ for 48 h (using Lipofectamine LTX/plus reagent) and then treated with 25 µ M Comp.11 for 2 h. Alternatively, the A375MM cells were transfected with LPAATδ or CtBP1/BARS siRNAs for 48 h (using RNAiMAX reagent). Then, cells were washed, harvested in the homogenization buffer (100 mM Tris, pH 7.4, 5 mM NaCl, 3 mM MgCl_2_) and homogenized. The lysate was centrifuged at 600 × g for 10 min at 4 °C, and 3 μg of the post-nuclear supernatant fraction was used in the acyltransferase assay. After LPAAT reaction, the lipids were extracted and separated by running the TLC plates. The radiolabeled lipids were analyzed using a RITA® TLC Analyser (Raytest, Germany) and quantified using GINA® (Raytest, Germany) software analysis. For each analyzed sample, nmols of phosphatidic acid produced were calculated using dioleoyl [^14^C]-PA (Perkin Elmer) as an internal standard.

### Cell viability assay

A375MM or B16F10 cells (5 × 10^3^/well) were seeded onto 96-well plates and cultured as described above. After 24 h of incubation, cells were incubated with complete medium in the presence of Comp.11 at different concentrations (from 0 to 150 μM) for 24, 48 and 72 h. MTT (5 mg/ml) was added and incubated for further 4 h at 37 °C. Then, DMSO was added at 150 μl/well, and incubated for 30 min to dissolve the formazan completely. Absorbances were determined at 570 nm using a plate reader (Cytation 3 BioTek Instruments, Winooski, VT, USA). Blanks containing medium only was used to correct the absorbances. The mean optical density (OD, absorbance) of four wells in the indicated groups was used to calculate the percentage of cell viability as follows: percentage of cell viability = (A_treatment_ − A_blank_) × 100 (where, A = absorbance 570 nm). Three independent sets of experiments were analyzed. The EC_50_ values of Comp.11 at each time point for each cell line were calculated by GraphPad Prism 7.0 (GraphPad Software).

### Flow Cytometry

A375MM or B16F10 cells were seeded onto 10-cm Petri dishes (1 × 10^6^ cells). After overnight incubation at 37 °C, the cells were treated with DMSO (vehicle control) or 15 µ M Comp.11 for 24 h or transfected with CtBP1/BARS or CtBP2 or non-targeting siRNAs for 48 h. Trypsinized cells were pelleted, washed in ice-cold PBS, resuspended in ice-cold ethanol (while vortexing) and then incubated overnight at -20 °C. Then, the samples were centrifuged at 500 × g for 5 min, the ethanol was removed, and the cells were washed in ice-cold PBS and incubated with 50 μg/ml propidium iodide for 30 min in the presence of 200 μg/ml RNAse A. The cells were then analyzed using the Becton Dickinson (BD) FACSCantoA instrument. Data are means ± SD of three independent experiments.

### Apoptotic assay

Apoptosis-mediated cell death of melanoma cell lines was examined at 24 h following Comp.11 treatment (15 µ M) and 48 h following siRNAs transfection by double staining with the Annexin V-FITC kit (Miltenyi Biotec GmbH, Bergisch Gladbach, Germany), according to the manufacturers’ instructions. Data are means ± SD of three independent experiments.

### Wound-Healing assay using the IBIDI culture insert

An Ibidi Culture Insert (Ibidi, Germany) consists of a chamber with two reservoirs separated by a 500 µm thick wall sticked to the surface of a 35 mm dish. An equal number of A375MM or B16F10 cells (70 µl; 5 × 10^5^ cells) was added into the two reservoirs of the same insert and incubated at 37 °C. After overnight, the cells were treated as indicated and the insert was gently removed creating a gap of ∼500 µm. The wound closure was monitored over the next 16 h, and images were captured by using AxioVision microscope (Carl Zeiss Micro Imaging GmbH) and were evaluated by AxioVision 4.2 software. The wound width was calculated by measuring the mean distance between the edges of the wound at time t0 (time of wounding) and t16 h (16 h after wounding) in randomly selected fields on the images by using ImageJ2. Three independent wound-healing assays were performed for each experimental condition.

### Cell invasion assay

Cell invasion assays were performed using transwell chamber (Merck, Darmstadt, Germany). Matrigel (Merck, Darmstadt, Germany) was diluted to 5 mg/ml with serum-free medium, and applied to 8-µm pore size polycarbonate membrane filters of the chamber at 37 °C for 1 h. The cells (1 × 10^5^/well) were treated as indicated and then seeded to the upper part of the chamber in serum-free medium. In the lower chamber, medium containing 10% FBS served as a source of chemo attractants. After 16 h, the cells on the surface of the upper membrane were removed. The cells that had invaded through the Matrigel and penetrated the insert were fixed in 4% paraformaldehyde solution supplemented with Hoechst 33342. The numbers of invasive cells were determined from ten random fields using a fluorescence microscope.

### Colony formation assay

For anchorage dependent colony assay, A375MM cells were seeded into 6 well plates (5 × 10^3^/well). The cells were cultured in complete medium containing DMSO (vehicle control) or Comp.11 (5 or 15 μM) or MTOB (5 mM) or HIPP (50 μM) or PPγ (5 μM) for 48 h. Then, the cell culture medium was replenished with fresh medium in the presence of the inhibitors every day and incubated for 7 days until large colonies were formed. Next, the cells were washed twice with PBS, fixed with 4% paraformaldehyde for 20 min, stained with crystal violet (0.5%) for 15 min, washed with water, and visualized under a phase-contrast light microscope. Cells were then lysed in 33% acetic acid to solubilize the dye, and the colony formation was evaluated by the optical density measured at 590 nm using a plate reader. Two independent assays were carried out in triplicate.

For anchorage independent colony formation assay, A375MM cells (5 × 10^3^/well) were seeded in 12 well plates with a bottom layer of 0.6% agarose (16500–500, Thermo Fisher Scientific) in DMEM/F12 plus 10% FBS and a top layer of 0.3% agarose in DMEM/F12. The wells were allowed to solidify, and 300 µl of growth medium supplemented with DMSO (vehicle control) or Comp.11 (5 or 15 μM) was added on top and refreshed every day to avoid agar drying. After 3 weeks, the top layer was removed and colonies were stained with Nitro Blue Tetrazolium Chloride dye (Thermo Fisher Scientific, N6876); scanned, and counted using ImageJ. Two independent assays were carried out in triplicate.

### Western blotting

A375MM and B16F10 cells were processed as previously described [68]. Briefly, the cells treated as indicated were washed three times with ice-cold PBS and scraped on ice in lysis buffer (25 mM Tris, pH 7.4, 150 mM NaCl, 5 mM EDTA, 5 mM MgCl_2_, 10 mM NaF, 40 mM β-glycerophosphate, 1 mM Na_3_VO_4_, 1 mM dithiothreitol, 1% Triton X-100) supplemented with complete protease inhibitor cocktail (Roche, Basel, Switzerland). Fifty micrograms of total protein extracts were separated by SDS–PAGE, and analyzed by western blotting.

The nitrocellulose membranes were blocked for 1 h at RT in Tris-Buffered Saline (TBS; Bio-Rad Laboratories, Hercules, CA, USA) with 0.05% Tween-20 (TBS-T; Merck, Darmstad, Germany) containing 5% BSA (Merck, Darmstad, Germany). The primary antibodies were diluted in blocking solution and incubated with membranes overnight at 4 °C. After washing with TBS-T, the membranes were incubated with an appropriate HRP-conjugated secondary antibody diluted in 5% non-fat, dry milk in TBS-T for 30 min at RT. The membranes were washed extensively with TBS-T before chemiluminescent detection using the ECL Western Blotting Detection Reagents (Merck, Darmstad, Germany; Cytiva, Marlborough, MA, USA) and X-ray film (Fujifilm, Tokyo, Japan).

### RNA extraction and real-time PCR

Total RNAs were extracted using the RNeasy Mini Kit (Qiagen, Hilden, Germany). cDNAs were obtained with QuantiTect Reverse Transcription kits (Qiagen, Hilden, Germany). according to the manufacturer’s protocol. Real-time PCRs were performed using 10 ng of cDNA, 50 nM concentration of each primer (listed in Tables S1 and S2), and SYBR Green Master Mix (Applied Biosystems) in 20-μl reactions in a Light Cycler 480 II thermocycler (Roche). mRNA levels were normalized to the internal housekeeping gene GAPDH. To measure the fold change of expression levels between control and experiment(s), the ΔΔ method (2^−ΔΔCT^) was used. Three biological replicates were analyzed and the average and SD were calculated.

### Animal care

The mice were maintained on a purified standard diet (4RF21) from Mucedola (Settimo Milanese, Milan, Italy). They were housed at a maximum of four mice per cage, and maintained at a constant temperature (22 ± 2 °C) and humidity (45–65%) on a 12-h light/dark cycle, with food and water ad libitum. The mice were monitored daily by a veterinary, to be sure of their health and behavioral status.

### In vivo xenograft studies

All studies have been performed in compliance with institutional guidelines and regulations (Directive 2010/63/EU; Italian Legislative Decree DLGS 26/2014) and after approval from the appropriate institutional review board and Italian Ministry of Health (authorization number 678/2017-PR). Female CD1 nude mice (Charles River, Wilmington, MA, USA) were used for A375MM xenograft model. Mice were acclimatized in the Animal Care Facility of CROM (laboratori di Mercogliano)- “Fondazione G. Pascale” – IRCCS. After seven days, cells (2,5 × 10^6^) diluted in 200 μl PBS were injected subcutaneously (s.c.) in the flank regions of the mice. When the tumors became palpable, the mice were randomized into three experimental groups (*n* = 3) to receive Comp.11 at the dosages 10 mg/Kg (daily. for 2 weeks), 20 mg/kg (three times/week for 2 weeks) or its vehicle intraperitoneally administrated for 14 days. Comp.11 (10 or 20 mg/kg/day dissolved in 10% DMSO/45% PEG/45% physiologic solution). Mice in the control groups were treated with vehicle (10% DMSO/45% PEG/45% physiologic solution) only. Tumor volume [(TV) (mm^3^)] and the percent change in the experimental groups was compared with that of the vehicle control groups as described before [84]. The measurement for each single mouse at the indicated time point was compared with the respective values at day 1 (T0). Relative fold increase values were reported in the graph.

Furthermore, we performed additional in vivo experiment using high-frequency ultrasound (HFUS) system, mounting a 40 MHz transducer (VEVO 2100, FUJIFILM VisualSonics, Inc., Toronto, 270 Ontario, Canada), to evaluate tumor growth in 3 Balb/c nude mice for each treatment group (vehicle and Comp.11) as above described (imaging study was approved by the Italian Ministry of Health, authorization number 932/2018-PR and 38/2015-PR). The HFUS evaluations were conducted in anesthetized mice (2% isoflurane in 100% oxygen at 0.8 L/min). Each mouse was placed in the right lateral recumbence on a dedicated small animal table (VEVO Imaging Station 2, FUJIFILM 274 VisualSonics, Inc., Toronto, Ontario, Canada). Brightness (B-) mode images were obtained for each tumor in two orthogonal planes, *i.e.*, the trans-axial and the sagittal planes. All tumors were scanned both on the transverse and on the sagittal plane, and height, width and length were recorded. Tumor volumes (TVs, in mm^3^) were calculated according to the ellipsoid formula: (height × width × length)/(π/6).

### Statistical analysis

Data are presented as mean ± standard deviation (SD). Statistical analyses were performed using Student's *t*-test (unpaired or paired, for data derived from the in vitro and in vivo experiments, respectively). All analyses were performed using GraphPad Prism Software 7.0 (GraphPad Software Inc., San Diego, CA, USA). Significance is indicated as *P ≤ 0.05, **P ≤ 0.01 and ***P ≤ 0.001.

## Results

### Identification of small molecules targeting CtBP1/BARS

In order to identify novel molecules that control the conformational arrangement and the cellular functions of CtBP1/BARS, we performed a virtual screening campaign targeting the NADH-binding Rossmann fold region as a binding pocket for the docking simulations. This region was further extended to include a sub-pocket nearby, where inhibitors such as MTOB, PPγ and HIPP [32, 71, 72, 85] are known to locate and exert their activity, in order to explore both competitive and non-competitive binding scenarios [71].

**Fig. 1:**
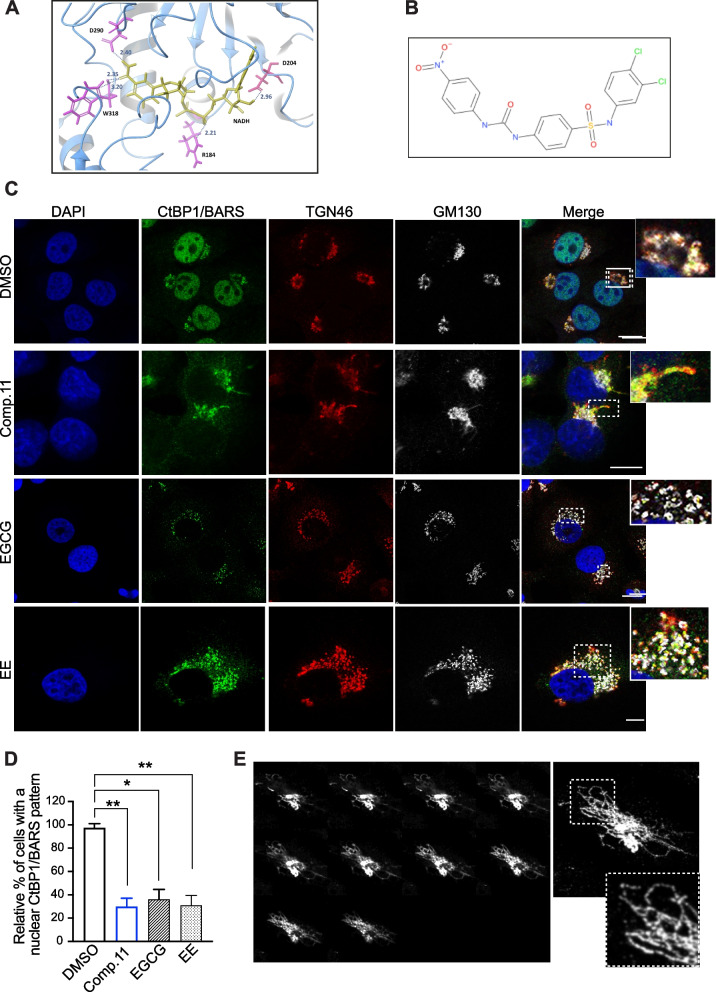
Identification of small molecules targeting CtBP1/BARS and their cellular effects. **A**, Detail of the co-crystalized complex of NADH (yellow) and CtBP1 (residues are referred to CtBP1-L; light blue ribbon) (PDB 1MX3). Crucial residues stabilizing the complex are reported in pink; interaction and distances between the protein functional groups and NADH are annotated in Armstrongs. **B** Chemical structure of N-(3,4-dichlorophenyl)-4-{[(4-nitrophenyl) carbamoyl]amino}benzene-sulfonamide (referred as Comp.11 from here on). **C** Representative confocal microscopy images of A375MM cells treated with DMSO (vehicle control) or with Comp.11 (15 μM) or EGCG (15 μM) or EE (50 μM) for 2 h at 37 °C (as indicated). Cells were fixed and labeled with a polyclonal anti-CtBP1/BARS antibody (endogenous CtBP1/BARS; green), with an anti-TGN46 antibody (used as a TGN-Golgi marker; red) and with a monoclonal anti-GM130 antibody (used as a cis-Golgi marker; grey). Insets, right: Magnification of Golgi area. Scale bars, 10 μm. **D** Quantification of cells with a nuclear localization pattern of CtBP1/BARS. Data are means ± SD of three independent experiments. *P ≤ 0.05, **P ≤ 0.01 *versus* DMSO (vehicle control) (Student’s t-tests). **E** Projections of Z-stack confocal microscopy images of A375MM cell treated with Comp.11, fixed and labeled with anti-TGN46 antibody (grey). Insets, right: Magnification of the elongated TGN tubular membranes

The resolved structure of human CtBP1/BARS (PDB: 4LCE) was selected since co-crystallized with both NADH and the inhibitor MTOB, thus offering clear indications about the complete binding region and the crucial residues involved in the ligand–protein interaction. Therefore, a single monomer of CtBP1/BARS was targeted during the simulations (Fig. 1A).

The protein structure was prepared for the docking calculations by *i)* refining and optimizing the overall backbone and residue side chains, *ii)* removing NADH, MTOB and solvent molecules, *iii)* relaxing the residues included into a box of 30 Å^3^, centered on the MTOB center of mass, (so to include both MTOB and NADH environments), via energetic minimization.

A subset of the KEGG Library, accounting for 9.000 small molecules, natural products and metabolites [86] long with a selection of 50.000 commercially available compounds was selected as compound database for the virtual screening campaign. A two-step docking strategy was conducted. In the first run, performed at a standard level of details, the best 2.000 compounds were selected according to the docking score; in the second run, carried out at higher precision with more stringent and time-demanding settings to optimize binding mode and predicted affinity scores, a definitive list of candidates was selected. All in silico calculations, from structure and ligand refinement to docking simulations were performed using the Schrödinger Drug Discovery suite [87] while the entire flow of activities was concerted and monitored with Biovia Pipeline Pilot [88].

Among the top ranked predicted hits, 27 commercially available molecules were acquired and firstly tested for their capability to affect the intracellular localization of CtBP1/BARS in the human A375MM melanoma cell line (a cancer type in which the CtBP1/BARS expression levels are more pronounced than in other tumors; Supplementary Fig. 1A) by immunofluorescence microscopy.

The endogenous CtBP1/BARS localizes both in the nucleus and in the Golgi complex where it acts as transcriptional corepressor and membrane fission inducer, respectively [35, 36, 64]. Three molecules are the most active (at low micromolar concentrations) in promoting the translocation of CtBP1/BARS from the nucleus into the cytoplasm and Golgi membranes, namely; (-)-Epigallocatechin gallate (EGCG), ethinyl estradiol (EE) and N-(3,4-dichlorophenyl)-4-{[(4-nitrophenyl)carbamoyl]amino}benzene-sulfonamide (referred as Comp.11 from here on) (Fig. 1B-D). Similar cytoplasmic re-localization of CtBP1/BARS has been observed in murine melanoma B16F10 cells (Supplementary Fig. 1B) and in other tumors: cervical (HeLa cells) and breast (MCF7 cells) (Supplementary Fig. 2), both tumors transcriptionally regulated by CtBP1/BARS as well. Surprisingly, while EGCG and EE promoted Golgi fission fragmentation (presumably inducing the monomeric fission-prone conformation of CtBP1/BARS) **(**Fig. 1C**)**, Comp.11 induces Golgi membrane tubulation in all the cancer cell lines (Fig. 1C and 1E, Supplementary Fig. 1B and Supplementary Fig. 2) that resembles a membrane fission defect seen upon inhibition of CtBP1/BARS monomerization [35, 64, 68].

Thus, Comp.11 seems to inhibit both CtBP1/BARS functions: the nuclear transcriptional activity (through protein redistribution into the cytoplasm and in the Golgi Complex) as well as its fission activity (Golgi membrane fission defect, see Fig. 1C and 1E, Supplementary Fig. 1B and Supplementary Fig. 2). This might be due to conformational changes on CtBP1/BARS induced upon Comp.11 binding, and this combination might be very effective in both the transcriptional pro-survival effects and cell cycle control of CtBP1/BARS. Hence, we focus our further investigation on Comp.11.

### Thermodynamic characterization of the Comp.11 - CtBP1/BARS binding

**Fig. 2:**
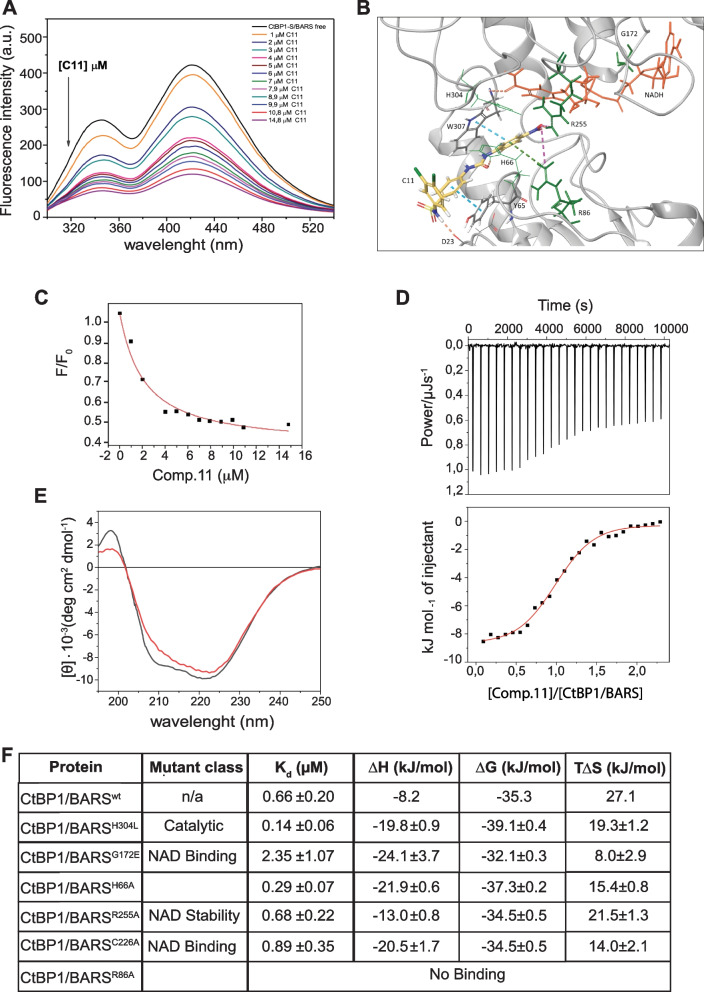
Thermodynamic characterization of the Comp.11-CtBP1/BARS binding. **A**. Fluorescence emission spectra of 2 μM solution of purified CtBP1/BARS (black line) and the tryptophan fluorescence quenching effect (black arrow) upon addition of increasing concentration of Comp.11 (C11, colored lines) on its fluorescence intensity. **B** Detail of the Comp.11 predicted binding mode in CtBP1/BARS, as computed in silico; Comp.11 (C11, yellow) and NADH (orange) coexist within the Rossmann Fold. Crucial residues for the interactions are highlighted (in sticks); in green, those residues selected for experimental validation via mutagenesis. The interactions between Comp.11 and CtBP1/BARS functional groups are schematized: blue dotted lines represent π- π interactions; green dotted lines stay for cation-aromatic interactions; pink dotted lines for salt bridge and orange dotted lines represent H-bonds. **C** Binding curve of CtBP1-S/BARS titration *versus* Comp.11 concentration (*F/F0*; see Methods). The red line represents the model fit to a binding equilibrium (K_a_ = 2,1 ± 0,6 × 10^5^ M^−1^) obtained by static quenching model [90] and OriginPro 9 software from the experimental points (black squares). **D** Top: ITC trace for the titration of the protein CtBP1/BARS with the Comp.11 as ligand. Bottom: Integrated ITC data (black squares) as a function ligand/protein concentration ratio. The solid red line is the best fit of experimental data using the independent site binding model. **E** Far UV-CD spectra of CtBP1/BARS in the absence (black line) and in presence of Comp.11 at protein ratio 1/1 (red line). The CD spectra were normalized and reported in mean residue ellipticity (MRE), represented by the symbol [θ]mrw (deg cm^2^ dmol.^−1^) and plotted as a function of the wavelength (see Methods). **F** Thermodynamic parameters obtained by ITC measurements for binding of Comp.11 to wild-type or point mutants of CtBP1/BARS (as indicated)

To examine the binding of Comp.11 in the NADH-binding Rossmann fold of CtBP1/BARS we performed fluorescence measurements taking advantages of the peculiar intra-molecular FRET between the residue W307 and the NADH moiety bound to the Rossmann fold of CtBP1/BARS [89] (Fig. 2A). Here the distance between W307 and the nicotinamide moiety of NADH for energy transfer is within the Förster radius of 25 Å [89]; thus, upon CtBP1/BARS excitation at 285 nm, the W307 acts as fluorescence donor to NADH that absorbs at 340 nm (the maximum emission of tryptophan) to emit fluorescence signal at ≅ 425 nm (Fig. 2A, black line). This spectrum agrees with the structural information that NADH binds the Rossmann fold of CtBP1/BARS [39], which, in turn, is in its NADH-bound dimeric conformation [39, 67].

Titration with increasing concentration of Comp.11 (0–15 μM) with fixed CtBP1/BARS (1 μM) was used to monitor the capability of this compound to displace NADH from the Rossmann fold. The addition of Comp.11 reduces both the tryptophan fluorescence peak at 344 nm and the NADH fluorescence peak at 425 nm, in a dose-dependent manner (Fig. 2A, color lines). These data indicate that Comp.11 does not release/displace NADH from the Rossmann fold (which should instead result in loss of FRET signal and accompanied by decreased NADH peak and increased tryptophan peak). These data confirm Comp.11 predicted binding mode.

As schematized in Fig. 2B, Comp.11 is expected to locate near the NADH molecule, the way MTOB, PPγ and HIPP do [32, 71, 72, 85], projecting the nitrobenzene moiety towards the center of the binding pocket and the nicotinamide moiety of NADH. Here, the nitro group is stabilized by a salt bridge with the guanidine group of R255 and an H-bond with another arginine, R86. Comp.11 orientation is further strengthened by the π-π stacking and the π-cation stacking that the nitrobenzene ring forms with the aromatic side chain of W307 and positive charge of R86, respectively. At the other extremity of Comp.11, its benzene sulfonamide moiety stacks with the aromatic ring of Y65 and establishes an H-bond with carbonyl backbone of D23, stabilizing the complex with CtBP1/BARS. The binding constant (K_b_) of the resulting Comp.11–CtBP1/BARS complex quantified based on static quenching methodologies [90] is K_b_ = 2.1 ± 0.6 × 10^5^ M^−1^ (Fig. 2C) corresponding to a K_d_ of 4.7 ± 1.3 µM. Further, we employed isothermal titration calorimetry (ITC) to determine the thermodynamic parameters associated to the binding of Comp.11 to CtBP1/BARS (Fig. 2D). We found that one molecule of Comp.11 binds to the protein, with a dissociation constant of K_d_ = 0.66 ± 0.20 μM (three orders of magnitude tighter than MTOB, K_d_ = 1.26 mM) [85]. The measured negative value of the binding enthalpy, **∆**H = -8.2 kJ/mol and the positive entropy value, T∆S = 27.1 kJ/mol, suggest that the interaction of Comp.11 is both enthalpically and entropically driven. These results agree with the above computational data, as the negative value of the enthalpy take into account the stabilizing interaction of the ligand and protein; while the water release from the binding site induced by the mostly hydrophobic Comp.11 explain the positive value of the binding entropy. A selection of single point mutants of CtBP1/BARS was also included in the panel in order to explore the contribution of critical (known and presumed) residues to the binding. Some of these residues were selected since they are crucial for NADH binding [G172E, C226A; [39, 67, 91]] and stability [R255A, [91]]; other being predicted to interact with Comp.11 [H66A, R86A; [91]]. Finally, H340L mutant was chosen because H340 plays an important role in the catalytic activity [91]. Related binding isotherms are shown in Fig. 2D and in Supplementary Fig. 3, and summarized with the thermodynamic parameters in Fig. 2F.

As predicted in silico and confirmed via ITC, Comp.11 binding strongly relies on R86, and substitution of this residue completely compromises the establishment of the complex of Comp.11 with CtBP1/BARS. Besides, the substitution of the two histidines, H66 and H304, with hydrophobic residues, alanine and leucine, respectively, would probably improve the binding (lower measured K_d_) by enriching the apolarity of the region surrounding the nitrobenzene moiety. Moreover, despite the predicted salt bridge between the nitro group and the side chain of R255, its substitution with alanine does not affect the binding affinity, probably because Comp.11 overall orientation within the pocket is guaranteed by R86. Finally, the G172 mutation leads to the dramatic decrease in the affinity of Comp.11–CtBP1/BARS interaction and we think that this effect does not correlate directly with a loss of direct interaction. We can assume that the presence of NADH itself (as found for MTOB binding), compromised in the G172E mutant [67], is a crucial step for Comp.11 binding.

Although we demonstrated that the binding of Comp.11 to CtBP1/BARS results in the fluorescence quenching of CtBP1/BARS (Fig. 2A), it is still unclear whether the binding affects the structure and the microenvironment of the protein. Therefore, we performed circular dichroism (CD) experiments to further investigate the conformational changes of CtBP1/BARS. This protein (at 2 μM concentration, 20 °C) in buffer solution shows a well-structured conformation as indicated by the negative bands at 222 nm and 208 nm and the positive band below of 200 nm (Fig. 2E). In the presence of Comp.11 at ratio 1/1 of protein/ligand, the band at 208 nm is slightly reduced, (Fig. 2E) indicating that the protein secondary structure is preserved upon ligand binding.

#### Comp.11 affects the oligomerization of CtBP1/BARS and inhibits the binding of CtBP1/BARS to partners involved in both transcription and membrane fission

**Fig. 3:**
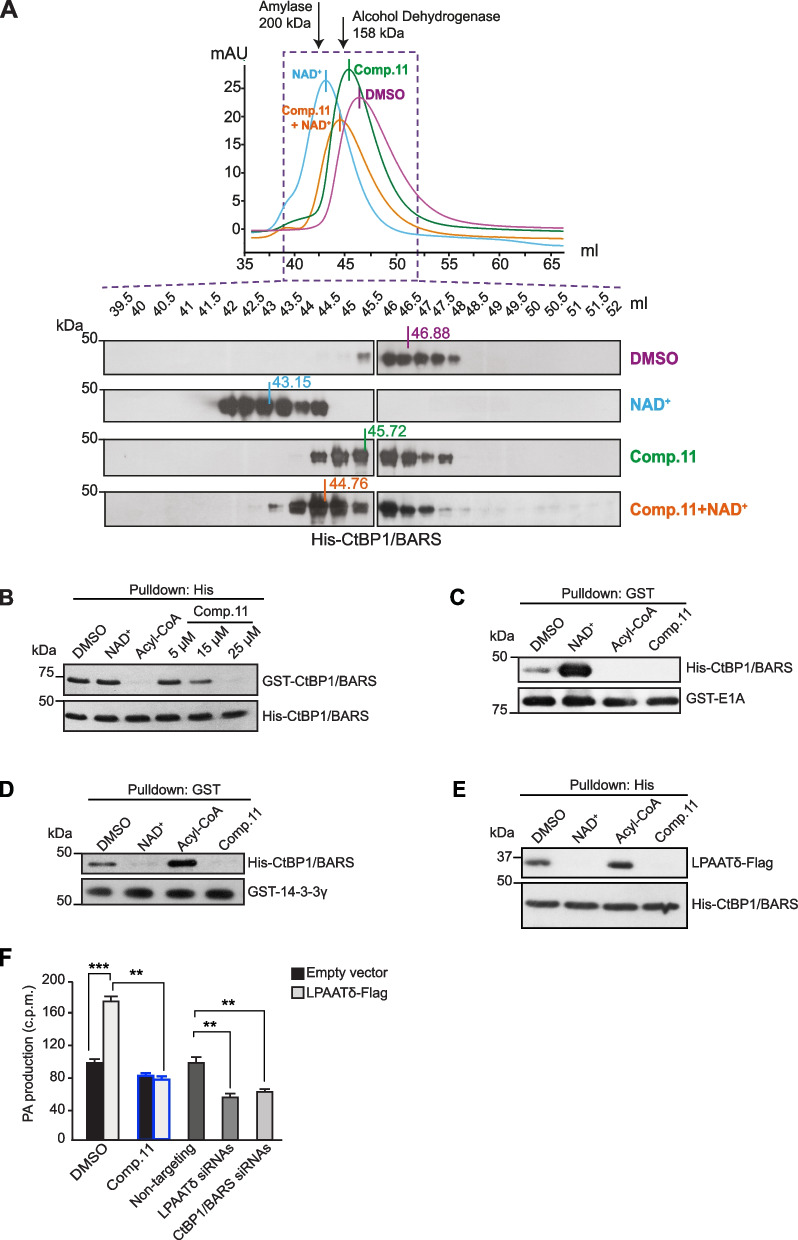
Comp.11 affects the oligomerization state of CtBP1/BARS and the interaction of CtBP1/BARS with transcription and membrane fission partners. **A** Top: Size-exclusion chromatography profiles of wild-type CtBP1/BARS incubated with DMSO (vehicle control, 2 h, 4 °C; purple line) or with NAD^+^ (100 μM, 2 h, 4 °C; blue line) or with Comp.11 (25 μM, 2 h, 4 °C; green line) or pre-incubated with NAD^+^ (1 h, 100 μM) and then for a further 2 h with Comp.11 (25 μM, 4 °C; orange line) (see Methods). One mg of purified CtBP1/BARS protein was applied to the Sephacryl S-200 column, with Gel filtration elution buffer at 0.3 ml/min. The elution patterns were detected by monitoring the absorbance at 280 nm. The elution positions of the molecular weight markers used are indicated by arrows: Amylase (200 kDa), alcohol Dehydrogenase (158 kDa). Bottom: Western blotting with anti-CtBP1/BARS antibody of aliquots of each eluted fraction (see Methods). **B** Representative histidine pull-down of equimolar amounts of His-CtBP1/BARS recombinant protein for GST-CtBP1/BARS. His-CtBP1/BARS was first incubated with DMSO (vehicle control), or 100 µ M NAD^+^ or 100 µ M acyl-CoA or increasing concentration of Comp.11 (5, 15 and 25 µ M) (1 h, 4 °C in a wheel), and then incubated with purified GST-CtBP1/BARS protein (2 h, 4 °C in a wheel; see Methods). The eluted proteins were analyzed by Western blotting using anti-GST monoclonal antibody (Top), with the pulled down His-CtBP1/BARS revealed with anti-His monoclonal antibody (Bottom). **C** Representative GST pull-down of GST-E1A recombinant protein for His-CtBP1/BARS. His-CtBP1/BARS protein was first incubated with DMSO (vehicle control) or with 100 μM NAD^+^ or 100 μM acyl-CoA or 15 μM Comp.11 (1 h, 4 C in a wheel), and then incubated with GST-E1A (2 h, 4 C in a wheel; see Methods). The bound proteins to the glutathione Sepharose beads were eluted and analyzed by Western blotting (top, anti-His monoclonal antibody; bottom, anti-GST monoclonal antibody). **D** Representative GST pull-down of GST-14–3-3γ recombinant protein for His-CtBP1/BARS. His-CtBP1/BARS protein was pre-incubated with DMSO (vehicle control) or with 100 μM NAD^+^ or 100 μM acyl-CoA or 15 μM Comp.11 (1 h, 4 °C in a wheel), and then incubated with GST-14–3-3γ (2 h, 4 °C in a wheel; see Methods). The bound proteins to the glutathione Sepharose beads were eluted and analyzed by Western blotting (top, anti-His monoclonal antibody; bottom, anti-GST monoclonal antibody). **E.** Representative His pull-down of His-CtBP1/BARS beads for LPAATδ-Flag immunopurified from lysates of A375MM cells overexpressing LPAATδ. His-CtBP1/BARS beads were treated with DMSO (vehicle control) or with 100 µ M NAD.^+^ or 100 µ M acyl-CoA or 15 µ M Comp.11 (1 h, 4 °C in a wheel), and then incubated with the immunopurified LPAATδ-Flag (2 h, 4 °C in a wheel; see Methods). The eluted proteins were analyzed by Western blotting (top, anti-Flag monoclonal antibody; bottom, anti-His monoclonal antibody). Molecular weight standards (kDa) are indicated on the left of each panel. Data are representative of three independent experiments.** F.** Quantification of phosphatidic acid (PA) production in the LPAAT assay for post-nuclear supernatants from A375MM cells transfected for 48 h with an empty Flag-vector or with LPAATδ-Flag and incubated with 15 μM Comp.11 or with DMSO (vehicle control) for 30 min at 25 °C before LPAAT assay, or alternatively transfected for 72 h with non-targeting or LPAATδ or CtBP1/BARS siRNAs. Data are means ± SD of three independent experiments. ***P ≤ 0.001 *versus* DMSO, **P ≤ 0.01 *versus* non-targeting (Student’s t-tests)

To investigate the effects of Comp.11 on the oligomerization state, size-exclusion chromatography was performed using purified CtBP1/BARS protein. Full-length CtBP1/BARS in the absence of NAD^+^ elutes primarily as a dimer mediated by the C-terminal domain (as shown in [39, 67]) near its predicted *M*_*r*_ 96 kDa (Fig. 3A; see DMSO purple line and Ctr/DMSO Western blot panel). The addition of NAD^+^ shifts the elution patterns so that the majority of CtBP1/BARS elutes as a tetramer, as expected, near its predicted *M*_*r*_ 192 kDa (Fig. 3A; see NAD^+^ blue line and NAD^+^ Western blot panel). After the addition of Comp.11, in the absence and in the presence of NAD^+^, CtBP1/BARS dimerizes and tetramerizes, respectively, in a slightly less packed conformation (Fig. 3A; see Comp.11 green and Comp.11 + NAD^+^ orange lines and Western blot panels). This indicates that the binding of Comp.11 to CtBP1/BARS alters its oligomerization by favoring an “open oligomeric conformation” probably as a result of a partial hindrance due to the interaction of dichlorophenyl-benzenesulfonamide moiety of Comp.11 with the second CtBP1/BARS monomer during the dimerization/tetramerization process. As reported in Supplementary Fig. 4 this portion of Comp.11 would present at the dimerization interface, in an area normally occupied by a stretch of CtBP1/BARS spanning from residues 153 to 168, somehow impeding the closest approaching of the two monomers.

To define the mechanism through which the binding of Comp.11 to CtBP1/BARS affects the cellular functions of this protein we first investigated whether Comp.11 has an impact on the dynamic equilibrium between monomeric and dimeric conformations of CtBP1/BARS by in vitro pull-down assay with purified recombinant proteins. The preincubation with Comp.11 impaired, in a concentration dependent manner, the ability of GST-CtBP1/BARS to bind to, and dimerizes to, His-CtBP1/BARS immobilized on Ni–NTA–agarose beads (Fig. 3B). NAD^+^ and acyl-CoA were used as internal pull-down controls: NAD^+^ to promote the GST-CtBP1/BARS–His-CtBP1/BARS self-association/dimerization and acyl-CoA to inhibit it (Fig. 3B).

After that, we further performed in vitro pull-down experiments, to directly assess whether Comp.11 can alter CtBP1/BARS interactions with the well-known molecular partners involved in transcriptional corepressor activity such as E1A (Fig. 3C) [92, 93] or in membrane fission (Fig. 3D,E) [63, 64, 68]. Figure 3C shows that the preincubation with Comp.11 (as well as with Acyl-CoA) inhibits the ability of His-CtBP1/BARS to bind to GST-tagged E1A, while the addition of NAD^+^ stabilizes their interaction as previously reported [35, 39].

We demonstrated that CtBP1/BARS, at the *trans*-Golgi network, is incorporated into a protein complex where the binding to 14–3-3γ adaptor protein stabilizes the monomeric fission-prone conformation of CtBP1/BARS [63, 64], which, in turn, binds to and activates a lysophosphatidic acid (LPA) acyltransferase type δ (LPAATδ), and this LPAATδ -mediated production of phosphatidic acid (PA) is required for fission of post-Golgi carriers [64, 68, 94].

Based on these data, we have investigated the effect of Comp.11 binding to CtBP1/BARS on the assembly and function of the above complex. We first found that Comp.11 inhibits the ability of His-CtBP1/BARS to bind to GST-tagged 14–3-3γ (Fig. 3D) and to Flag-LPAATδ (immunopurified from lysates of HeLa cells transiently-transfected with Flag-tagged LPAATδ, Fig. 3E). Then, on the same line of evidence, CtBP1/BARS bound to Comp.11 was not able to activate the enzymatic activity of LPAATδ and hence the production of PA (Fig. 3F). To this end we performed an in vitro LPAATδ acyltransferase assay [as described in [68]] where extract from Flag-LPAATδ expressing A375MM melanoma cells were incubated with the acyl donor [1-14C]-oleoyl-CoA and the acyl acceptor oleoyl-LPA, with [1-14C]-PA measured as the reaction product (Fig. 3F). The 45% increase in LPAATδ activity in extract from LPAATδ overexpressing cells over the empty Flag-vector transfected cells was completely hindered by Comp.11 (Fig. 3F) which confirms its inhibitory effects on assembly and function of the CtBP1/BARS-mediated fission machinery. Extracts from LPAATδ- and CtBP1/BARS-depleted cells, where LPAATδ is inactive (as described in [68]) were used as internal controls of the LPAATδ acyltransferase assay. As a control of specificity, Comp.11 treatment did not affect the cellular levels of LPAATδ (Supplementary Fig. 5). Finally, as a control of selectivity, the reported CtBP inhibitors, namely MTOB, HIPP and PPγ were unable to inhibit the binding of CtBP1/BARS to both E1A and 14–3-3γ interactors (Supplementary Fig. 6).

Collectively, these results indicate that Comp.11 after binding to CtBP1/BARS induces an oligomerization change that inhibits the ability of CtBP1/BARS to bind both its transcription and membrane fission partners.

#### Comp.11 impairs the CtBP1/BARS-controlled protein transport and cell entry into mitosis

We have previously reported that CtBP1/BARS localizes at the Golgi complex where it controls membrane fission required to support: *i)* the export of specific class of basolateral cargoes (e.g., human growth hormone (hGH) and vesicular stomatitis virus G protein (VSVG); and *ii)* the Golgi ribbon unlinking during G2/M transition and hence the cell entry into mitosis [63, 68].

**Fig. 4:**
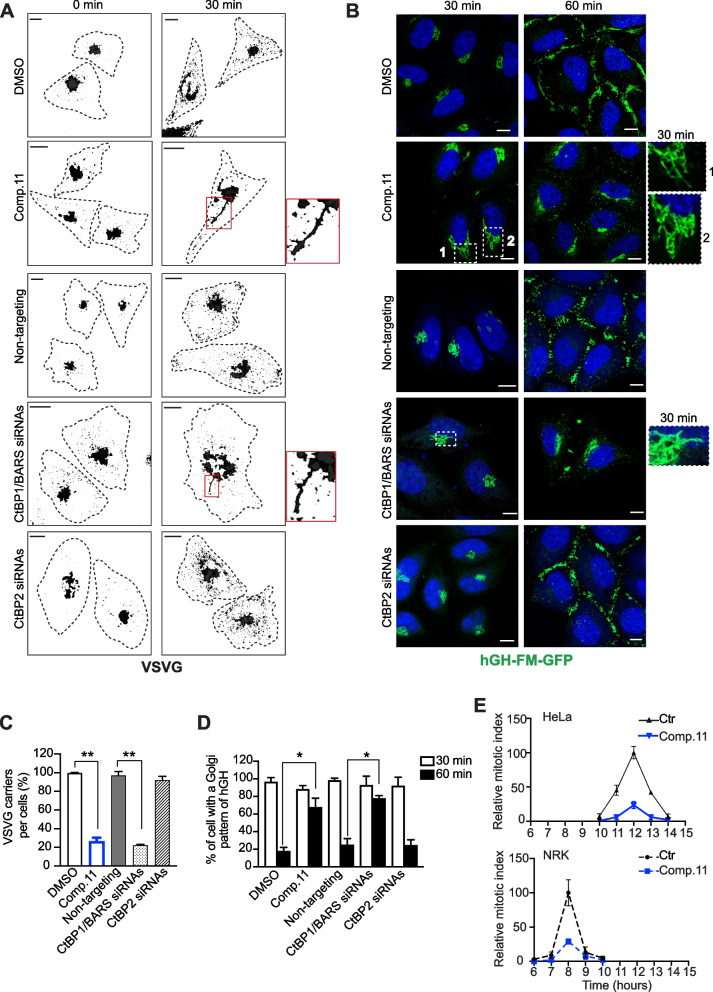
Comp.11 impairs the CtBP1/BARS-controlled protein transport and cell entry into mitosis. **A** Representative confocal microscopy images of A375MM cells infected with VSV and subjected to TGN-exit assay with 0.5% tannic acid. The cells were transfected with non-targeting or CtBP1/BARS siRNAs or CtBP2 siRNAs for 48 h, and subjected to VSV infection or treated with DMSO (vehicle control) or with Comp.11 (15 μM) 2 h at the 20 °C block during the TGN-exit assay (see Methods). The cells were fixed following the 20 °C (0 min) or 30 min after the shift to 32 °C, and processed for immunofluorescence with anti-VSVG (p5D4) antibody, to monitor formation of VSVG-containing carriers. Dotted lines show cell borders. Inserts, right: magnification of the tubular carrier precursors in the Golgi area. **B** Representative images of HeLa cells stably expressing hGH-FM–GFP and transfected with non-targeting or CtBP1/BARS siRNAs or CtBP2 siRNAs for 48 h or treated with DMSO (vehicle control) or Comp.11 (15 μM) for 2 h before subjection to a secretion assay (see Methods). Release of hGH-FM–GFP from ER was performed by the addition of DD-solubilizer at 37 °C for the indicated times. Insets, right: magnification of the tubular carrier precursors in the Golgi area. Scale bars, 10 μm. **C** Quantification of VSVG-positive carriers in **A**. **D** Quantification of hGH-FM–GFP in the Golgi area in B (see Methods). Data are means ± SD of three independent experiments. *P ≤ 0.05, **P ≤ 0.01 *versus* DMSO (vehicle control) or non-targeting (Student’s t-tests). **E** HeLa and NRK cells (as indicated) were arrested in S phase by using the double-thymidine block. Four hours after the S-phase block release, the cells were either treated with 15 μM Comp.11 or with DMSO (vehicle control) and fixed and processed for immunofluorescence at the indicated times after thymidine removal. Cells were labeled with Hoechst 33,342 to determine the mitotic indices up to 14 h after S phase release (see Methods)

To understand whether Comp.11 has an impact on these CtBP1/BARS-controlled cellular functions, we firstly investigated whether Comp.11 affects the export of the basolateral cargo VSVG from the *trans*-Golgi Network (TGN) to the plasma membrane (PM) using a well characterized transport assay which relies on the thermosensitive mutant protein ts045 from VSV [64]. Briefly, melanoma A375MM cells were infected with VSV and incubated at 40 °C to first accumulate the protein in the endoplasmic reticulum (ER) and then shifted to 20 °C, a temperature at which cargo proteins exit the ER and reach, but cannot exit, the TGN. The temperature was finally shifted to 32 °C, and the formation of VSVG-containing carriers from the TGN was visualized by immunofluorescence and quantified by preventing the fusion of these carriers with the PM with tannic acid [68], resulting in accumulation of carriers close to the cell surface. Comp.11 induced a strong reduction of the formation of the VSVG-positive post-Golgi carriers (Fig. 4A and C). Similar results were observed in CtBP1/BARS depleted cells; conversely neither CtBP2 knockdown nor MTOB, HIPP and PPγ treatments affect VSVG carrier formation (Fig. 4A, C and Supplementary Fig. 7). Of note, Comp.11-treated cells, as well as CtBP1/BARS depleted cells, showed several long (> 10 nm) tubular extensions that contained VSVG. These tubules represent carrier precursors that elongate out of the Golgi but are unable to undergo fission and detach to form mature transport carrier intermediates (Fig. 4A**)**. This fission-defect phenotype resembles that induced by depletion and inhibition of LPAAT δ, 14–3-3γ and other components of the CtBP1/BARS-fission machinery [35, 64, 68].

Along this line, we analyzed a soluble basolateral cargo, the stably expressed constitutively secreted GFP-tagged variant of the human growth hormone (hGH) that is retained in the ER and synchronously released in a temperature-independent fashion [83]. The treatment with Comp.11, as well as the depletion of CtBP1/BARS (but not of CtBP2) strongly inhibited export of hGH-FM–GFP from the Golgi to the PM with a similar fission-defect phenotype seen for VSVG cargo (Fig. 4B-D).

These findings indicate that Comp.11 after binding to CtBP1/BARS compromises the fission of basolateral-directed tubular carriers exiting the Golgi complex, and, in turn, blocks the transport of VSVG and hGH to the PM. Of note, Comp.11 treatments had no effect on CtBP1/BARS and CtBP2 protein levels (Supplementary Fig. 8A and B), as well as on cell viability, growth and morphology for the duration of the traffic-pulse experiments and longer.

Finally, we investigated the effects of Comp.11 in G2-blocked cells and monitored the effect of this treatment on mitotic entry, which depends tightly on Golgi fragmentation [61]. To this purpose we used HeLa and NRK cells, two well-known cell systems, to synchronize cells at G2/M boundary by the double-thymidine S-phase block. The cells, 4 h after thymidine washout, were incubated with Comp.11 and then fixed at various times to determine the mitotic index (see Methods). As shown in Fig. 4E there is a strong impairment (by 75%), with no delay, of entry into mitosis in both Comp.11-treated cell lines. These data indicate that Comp.11 inhibits the CtBP1/BARS-mediated cleavage of Golgi ribbon that occurs during the G2 phase of the cell cycle.

The conclusion that can be made from the above data is that Comp.11 interfering with the assembly of the CtBP1/BARS-fission machinery (unlike MTOB, HIPP and PPγ) compromises the cellular function of this protein complex, which results: *i)* in an impaired transport of basolateral cargoes to the PM, and, *ii)* in a block of mitotic Golgi fragmentation, a step that controls cell entry into mitosis.

#### Comp.11 inhibits cell proliferation by inducing G0/G1 phase cell cycle arrest in melanoma cells

**Fig. 5:**
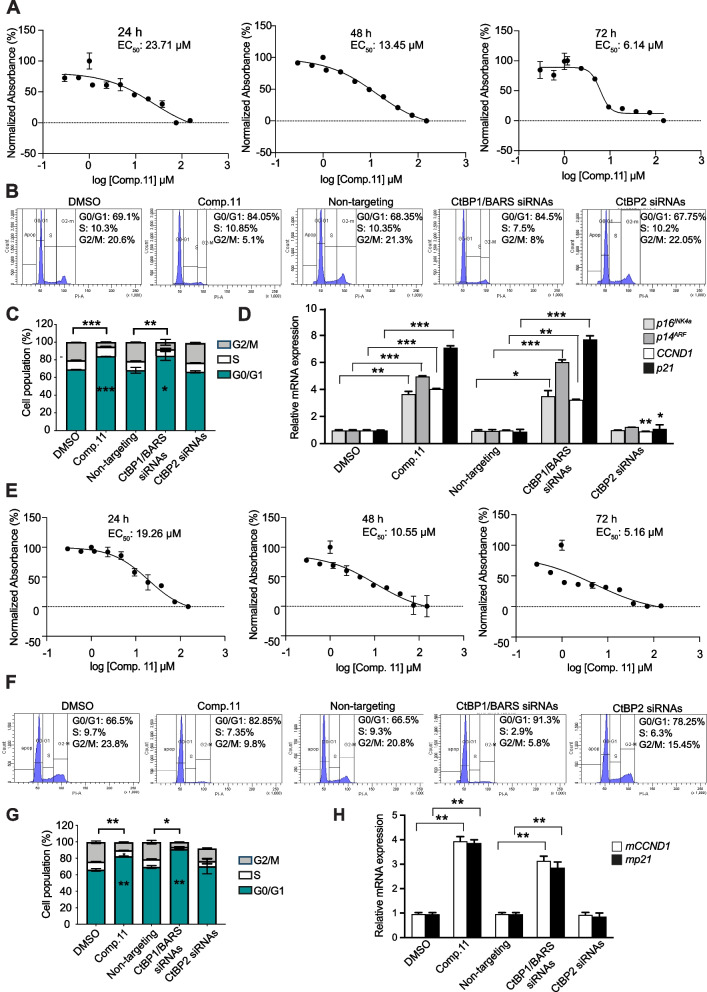
Comp.11 inhibits cell proliferation in melanoma cells. **A**, A375MM cells and **E**, B16F10 cells were treated with increasing concentrations of Comp.11 (from 0 to 150 μM) for 24 h, 48 h and 72 h and their viability was evaluated according to MTT assay. The graphs represent the dose–response of log10 concentrations of Comp.11 *versus* normalized optical intensity at 570 nm. EC_50_ values of Comp.11 were calculated and reported as indicated. **B** A375MM cells and **F** B16F10 cells treated for 24 h with DMSO (vehicle control) or Comp.11 (15 µM) or transfected for 48 h with non-targeting or CtBP1/BARS siRNAs or CtBP2 siRNAs (as indicated) were subjected to cell cycle analysis by flow cytometry using propidium iodide (PI) staining. **C** and **G**, Quantification of the FACS analysis reported in **B** and in **F**, respectively. Relative mRNA levels of *p16*^*INK4a*^, *p14*.^*ARF*^*, p21*, and *CCND1* in A375MM cells **D**, and of murine *p21* and *CCND1* (*mp21* and *mCCND1*, as indicated) in B16F10 cells **H**, measured by real time PCR after 24 h of treatment with DMSO (vehicle control) or Comp.11 (15 μM) or after 48 h of transfection with non-targeting or CtBP1/BARS siRNAs or CtBP2 siRNAs (as indicated). *GAPDH* is used as housekeeping gene. Data are means ± SD of three independent experiments. *P ≤ 0.05, **P ≤ 0.01, ***P ≤ 0.001 *versus* DMSO (vehicle control) or non-targeting (Student’s t-tests)

Having established the more pronounced expression levels of CtBP1/BARS in the two A375MM and B16F10 melanoma cell lines (Supplementary Fig. 1A), we investigated the cytotoxic effects of Comp.11 on these cells (and compared these effects with those of MTOB, HIPP and PPγ inhibitors; Fig. 5A-E, see also Supplementary Figs. 9, 10 and 11). The cells were treated with different concentrations of Comp.11 up to 150 µM for 24 h, 48 h and 72 h and the cell viability was measured by the MTT assay (Supplementary Fig. 9). As shown in Fig. 5A-E, the addition of Comp.11 results in a dose-dependent loss of viability in both cell lines, with EC_50_ upon 24 h treatment of 23.71 μM on A375MM and of 19.36 μM on B16F10 cells (see also Supplementary Fig. 9). PPγ was found more cytotoxic than Comp.11 (cellular EC_50:_12.68 μM vs 23.71 μM) and MTOB and HIPP (cellular EC_50:_13.47 mM and 123 μM) upon 24 h treatments on A375MM (Supplementary Fig. 10, see also Supplementary Fig. 11 for B16F10 cells). Accordingly, all additional in vitro experiments were performed with 15 μM Comp.11 treatment and with 5 μM PPγ, 5 mM MTOB and 50 μM HIPP treatments.

To further investigate whether Comp.11 inhibited cancer cell proliferation by inducing cell cycle arrest, we treated the two melanoma cell lines with this compound and examined the cell cycle distribution by flow cytometry (see Methods). Figure 5B, C, F and G indicate an increase in the mean percentage of cells in the G0/G1 phase of the cell cycle from 69.1% and 66.5% (vehicle alone) to 84.05% and 82.85% (Comp.11), respectively. G0/G1 phase cell cycle arrest was accompanied by a decrease in the percentage of cells in the G2/M (as found by thymidine cell-cycle synchronization assays, see Fig. 4E) and S phases (Fig. 5B, C and F-G). Similar results were observed in the cell cycle distribution of CtBP1/BARS-depleted but not in CtBP2-depleted cells (Fig. 5B, C and F-G; see also Supplementary Fig. 12A) indicating that Comp.11 inhibits cell proliferation through its specific interaction with CtBP1/BARS. Indeed, Comp.11 does not bind CtBP2 as validated via ITC experiments (Supplementary Fig. 13).

The transcriptional activity of CtBP1/BARS controls several genes involved in cell proliferation and tumor growth, including *p16*^*INK4a*^, *p14*^*ARF*^*, Ciclin D1* and *p21* [29, 31].

To define the mechanism of the anti-proliferative effects of Comp.11, we explored whether this compound does interferes with the expression of the above cell cycle-related genes by real-time PCR. As shown in Fig. 5D, *p16*^*INK4a*^, *p14*^*ARF*^*, Ciclin D1* and *p21* exhibited a significant increase in their expression levels upon Comp.11 treatment, in A375MM cells. Similar results were obtained in CtBP1/BARS-depleted cells but not in CtBP2-depleted cells or in MTOB-, HIPP- and PPγ-treated cells (as a demonstration of on-target specificity of Comp.11 action on CtBP1/BARS functions) (Fig. 5D; see also Supplementary Fig. 14A). In parallel, upon Comp.11 treatment, increased expression levels of *Ciclin D1* and *p21* were evaluated in murine B16F10 cells (Fig. 5H; see also Supplementary Fig. 14B), which express a level of CtBP1/BARS protein comparable to that in A375MM cells (Supplementary Fig. 1A).

Altogether, these results demonstrate that binding of Comp.11 to CtBP1/BARS abrogates its transcriptional activity and inhibits cell proliferation by inducing cell cycle arrest in the G0/G1 and G2/M phases.

#### Comp.11 induces apoptosis in melanoma cells

**Fig. 6:**
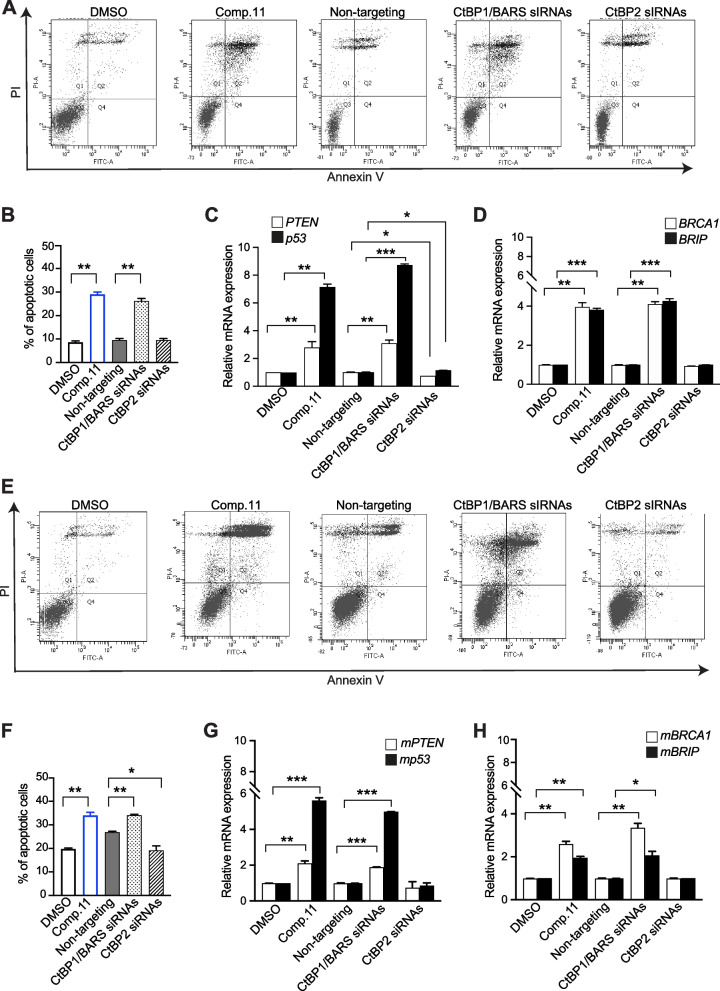
Comp.11 induces apoptosis in melanoma cells. **A**, A375MM cells and **E**, B16F10 cells were stained with Annexin V/PI, and analyzed by FACS after 24 h of treatment with DMSO (vehicle control) or Comp.11 (15 μM) or after 48 h of transfection with non-targeting or CtBP1/BARS siRNAs or CtBP2 siRNAs (as indicated). **B** and **F** Quantification for Annexin V-positive apoptotic cells in A and E, respectively (see Methods). Relative mRNA levels of the reported genes measured by real time PCR in A375MM cells **C-D**, and in B16F10 cells **G-H,** after 24 h of treatment with DMSO (vehicle control) or Comp.11 (15 μM) or after 48 h of transfection with non-targeting or CtBP1/BARS siRNAs or CtBP2 siRNAs (as indicated). *GAPDH* is used as housekeeping gene. Data are means ± SD of three independent experiments. *P ≤ 0.05, **P ≤ 0.01, ***P ≤ 0.001 *versus* DMSO (vehicle control) or non-targeting (Student’s t-tests)

Flow cytometry analysis by annexin V/PI staining was performed in order to investigate the induction of apoptosis in melanoma cells by Comp.11. The percentages of viable, early apoptotic, late apoptotic, and necrotic cells after 24 h of treatment with 15 μM Comp.11 are shown in Fig. 6A. Significant differences were observed between control and treated cells: 7.6% of total percentage of apoptotic cells in vehicle-treated A375MM cells *versus* 28.7% in Comp.11-treated cells. Similar effects of Comp.11 treatments were observed in CtBP1/BARS-depleted cells but not in CtBP2-depleted cells (Fig. 6A and B and Supplementary Fig. 12B).

To understand the mechanism of apoptosis induced by Comp.11, we examined the expression levels of *p53* and *PTEN* tumor suppressors genes, which are both reported to inhibit cell cycle progression and promote apoptosis under the transcriptional control of CtBP1/BARS [30]. The treatment of A375MM with Comp.11, or with CtBP1/BARS siRNAs, resulted in a marked increase in PTEN (sevenfold) and in p53 (threefold) mRNA levels (Fig. 6C) compared with that in vehicle- or non-targeting-treated cells. A negligible effect in p53 mRNA level was observed in CtBP2 depleted cells (Fig. 6C) and opposite effects were seen in MTOB-, HIPP- and PPγ-treated cells (Supplementary Fig. S15A).

CtBP1/BARS is also involved in genome instability through its transcriptional regulation of *BRCA1* gene*,* which dampens DNA-damage repair in melanoma. Indeed, in melanoma patients, the increased expression level of CtBP1/BARS correlates with decreased expression and function of *BRCA1*, and this contribute to genome instability and melanoma initiation [29]. We found that upon CtBP1/BARS depletion the expression levels of *BRCA1* increased (Fig. 6D). In light of this finding, we investigated the possibility that by blocking CtBP1/BARS functions with Comp.11, we could prevent melanoma progression by increasing the *BRCA1/BRIP1*-controlled DNA-Damage Response Pathway [95]. As shown in Fig. 6D, both the mRNA levels of *BRCA1* and *BRIP1* increased fourfold in Comp.11- and in CtBP1/BARS siRNAs-treated cells compared to the vehicle- or non-targeting-treated A375MM cells. No effects were observed in MTOB- and HIPP-treated cells and a negligible opposite effect was seen in PPγ-treated cells (Supplementary Fig. S15B). Similar data were observed in murine B16F10 melanoma cells upon the above treatments (Fig. 6E-H; see also Supplementary Fig. S15C and D).

In conclusion, these observations indicate that Comp.11 treatment is able to reverse the CtBP1/BARS-mediated transcriptional repression of *PTEN, p53, BRCA1* and *BRIP1* genes, activating apoptosis and reducing melanoma initiation.

#### Comp.11 impairs motility and invasion of melanoma cells by affecting the CtBP1/BARS- mediated transcription of EMT-related genes

A key pro-oncogenic function in which CtBP1/BARS has been implicated is the ability to promote cancer cell migration and invasion [31, 96], which is related to CtBP1/BARS role in induction of EMT and metastasis [32]. Indeed, CtBP1/BARS expression and activity has been found to be upregulated in metastatic cancer types [97] where this protein repressed epithelial marker genes such as *E-cadherin (CDH1)*, *plakoglobin*, *desmoglein-2*, *occludin* [44, 45, 58], *beta-catenin* [98] and increased the expression of mesenchymal marker genes including *vimentin*, *N-cadherin*, *Snail* [99] and *versican* [VCAN [96]].

**Fig. 7:**
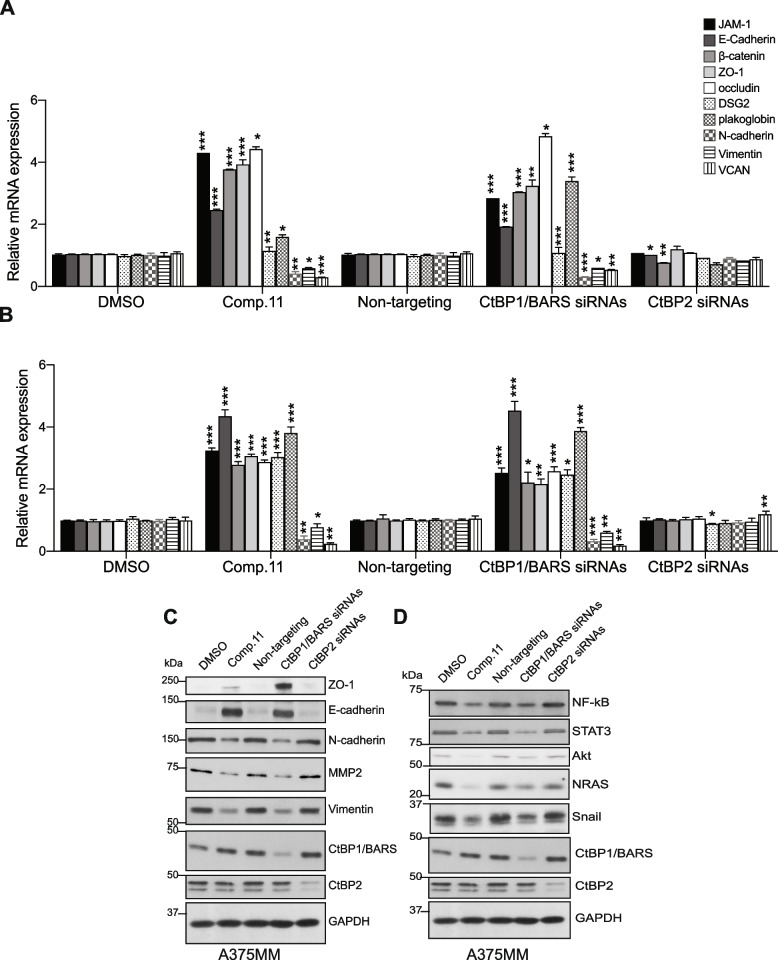
Comp.11 affects the CtBP1/BARS-mediated transcription of EMT-related genes. Relative mRNA levels of epithelial markers (*E-cadherin*, *plakoglobin*, *β-cathenin*, *Desmoglein 2*, *Occludin*, *JAM-1*, *ZO1*) and mesenchymal markers (*N-cadherin*, *Vimentin* and *Versican*) in A375MM cells **A**, and in B16F10 cells **B**, measured by real time PCR after 24 h of treatment with DMSO (vehicle control) or Comp.11 (15 μM) or after 48 h of transfection with non-targeting or CtBP1/BARS siRNAs or CtBP2 siRNAs (as indicated). *GAPDH* is used as housekeeping gene. Data are means ± SD of three independent experiments performed in triplicate. *P ≤ 0.05, **P ≤ 0.01, ***P ≤ 0.001 *versus* Ctr or non-targeting (Student’s t-tests). **C** and **D** Representative Western blotting of the indicated proteins in A375MM cells treated as in A. GAPDH is shown for the internal protein levels and molecular weight standards (kDa) are indicated on the left of each panel. Data are representative of three independent experiments

We have thus evaluated whether Comp.11 can affect melanoma cell migration and invasion by reverting the transcription of these CtBP1/BARS-controlled EMT-related genes. Total mRNA extraction and real-time PCR determination were applied on A375MM and B16F10 cells, both recognized to possess strong migratory and invasive abilities. As shown in Fig. 7A and B, the addition of Comp.11 increased by several folds the epithelial *JAM-1*, *E-cadherin*, *beta-catenin*, *Zona occludens 1 (ZO-1)*, *occludin, desmoglein-2 (DSG2)* and *plakoglobin* genes, and conversely impaired the induction of mesenchymal *N-cadherin*, *vimentin* and *VCAN* genes compared to vehicle-treated cells. Similar results were observed in both A375MM and B16F10 cells, upon depletion of CtBP1/BARS but not of CtBP2 (see also Fig. 7C and D). Only negligible effects were observed in *DSG2* and *plakoglobin* genes upon HIPP treatment and in *plakoglobin* gene upon PPγ treatment (Supplementary Fig. S16). Western blot analysis of cell lysates treated with Comp.11 (or with CtBP1/BARS siRNAs) showed a reduction of the well-known signaling proteins associated with melanoma development and progression: NRAS and Akt [identified as CtBP repression target, [45]], and NF-kB and STAT3 [100] (Fig. 7C and D).

Altogether, these data indicate that Comp.11 is capable of reversing the CtBP1/BARS-mediated transcriptional activity and this could be explained by alteration in the oligomerization state of CtBP1/BARS bound to Comp.11, which, in turn, is unable to form the active CtBP1/BARS transcriptional complex [32].

**Fig. 8:**
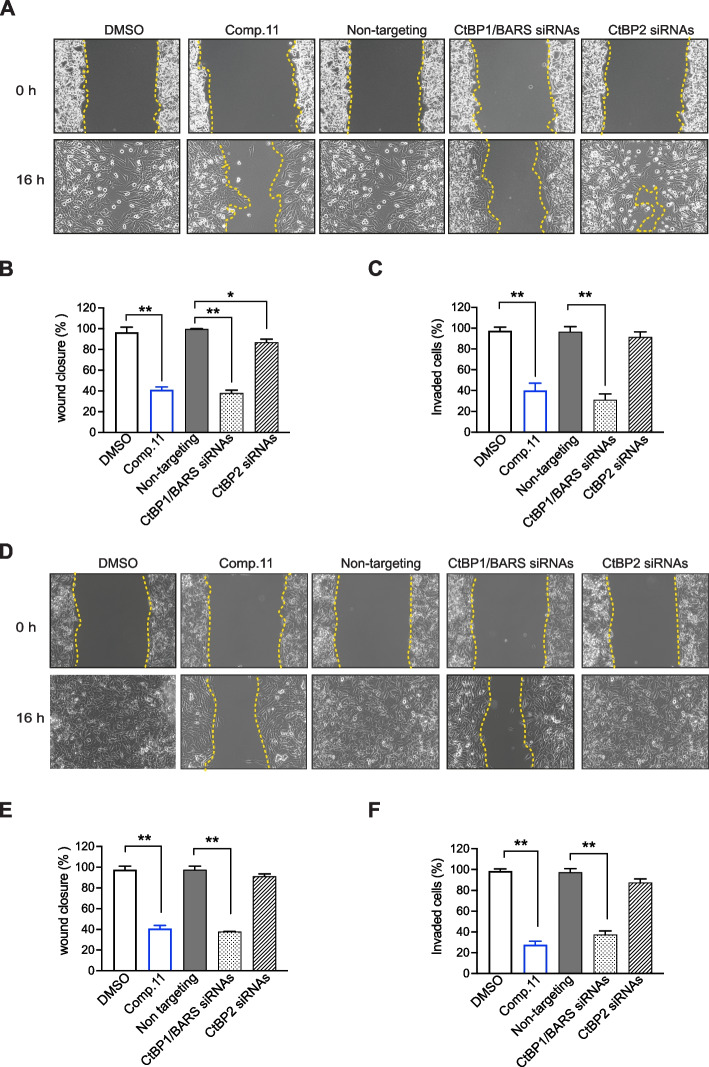
Comp.11 impairs melanoma cell migration and invasion. Representative images of wound closure assays (at the indicated times; see Methods) in A375MM cells **A**, and in B16F10 cells **D**, after 24 h of treatment with DMSO (vehicle control) or Comp.11 (15 μM) or after 48 h of transfection with non-targeting or CtBP1/BARS siRNAs or CtBP2 siRNAs (as indicated). **B** and **E**. Quantification of the extent of wound closure calculated by analyzing the scratched area covered by the A375MM cells (**B**) or B16F10 cells (**E**) after 16 h using ImageJ software. **C** and **F**. Quantification of Matrigel invasion assays of A375MM and B16F10 cell lines, respectively, treated as in **A** and **B**. Invasion was quantified by counting cells in ten random fields and data are presented as mean number of cells/field. Data are mean ± SD of three independent experiments. *P ≤ 0.05, **P ≤ 0.01 *versus* DMSO (vehicle control) or non-targeting (Student’s t-tests)

Based on the above results, we analyzed the effects of these gene alteration upon Comp.11 treatment in cell migration and invasion by wound healing and transwell matrigel invasion assays. Both A375MM and B16F10 cells, exposed to 15 μM Comp.11 or to CtBP1/BARS depletion (see Methods), showed a significantly reduced cell migration ability compared with the controls (Fig. 8A-D and Supplementary Fig. 17). Quantification performed at 16 h after scratching showed that the wound closure was reduced by 60% compared to the controls (Fig. 8B-E). A minor effect in A375MM cell migration was observed in CtBP2 depleted cells (Fig. 8B). The transwell invasion assays also showed that Comp.11 or CtBP1/BARS siRNAs treatment inhibited cell invasion (Fig. 8C-F and Supplementary Fig. 10), indicating an inhibitory role of CtBP1/BARS bound to Comp.11 on melanoma cell invasion. Of note, no effects on melanoma cell invasion ability were seen upon treatments with MTOB, HIPP and PPγ (Supplementary Fig. S18A and B).

Overall, our data indicate that Comp.11 directly binds to CtBP1/BARS and thus inhibits the transcriptional complex function of CtBP1/BARS shifting gene expression patterns from mesenchymal to a more epithelial phenotype, leading to an impairment of melanoma cell migration and invasion.

#### *Comp.11 triggers reduction in colony formation and *in vivo* primary tumor growth*

**Fig. 9:**
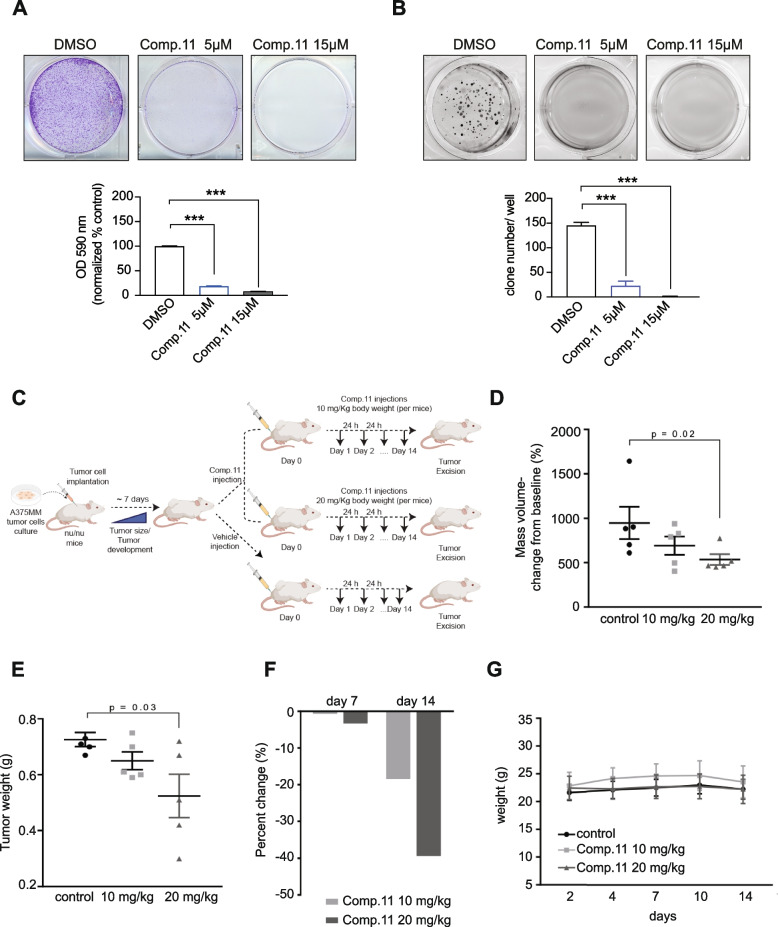
Comp.11 exhibited antitumor activity in colony formation and in vivo tumor growth. **A** Top: Representative images of crystal violet staining of A375MM cells in the colony formation assays after 7 days of treatment with DMSO (vehicle control) or with Comp.11 (5 or 15 μM) (see Methods). Bottom: Quantification of the Stained colonies dissolved in 33% acetic acid by measuring the absorbance at 590 nm and normalized *versus* DMSO (vehicle control). **B**. Top: Representative images of soft agar colony formation assay. A375MM cells were grown in agar-media suspension for 21 days in presence of DMSO (vehicle control) or of Comp.11 (5 or 15 μM). Plates were incubated overnight with Nitroblue Tetrazolium Chloride dye to visualize colonies on a gel imager. Bottom: Quantification of clone number/ well of A375MM cells after 21 days of treatments. Data are mean ± SD of two independent experiments performed in triplicate ***P ≤ 0.001 *versus* DMSO (control vehicle) (Student’s t-tests). **C** Schematic diagram of the experimental design and steps for the in vivo studies. A375MM cells (2.5 $\times$ 10^6^ cells) were s.c. injected into the flank of female CD1 nude mice as described in Methods. When tumors were established mice (*n* = 5, each) were randomly divided into three experimental groups (*n* = 3) to receive Comp.11 at the dosages of 10 mg/kg (daily, for 2 weeks), 20 mg/kg (three times/week for 2 weeks) or its vehicle intraperitoneally administrated for 14 days. **D** Fold change in tumor volume (% of means ± SEM) compared with the baseline [determined by caliper and calculated as (length $\times$ width2)/2] of A375MM tumors after two weeks of Comp.11 treatments. Statistically significant results are reported (*P* < 0.02) for Comp.11 20 mg/kg treatment. **E** At the end of the experiment (day 14), the tumors were dissected and tumor weight were determined. Statistically significant results are reported (*P* < 0.03) for Comp.11 20 mg/kg treatment. **F** Percent change in tumor volume average from each group of A375MM model at day 7 and day 14 were compared and presented as percentages of vehicle. **G,** Mice body weight as surrogate indicator of toxicity for *in vivo* experiment reported in D. Body weight was measured three times/week. *P* values in **D** and **E** were calculated using one way ANOVA correct by original FDR method of Benjamini and Hochberg

We decided to explore further the anti-tumor effects of Comp.11 in anchorage-dependent and anchorage-independent (in soft agar) colony formation assays. The A375MM cell line was particularly sensitive, and exhibited a significant decrease in colony-forming potential (see Methods) in response to treatment with Comp.11. As shown in Fig. 9A and B, the inhibition of colony formation was dose-dependent, with higher doses of 15 μM (consistent with the dose that induced apoptosis; see also Fig. 6). Of note, unlike Comp.11, the other CtBPs inhibitors: HIPP and PPγ had minimal effects on colony formation (Supplementary Fig. S18C), indicating that, under these conditions, Comp.11 is the most potent and selective inhibitor to interfere with melanoma cell growth.

In order to confirm in vivo the antitumor effect observed in vitro*,* we evaluated the effect of Comp.11 in a A375MM xenograft model (Fig. 9C-G). Specifically, A375MM cells were injected in the right flank of fifteen mice, when the tumors became palpable, the mice were randomly assigned to three experimental groups (*n* = 3) to receive Comp.11 (10 mg/kg daily for 2 weeks or 20 mg/kg three times/week for 2 weeks) or its vehicle as schematized in Fig. 9C (see Methods). Comp.11 20 mg/kg produced a statistically significant tumor growth inhibition (*p* = 0.02) compared with control group, evaluated as % of means of the fold change of the tumour volume for each group (Fig. 9D) after 2 weeks of treatment, compared with the baseline. The waterfall plot of the fold-change in tumor volume compared with the baseline for each mouse is reported in Supplementary Fig. 19A.

A statistically significant tumor inhibition (*p* = 0.03) of Comp.11 20 mg/kg treatment was confirmed by evaluating the weight of tumors collected at the end of study (day 14) as shown in Fig. 9E. Moreover, by calculating the percent change in tumor volume from the time of initial treatment (day 0) to day 7 or day 14 (end of the treatment) of the study, Comp.11 20 mg/kg treatment reduced the tumor burden by 3.3% and 39.39%, respectively, in spite of the other treated group (Comp.11 10 mg/kg) that reduce tumor burden of 0.68% and 18.4% respectively (Fig. 9F). The treatment was well tolerated by xenografted mice, as shown by the maintenance of body weight (Fig. 9G) and by the absence of other signs of acute or delayed toxicity.

Next, we confirmed, a statistically significant inhibition of tumor volume after Comp.11 20 mg/kg treatment respect to vehicle (*P* = 0.04) by the High Frequency Ultrasound (HFUS) (Supplementary Fig. 19B).

Altogether, these data suggest that Comp.11, with the best results at the dosage of 20 mg/kg, once bound to CtBP1/BARS, inhibits growth of melanoma tumor in xenograft models, indicating its potential effective antitumor activity.

## Discussion

Based on the increasing evidence on the CtBPs properties for oncogenesis, tumor growth and progression/metastasis, this protein family represents a potential therapeutic target in neoplastic disease [29–31, 81, 101, 102].

We report here the identification of N-(3,4-dichlorophenyl)-4-{[(4 nitrophenyl)carbamoyl] amino} benzenesulfonamide (for brevity Comp.11) as a new, potent and selective inhibitor of CtBP1/BARS. This molecule by interacting with the NADH-binding Rossmann fold region of CtBP1/BARS, affects the oligomerization state of the protein (unlike the other CtBPs inhibitors: MTOB, PPγ and HIPP derivates), which in turn, inhibits both the CtBP1/BARS cellular functions: *i)* membrane fission, with block of mitotic entry and cellular secretion; and *ii)* transcriptional pro-tumoral effects with hampered survival, EMT, migration/invasion. This combination of effects impairs melanoma tumor growth in mouse models providing substantial evidence that pharmacological targeting of CtBP1/BARS may be a feasible strategy for future development of novel melanoma treatments.

PPγ was found more cytotoxic than Comp.11, MTOB and HIPP. Moreover, PPγ, MTOB and HIPP, when used below their EC_50_, proved ineffective in the inhibition of 14–3-3γ binding and cellular secretion as well as in the inhibition of E1A binding and on transcriptional pro-tumoral effects with invasion, and colony-forming capabilities.

These findings underscore the distinct capability of Comp.11 to finely regulate CtBP1/BARS with higher selectivity and potency on melanoma cells than the other reported inhibitors of CtBPs.

Furthermore, unlike Comp.11, inhibitors MTOB, PPγ, and HIPP have been shown to bind indiscriminately to both CtBP1/BARS and CtBP2. Despite their evaluation across various cancer models [32, 103, 104] there is currently no reported application of these compounds in melanoma. Taken together, these data support a distinct and innovative mechanism of Comp.11 in inhibiting melanoma growth through its selective binding to CtBP1/BARS. Overall, the present study highlights the progress made by our group on the theory and rationale for the identification and validation of a selective and potent CtBP1/BARS inhibitor but also provides an important tool to define the functional role of CtBP1/BARS in tumor biology.

The CtBPs differ from all the other eukaryotic NAD(H)-dependent D-2-hydroxyacid dehydrogenases in that they contain a unique tryptophan residue, W307 in CtBP1/BARS (W318/324 in CtBP1-L/CtBP2) within their large hydrophilic water-filled catalytic site [32, 105]. This W307 residue has been shown as a “keystone” residue that links NAD(H) binding to CtBP dimerization/oligomerization, transcriptional regulation and induction of cell migration [91, 105].

Notably, crystal structure of CtBPs and MTOB revealed that the indolyl ring of W307 is crucial for MTOB orientation via its thioether group in the CtBP catalytic site while the other extremity of MTOB is oriented towards the phosphate group of NADH [71]. The substitution of thioether group in MTOB with phenyl ring increased the binding with W307, with an affinity greater than 1000-fold over that MTOB, by the π-π stacking between the phenyl ring and the W307 indolyl ring. This led to the identification of a more potent competitive class of CtBP inhibitors: HIPP and derivatives [72, 85], which further showed promising preclinical anticancer activity [72, 106, 107]. Subsequently, the substitution with chlorine group at the para- and meta-position on the HIPP phenyl ring enhanced significantly the inhibition of CtBP transcriptional activity, and in turn, the cytotoxicity effects of these chlorinate HIPP derivatives on tumor cells [72].

In this study, the chlorine group in the HIPP phenyl ring is replaced with a nitrobenzene functional group in Comp.11 molecule (Fig. 1B). The effect of this nitro-substitution in para-position enhanced binding efficiency of Comp.11 (Kd = 0.66 μM) over the HIPP [Kd = 2.77 μM; [85]] to CtBP1/BARS. Here, in addition to the π-π stacking between the phenyl ring and the W307 indolyl ring, a further stabilization of Comp.11-CtBP1/BARS complex would be likely explained by an H-bond between the nitro group and the R86, which probably coordinates the orientation of the Comp.11 molecule within the pocket (Fig. 2B). The substitution of this arginine with alanine completely abolishes the Comp.11-CtBP1/BARS complex formation (Fig. 2F).

At the other extremity, Comp.11 is larger than MTOB and comprises a benzenesulfonamide moiety, which is accommodated by extended coordination via H-bond with D23 and a stack with the phenol ring of Y25 (Fig. 2B) in a region critical for CtBP1/BARS oligomer formation [91]. The binding of Comp.11 in this region that accommodates the “keystone” residue W307 directly locks CtBP1/BARS in an “open oligomeric conformation” (Fig. 3A) that hinders the release of NADH (Fig. 2A). Consequently, CtBP1/BARS is trapped in an inactive abortive Comp.11-CtBP1/BARS-NADH ternary complex that prevents its binding to both transcription and membrane fission partners (Fig. 3B-F).

Functionally, Comp.11 compromises the regulatory network of genes associated with multiple “cancer hallmarks” and malignant transformation under the specific control of CtBP1/BARS in melanoma cells. Specifically, upon binding to CtBP1/BARS, Comp.11: 1. controls the expression of cell proliferation genes such as *p16INK4a, p21, p14ARF* and *CCND1* resulting in the induction of cell cycle arrest (Fig. 5); 2. controls the expression of cell survival and apoptotic genes such as *p53, PTEN, BRCA1, BRIP1* with, consequently, the induction of apoptosis (Fig. 6); 3. leads to an inhibition in the mobile and invasive mesenchymal phenotype by shifting gene expression patterns towards more epithelial patterns (Fig. 7– 8) resulting in reduced tumor cell aggressiveness; 4. inhibits the growth of primary tumor in animal model (Fig. 9). Future studies in metastatic animal models will help to evaluate the effect of Comp.11 in hindering metastatic spread.

Changes in mRNA levels of the above panel of genes are similar between Comp.11 treatment and CtBP1/BARS knockdown (Fig. 5– 8). This may be due to the ability of Comp.11 to sequester CtBP1/BARS in an inactive abortive transcriptional complex unable to occupy the promoter regions, thereby influencing cellule processes under the transcriptional control of CtBP1/BARS. Moreover, as a demonstration of on-target specificity of Comp.11 action, depletion of CtBP2 does not affect the mRNA levels of these analyzed CtBP1/BARS-controlled genes (Fig. 5– 8).

Over the last decades, different classes of sulfonamides and their analogs have been identified as promising candidates to develop novel class of antitumor agents [108]. These molecules, by targeting the enzymatic activity of carbonic anhydrases (CAs), inhibited breast cancer cell growth and migration in vitro, and some of them suppressed breast tumor growth and metastases in mouse models [109–111].

Moreover, a class of arylureido-benzenesulfonamides is under clinical investigation for the treatment of solid malignancies. A highly selective inhibitor of CA type IX (CAIX), namely SLC-0111, is currently: *i)* in phase Ib / II clinical trial for the treatment of advanced and metastatic solid tumors, and; *ii)* is being studied as an adjuvant to avoid therapeutic failure due to drug resistance development and to improve the efficacy of anticancer pharmacological therapies [108, 112–114]. Specifically, SLC-0111 inhibits the enzymatic activity of CAIX, an enzyme that, catalyzing the conversion of carbon dioxide into carbonic acid, controls the intracellular and extracellular pH. Hypoxia condition induces the expression of CAIX, which, in turn, contributes to the acidification of the extracellular milieu and promotes the acquisition of tumor malignancy [112, 115]. Treatment with 100 µM of SLC-0111 for 72 h affects the viability of the high-malignant acid cancer subpopulation particularly addicted to CAIX activity, *i.e.,* the cells growing under specific conditions of acidosis within the tumor mass of the breast, melanoma and colorectal tumors.

In summary, here we provide insight into the future development and use of a molecule N-(3,4-dichlorophenyl)-4-{[(4 nitrophenyl)carbamoyl] amino} benzenesulfonamide (Comp.11), which, despite comprising an ureido-substituted benzenesulfonamide functional group, is able to specifically target the pro-tumoral cell functions of the CtBP1/BARS protein at lower concentration (15 µM) and in shorter times (24 h) than those effective for SLC-0111. The resulting functional effect is the suppression of tumor growth, cell migration and invasion of melanoma cells grown under normoxic conditions. This selectivity towards CtBP1/BARS could be due to the other end of the Comp.11 molecule that specifically binds the catalytic pocket of CtBP1/BARS near the NADH molecule and containing the W307 unique for the CtBP proteins.

Furthermore, our results strongly support the anti-cancer properties of Comp.11. Nevertheless, whether Comp.11 could have a double anti-cancer effect by also inhibiting, via the benzenesulfonamide group, the viability of the subpopulation of melanoma cells grown under acid conditions, will be of particular interest for future studies. 

### Supplementary Information

**Additional file 1: Supplementary Fig. 1.** **A**. Representative Western blot analysis of CtBP1/BARS protein expression in HeLa, A375MM, B16F10, MDA-MB231 and PC3 cancer cells *versus* the epithelial normal PNT2 cells. β-actin is used as a loading control. Molecular weight standards (kDa) are indicated on the left of each panel. **B**. Representative confocal microscopy images of B16F10 cells treated with DMSO (vehicle control) or with Comp.11 (15 μM) for 2 h at 37°C. Cells were fixed and labeled with a polyclonal anti-CtBP1/BARS antibody (endogenous CtBP1/BARS; green) and with a monoclonal anti-GM130 antibody (used as a cis-Golgi marker; grey). Insets right: Magnification of Golgi area. Scale bars, 10 μm. Bottom; quantification of cells with a nuclear CtBP1/BARS pattern. Data are means ± SD of three independent experiments. **P ≤ 0.01 *versus* the vehicle DMSO (Student’s t-tests).

**Additional file 2: Supplementary Fig. 2.** Representative confocal microscopy images of HeLa cells **A**, and MCF7 cells **B**, treated with DMSO (vehicle control) or with Comp.11 (15 μM) for 2 h at 37°C. Cells were fixed and stained for endogenous CtBP1/BARS (green), TGN46 (red; used as a TGN-Golgi marker) and GM130 (grey; used as a cis-Golgi marker). Insets: Magnification of Golgi area. Scale bars, 10 μm. Bottom; quantification of cells with a nuclear CtBP1/BARS pattern. Data are means ± SD of three independent experiments. *P ≤ 0.05, **P ≤ 0.01 versus DMSO (vehicle control) (Student’s t-tests).

**Additional file 3: Supplementary Fig. 3.** Top: ITC traces for the titration of the protein CtBP1/BARS mutants (as indicated) with the Comp.11 as ligand. Bottom: Integrated ITC data (black squares) as a function ligand/protein concentration ratio. The solid red line is the best fit of experimental data using the independent site binding model.

**Additional file 4: Supplementary Fig. 4. A**. Representation of the predicted overlap between Comp.11 (in red) and the second monomer of CtBP1/BARS (orange chain) as would happen in the dimerization process. In yellow the residues along the second chain that collide with Comp.11. NADH is reported as well (in blue) and the first monomer of CtBP1/BARS is in light blue. **B**. Other representation of the incompatibility between Comp.11 (mashed red surface) and the second monomer of CtBP1/BARS (yellow solid surface).

**Additional file 5: Supplementary Fig. 5**. Evaluation of transfection efficiencies of post-nuclear supernatants used for the LPAAT assays in Fig. 3F. **A**. Representative Western blotting with anti-Flag and anti-GAPDH antibodies (as indicated) of post-nuclear supernatants from A375MM cells transfected for 48 h with an empty Flag-vector (Empty vector) or with LPAATδ–Flag (LPAATδ) and then incubated with 15 μM of Comp.11 (+) or with DMSO (-) for 30 min at 25°C. **B**. Representative Western blotting with anti-LPAATδ, anti-CtBP1/BARS and anti-GAPDH antibodies (as indicated) of post-nuclear supernatants from A375MM cells transfected for 72 h with non-targeting or CtBP1/BARS siRNAs or LPAATδ siRNAs. Molecular weight standards (kDa) are indicated on the left of each panel.

**Additional file 6: Supplementary Fig. 6. A**. Representative GST pull-down of GST-E1A recombinant protein for His-CtBP1/BARS. His-CtBP1/BARS protein was first incubated with DMSO (vehicle control) or with 5 mM MTOB or 50 µM HIPP or 5 µM PPγ or 15 µM Comp.11 (1 h, 4°C in a wheel), and then incubated with GST-E1A (2 h, 4°C in a wheel; see Methods). The bound proteins to the glutathione Sepharose beads were eluted and analyzed by western blotting with anti-His monoclonal antibody (top), with pulled-down proteins revealed by Ponceau-S staining (bottom). **B**. Representative GST pull-down of GST-14-3-3γ recombinant protein for His-CtBP1/BARS. His-CtBP1/BARS protein was pre-incubated with DMSO (vehicle control) or with 5 mM MTOB or 50 µM HIPP or 5 µM PPγ or 15 μM Comp.11 (1 h, 4°C in a wheel), and then incubated with GST-14-3-3γ (2 h, 4°C in a wheel; see Methods). The bound proteins to the glutathione Sepharose beads were eluted and analyzed by western blotting with anti-His monoclonal antibody (bottom), with pulled-down proteins revealed by Ponceau-S staining (bottom). Data are representative of three independent experiments.

**Additional file 7: Supplementary Fig. 7**. Representative confocal microscopy images of A375MM cells infected with VSV and subjected to TGN-exit assay with 0.5% tannic acid. The cells were treated with DMSO (vehicle control) or with 5 mM MTOB or 50 µM HIPP or 5 µM PPγ or 15 μM Comp.11 for 2 h at the 20°C block during the TGN-exit assay (see Methods). The cells were fixed following the 20°C (0 min) or 30 min after the shift to 32°C, and processed for immunofluorescence with anti-VSVG (p5D4) antibody, to monitor formation of VSVG-containing carriers. Dotted lines show cell borders. Scale bars, 10 μm. Quantification of VSVG-positive carriers (right). Data are means ± SD of three independent experiments. These data are not statistically significant (Student’s t-tests).

**Additional file 8: Supplementary Fig. 8. **Analysis of CtBP1/BARS and CtBP2 depletion in A375MM cells subjected to the VSV-traffic pulse **A**, and in HeLa cells stably transfected with hGH-FM–GFP and subjected to a secretion assay **B**, as reported in Fig. 4. Representative Western blotting with anti-CtBP1/BARS, anti-CtBP2 and anti-GAPDH antibodies (as indicated). Molecular weight standards (kDa) are indicated on the left of each panel.

**Additional file 9: Supplementary Fig. 9**. Comp.11 inhibits cell proliferation in melanoma cell lines. **A**, A375MM cells and **B**, B16F10 cells were treated with increasing concentrations of Comp.11 (from 0 to 150 µM) for 24 h, 48 h and 72 h and their viability was evaluated according to MTT assay (as reported in Fig. 5A and 5E). Absorbance was detected at 570 nm with a microplate reader. Data are expressed in MTT Reading and are means ± SD of three independent experiments performed in duplicate. *P ≤ 0.05, **P ≤ 0.01, ***P ≤ 0.001 *versus* Ctr (Student’s t-tests).

**Additional file 10: Supplementary Fig. 10**. A375MM cells were treated with increasing concentrations of MTOB (from 0 to 20 mM) or HIPP (from 0 to 150 µM) or PPγ (from 0 to 150 µM) for 24 h and 48 h and their viability was evaluated according to MTT assay. The graphs represent the dose-response of log10 concentrations of MTOB, HIPP or PPγ (as indicated) *versus* normalized optical intensity at 570 nm. EC50 values of MTOB, HIPP or PPγ were calculated and reported as indicated.

**Additional file 11:** **Supplementary Fig. 11**. B16F10 cells were treated with increasing concentrations of MTOB (from 0 to 20 mM) or HIPP (from 0 to 150 µM) or PPγ (from 0 to 150 µM) for 24 h and 48 h and their viability was evaluated according to MTT assay. The graphs represent the dose-response of log10 concentrations of MTOB, HIPP or PPγ (as indicated) *versus* normalized optical intensity at 570 nm. EC50 values of MTOB, HIPP or PPγ were calculated and reported as indicated.

**Additional file 12:** **Supplementary Fig. 12**. Analysis of CtBP1/BARS and CtBP2 depletion in A375MM and B16F10 melanoma cells subjected to the cell cycle analysis **(A)** and to the apoptosis analysis **(B)** as reported in Fig. 5 and Fig. 6. Representative Western blotting with anti-CtBP1/BARS, anti-CtBP2 and anti-GAPDH antibodies (as indicated) of A375MM cells and B16F10 cells treated for 24 h with DMSO (vehicle control) or Comp.11 (15 μM) or transfected for 48 h with non-targeting or with CtBP1/BARS siRNAs or CtBP2 siRNAs. Molecular weight standards (kDa) are indicated on the left of each panel.

**Additional file 13:** **Supplementary Fig. 13**. ITC experiments of binding isotherm curve obtained from the titration of a solution of CtBP2 with Comp.11. Bottom: no binding is detected. Top: raw data of the measurements.

**Additional file 14:** **Supplementary Fig. 14**. Relative mRNA levels of *p16*^*INK4a*^, *p14*^*ARF*^*, p21*, and *CCND1* (upper panel), and *CtBP1/BARS* and *CtBP2* (lower panel), in A375MM cells **A**, and of murine *p21* and *CCND1* (*mp21* and *mCCND1, *upper panel), *CtBP1/BARS* and *CtBP2* (lower panel), in B16F10 cells **B**, measured by real time PCR after 24 h of treatment with DMSO (Ctr) or MTOB (5 mM) or HIPP (50 μM) or PPγ (5 μM) or Comp.11 (15 μM). *GAPDH *is used as housekeeping gene. Data are means ± SD of three independent experiments. **P ≤ 0.01, ***P ≤ 0.001 *versus* DMSO (Ctr) or non-targeting (Student’s t-tests).

**Additional file 15:** **Supplementary Fig**. **15**. Relative mRNA levels of the reported genes measured by real time PCR in A375MM cells **A** and **B**, and in B16F10 cells **C** and **D**, after 24 h of treatment with DMSO (Ctr) or MTOB (5 mM) or HIPP (50 μM) or PPγ (5 μM) or Comp.11 (15 μM). *GAPDH* is used as housekeeping gene. Data are means ± SD of three independent experiments. *P ≤ 0.05, ***P ≤ 0.001 *versus* DMSO (Ctr) or non-targeting (Student’s t-tests)

**Additional file 16:** **Supplementary Fig. 16**. Relative mRNA levels of epithelial markers (*E-cadherin*, *plakoglobin*, *β-cathenin*, *Desmoglein 2*, *Occludin*, *JAM-1*, *ZO1*) and mesenchymal markers (*N-cadherin*, *Vimentin* and *Versican*) in A375MM cells **A**, and in B16F10 cells **B**, measured by real time PCR after 24 h of treatment with DMSO (Ctr) or MTOB (5 mM) or HIPP (50 μM) or PPγ (5 μM) or Comp.11 (15 μM). *GAPDH *is used as housekeeping gene. Data are means ± SD of three independent experiments performed in triplicate. *P ≤ 0.05, **P ≤ 0.01, ***P ≤ 0.001 *versus* DMSO (Ctr) or non-targeting (Student’s t-tests).

**Additional file 17:** **Supplementary Fig. 17**. Analysis of CtBP1/BARS and CtBP2 depletion in A375MM and B16F10 melanoma cells subjected to wound closure assays and to Matrigel invasion assays reported in Fig. 8. Representative Western blotting with anti-CtBP1/BARS, anti-CtBP2 and anti-GAPDH antibodies (as indicated) of A375MM cells and B16F10 cells treated for 24 h with DMSO (vehicle control) or Comp.11 (15 μM) or transfected for 48 h with non-targeting or with CtBP1/BARS siRNAs or CtBP2 siRNAs. Molecular weight standards (kDa) are indicated on the left of each panel.

**Additional file 18**: **Supplementary Fig. 18**. Analysis of CtBP1/BARS and CtBP2 depletion in A375MM and B16F10 melanoma cells subjected to wound closure assays and to Matrigel invasion assays reported in Fig. 8. Representative Western blotting with anti-CtBP1/BARS, anti-CtBP2 and anti-GAPDH antibodies (as indicated) of A375MM cells and B16F10 cells treated for 24 h with DMSO (vehicle control) or Comp.11 (15 μM) or transfected for 48 h with non-targeting or with CtBP1/BARS siRNAs or CtBP2 siRNAs. Molecular weight standards (kDa) are indicated on the left of each panel.

**Additional File 19: Supplementary Fig. 19.** **A**. Waterfall plot of the fold-change in tumor volume compared with baseline [determined by caliper and calculated as (length $\times$width2)/2] of A375MM xenograft tumors after 2 week of Comp.11 treatment. **B**. Representative High Frequency Ultrasound (HFUS) images of tumors (yellow arrow) at 2 weeks of Comp.11 (20 mg/kg body weight) or vehicle treatment (3 mice for each treatment group). Tumor volumes are expressed as mean ± SEM, *P=0.04, Test Kruskal-Wallis followed by post hoc Tukey.

**Additional File 20: Supplementary Table 1. **List of oligonucleotides used in this study (human genes).

**Additional File 21: Supplementary Table 2. **List of oligonucleotides used in this study (murine genes).

## Data Availability

All data generated or analyzed during this study are included in this published article (and its supplementary information files).
