# Supplementary figures and images for: Identification and characterization of a new potent inhibitor targeting CtBP1/BARS in melanoma cells
Source: J Exp Clin Cancer Res. 2024 May 6;43:137. doi: 10.1186/s13046-024-03044-5 (PMC11071220; doi:10.1186/s13046-024-03044-5)

**A**

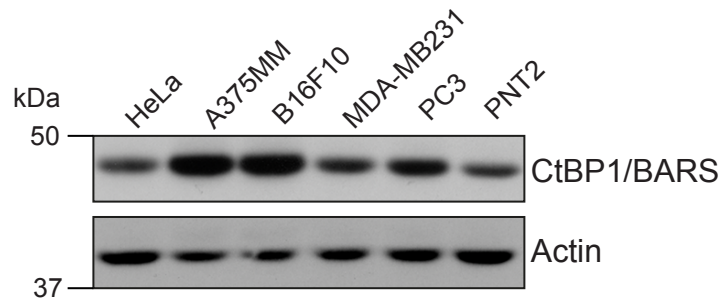

**B**

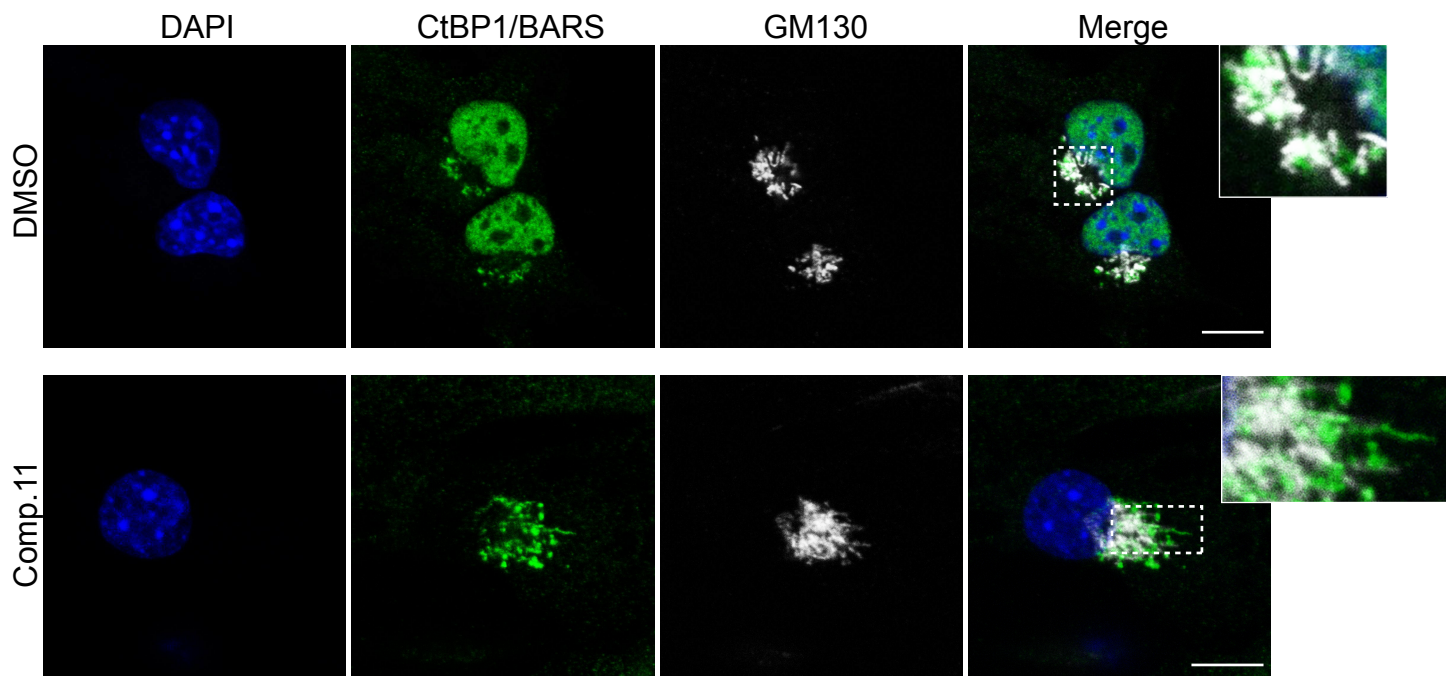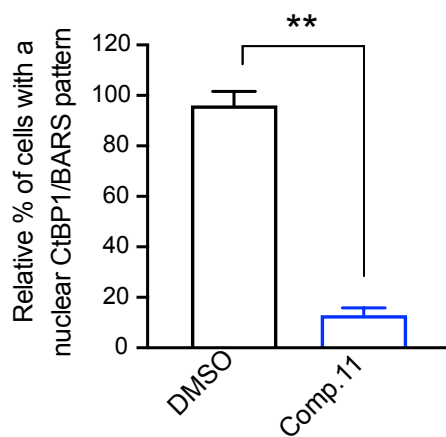

Supplement: Supplementary file 1 — Additional file 1: Supplementary Fig. 1. A. Representative Western blot analysis of CtBP1/BARS protein expression in HeLa, A375MM, B16F10, MDA-MB231 and PC3 cancer cells versus the epithelial normal PNT2 cells. β-actin is used as a loading control. Molecular weight standards (kDa) are indicated on the left of each panel. B. Representative confocal microscopy images of B16F10 cells treated with DMSO (vehicle control) or with Comp.11 (15 μM) for 2 h at 37°C. Cells were fixed and labeled with a polyclonal anti-CtBP1/BARS antibody (endogenous CtBP1/BARS; green) and with a monoclonal anti-GM130 antibody (used as a cis-Golgi marker; grey). Insets right: Magnification of Golgi area. Scale bars, 10 μm. Bottom; quantification of cells with a nuclear CtBP1/BARS pattern. Data are means ± SD of three independent experiments. **P ≤ 0.01 versus the vehicle DMSO (Student’s t-tests). [file 13046_2024_3044_MOESM1_ESM.pdf]

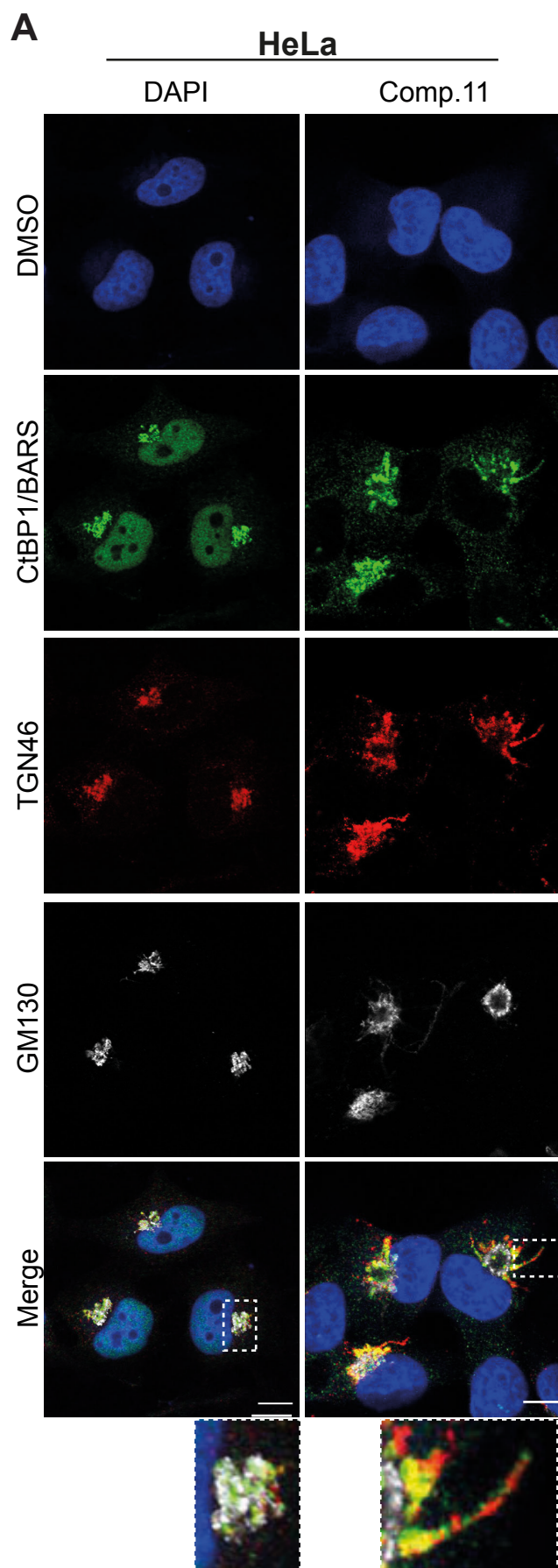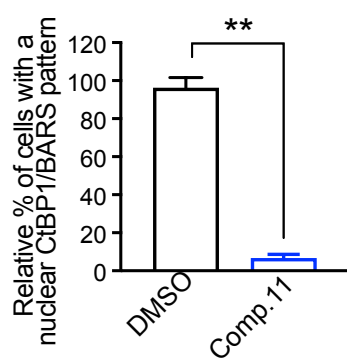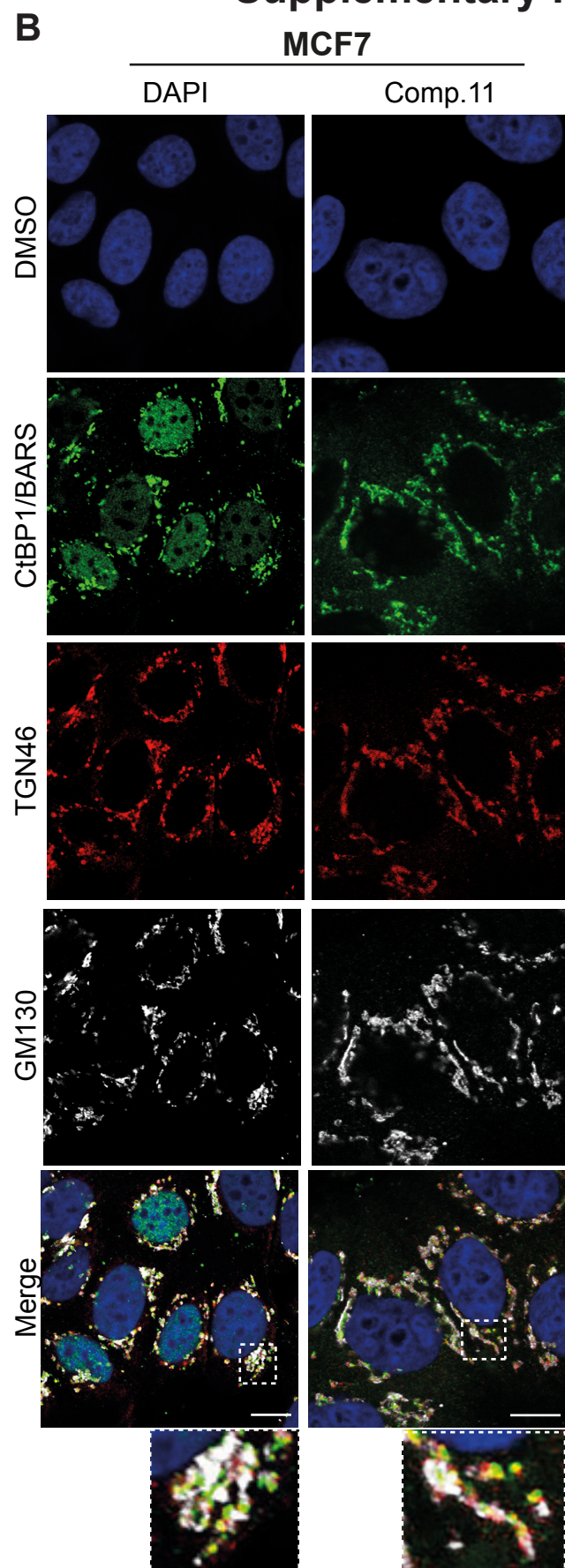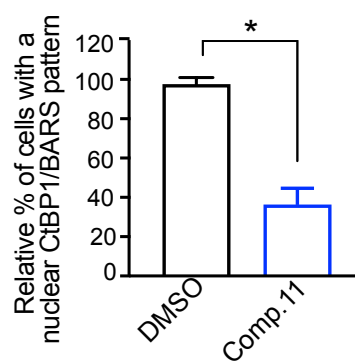

Supplement: Supplementary file 2 — Additional file 2: Supplementary Fig. 2. Representative confocal microscopy images of HeLa cells A, and MCF7 cells B, treated with DMSO (vehicle control) or with Comp.11 (15 μM) for 2 h at 37°C. Cells were fixed and stained for endogenous CtBP1/BARS (green), TGN46 (red; used as a TGN-Golgi marker) and GM130 (grey; used as a cis-Golgi marker). Insets: Magnification of Golgi area. Scale bars, 10 μm. Bottom; quantification of cells with a nuclear CtBP1/BARS pattern. Data are means ± SD of three independent experiments. *P ≤ 0.05, **P ≤ 0.01 versus DMSO (vehicle control) (Student’s t-tests). [file 13046_2024_3044_MOESM2_ESM.pdf]

**A**

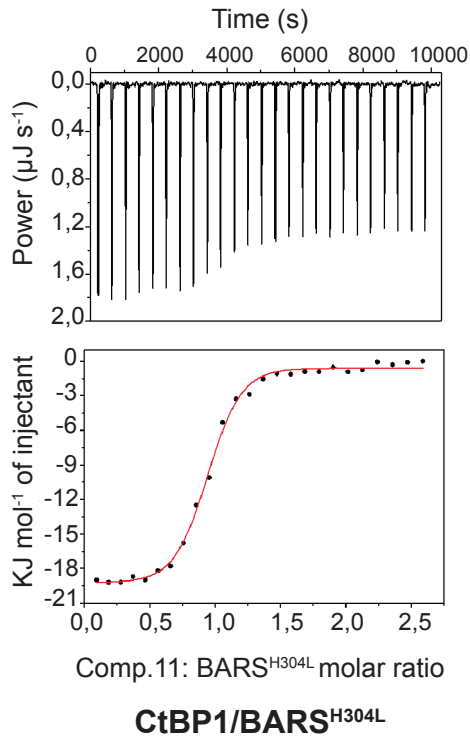

**B**

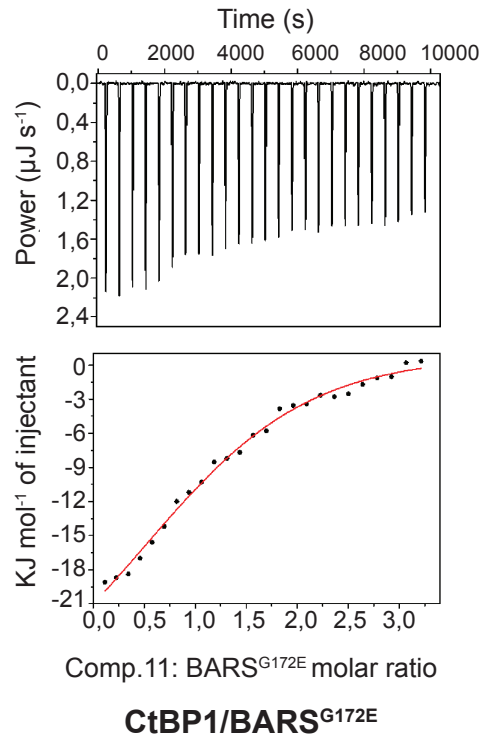

**C**

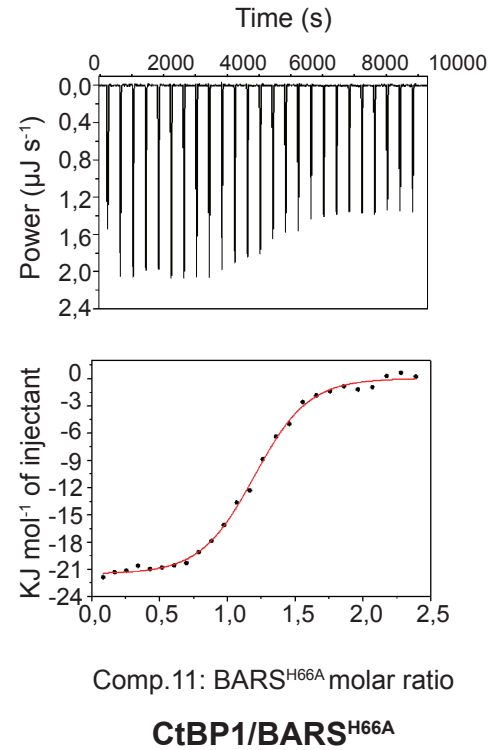

**D**

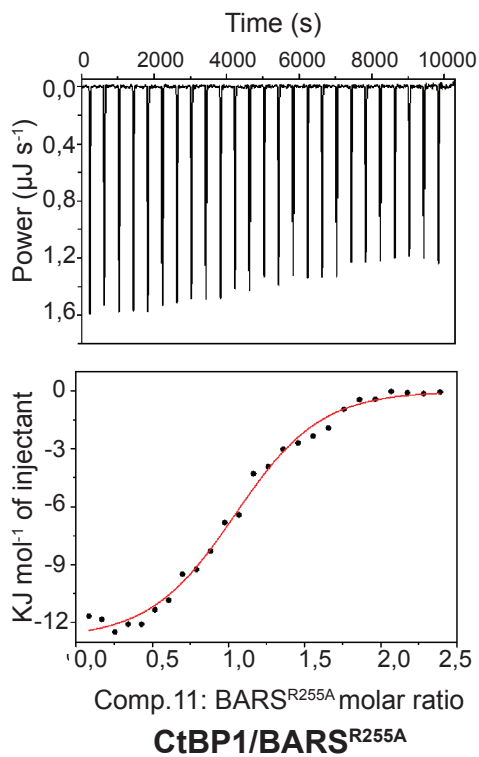

**E**

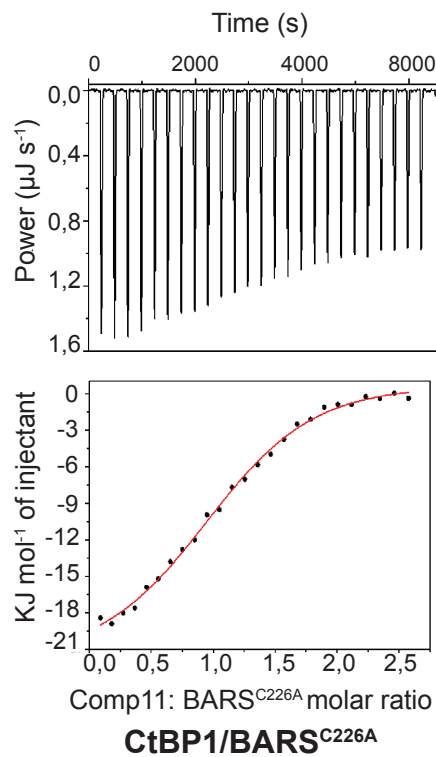

**F**

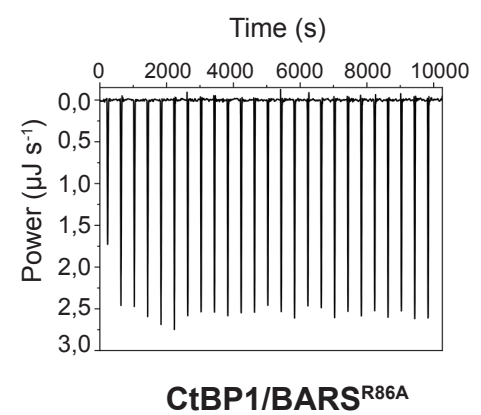

Supplement: Supplementary file 3 — Additional file 3: Supplementary Fig. 3. Top: ITC traces for the titration of the protein CtBP1/BARS mutants (as indicated) with the Comp.11 as ligand. Bottom: Integrated ITC data (black squares) as a function ligand/protein concentration ratio. The solid red line is the best fit of experimental data using the independent site binding model. [file 13046_2024_3044_MOESM3_ESM.pdf]

**A**

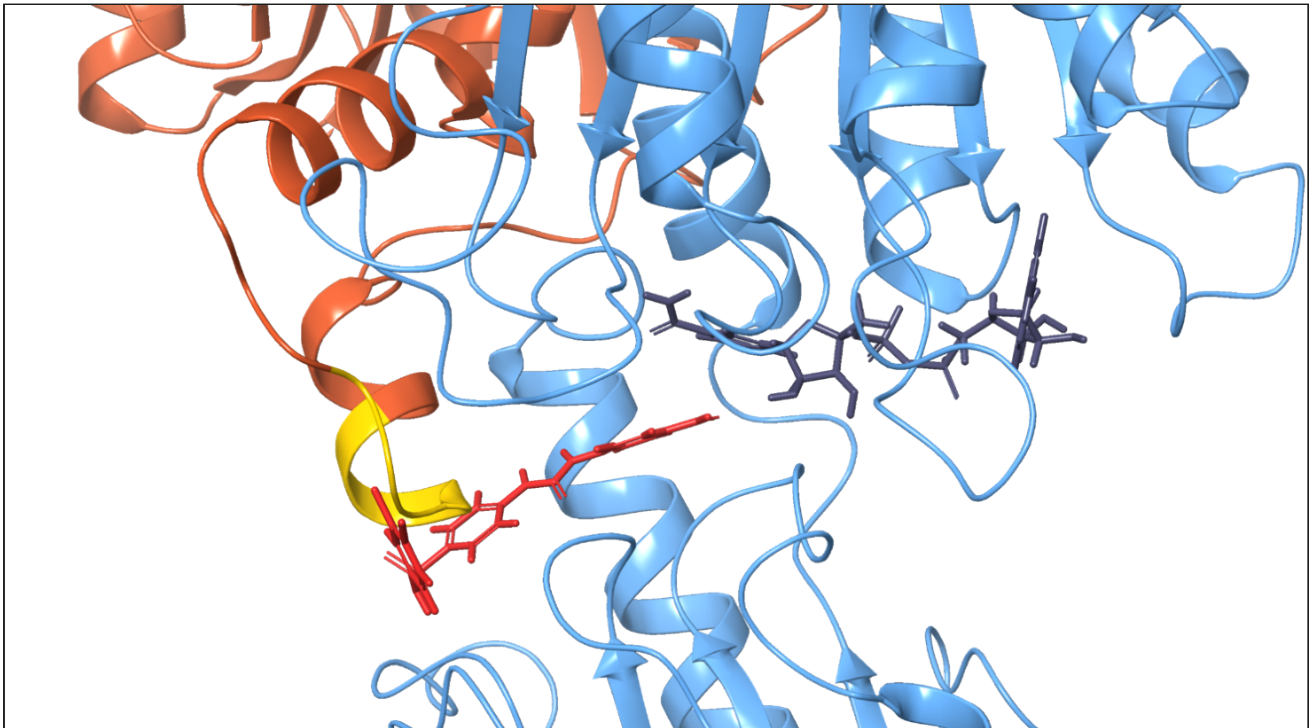

**B**

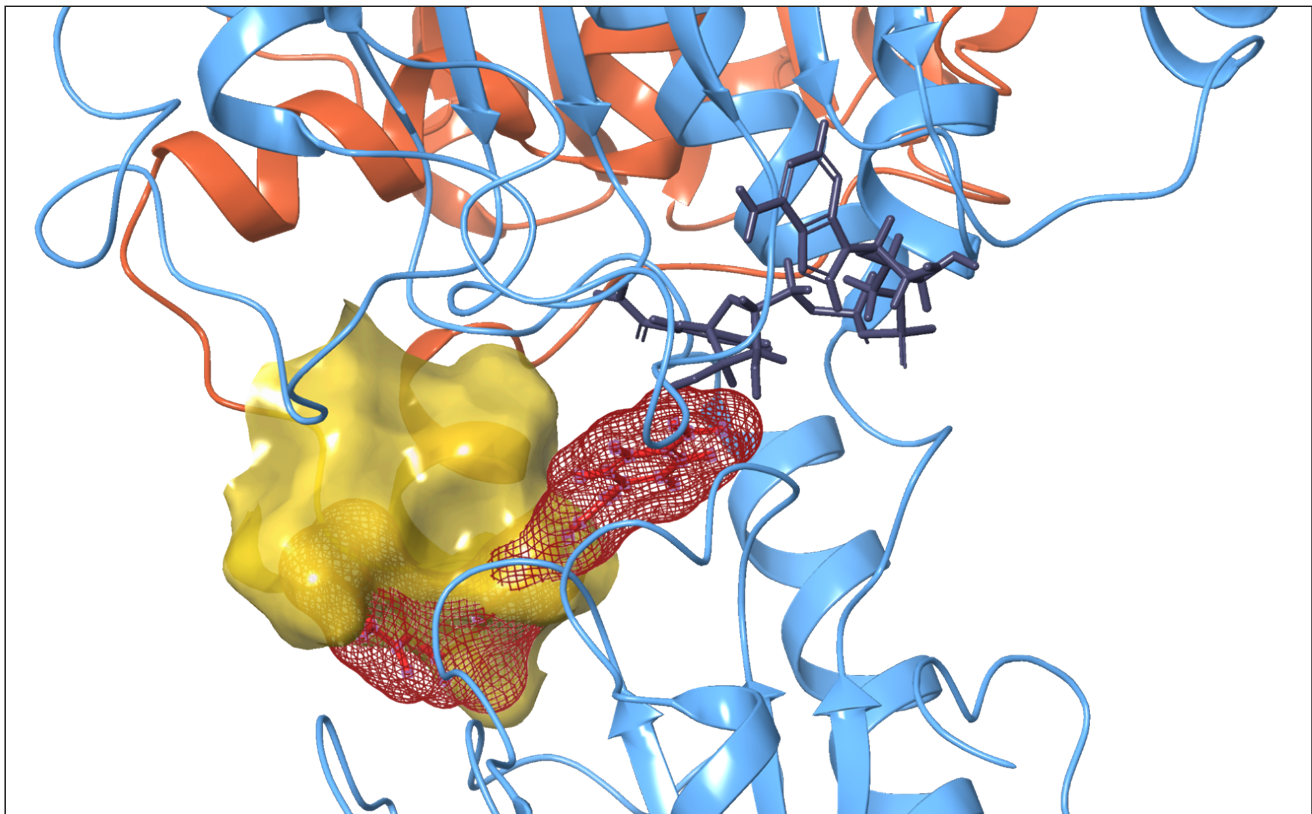

Supplement: Supplementary file 4 — Additional file 4: Supplementary Fig. 4. A. Representation of the predicted overlap between Comp.11 (in red) and the second monomer of CtBP1/BARS (orange chain) as would happen in the dimerization process. In yellow the residues along the second chain that collide with Comp.11. NADH is reported as well (in blue) and the first monomer of CtBP1/BARS is in light blue. B. Other representation of the incompatibility between Comp.11 (mashed red surface) and the second monomer of CtBP1/BARS (yellow solid surface). [file 13046_2024_3044_MOESM4_ESM.pdf]

**A**

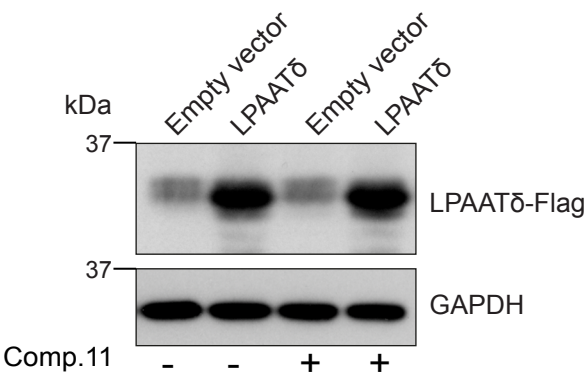

**B**

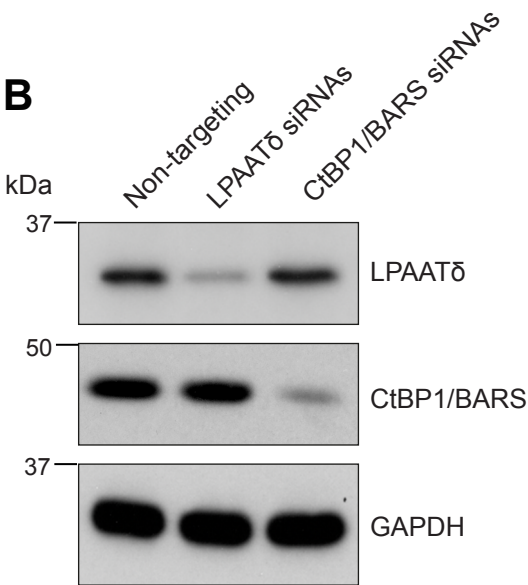

Supplement: Supplementary file 5 — Additional file 5: Supplementary Fig. 5. Evaluation of transfection efficiencies of post-nuclear supernatants used for the LPAAT assays in Fig. 3F. A. Representative Western blotting with anti-Flag and anti-GAPDH antibodies (as indicated) of post-nuclear supernatants from A375MM cells transfected for 48 h with an empty Flag-vector (Empty vector) or with LPAATδ–Flag (LPAATδ) and then incubated with 15 μM of Comp.11 (+) or with DMSO (-) for 30 min at 25°C. B. Representative Western blotting with anti-LPAATδ, anti-CtBP1/BARS and anti-GAPDH antibodies (as indicated) of post-nuclear supernatants from A375MM cells transfected for 72 h with non-targeting or CtBP1/BARS siRNAs or LPAATδ siRNAs. Molecular weight standards (kDa) are indicated on the left of each panel. [file 13046_2024_3044_MOESM5_ESM.pdf]

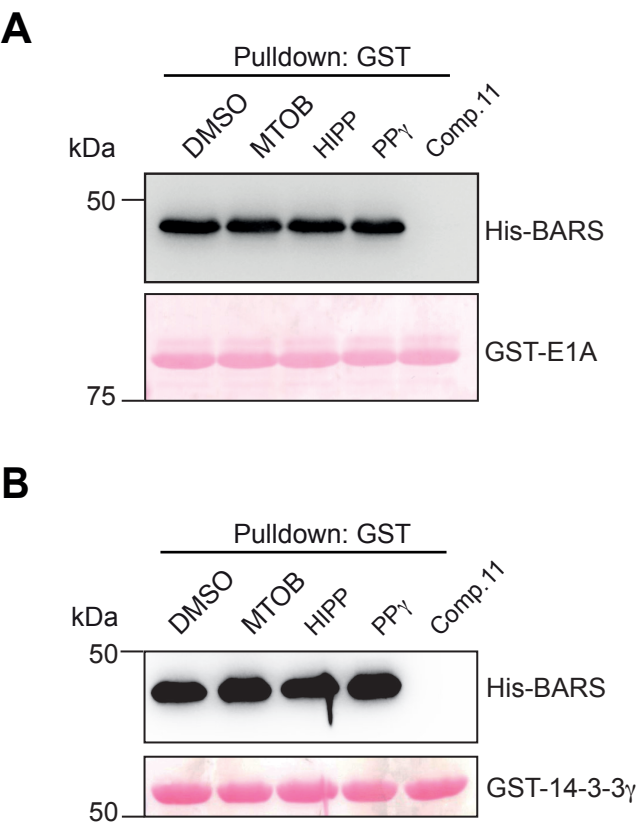

Supplement: Supplementary file 6 — Additional file 6: Supplementary Fig. 6. A. Representative GST pull-down of GST-E1A recombinant protein for His-CtBP1/BARS. His-CtBP1/BARS protein was first incubated with DMSO (vehicle control) or with 5 mM MTOB or 50 µM HIPP or 5 µM PPγ or 15 µM Comp.11 (1 h, 4°C in a wheel), and then incubated with GST-E1A (2 h, 4°C in a wheel; see Methods). The bound proteins to the glutathione Sepharose beads were eluted and analyzed by western blotting with anti-His monoclonal antibody (top), with pulled-down proteins revealed by Ponceau-S staining (bottom). B. Representative GST pull-down of GST-14-3-3γ recombinant protein for His-CtBP1/BARS. His-CtBP1/BARS protein was pre-incubated with DMSO (vehicle control) or with 5 mM MTOB or 50 µM HIPP or 5 µM PPγ or 15 μM Comp.11 (1 h, 4°C in a wheel), and then incubated with GST-14-3-3γ (2 h, 4°C in a wheel; see Methods). The bound proteins to the glutathione Sepharose beads were eluted and analyzed by western blotting with anti-His monoclonal antibody (bottom), with pulled-down proteins revealed by Ponceau-S staining (bottom). Data are representative of three independent experiments. [file 13046_2024_3044_MOESM6_ESM.pdf]

Supplementary Fig. 7

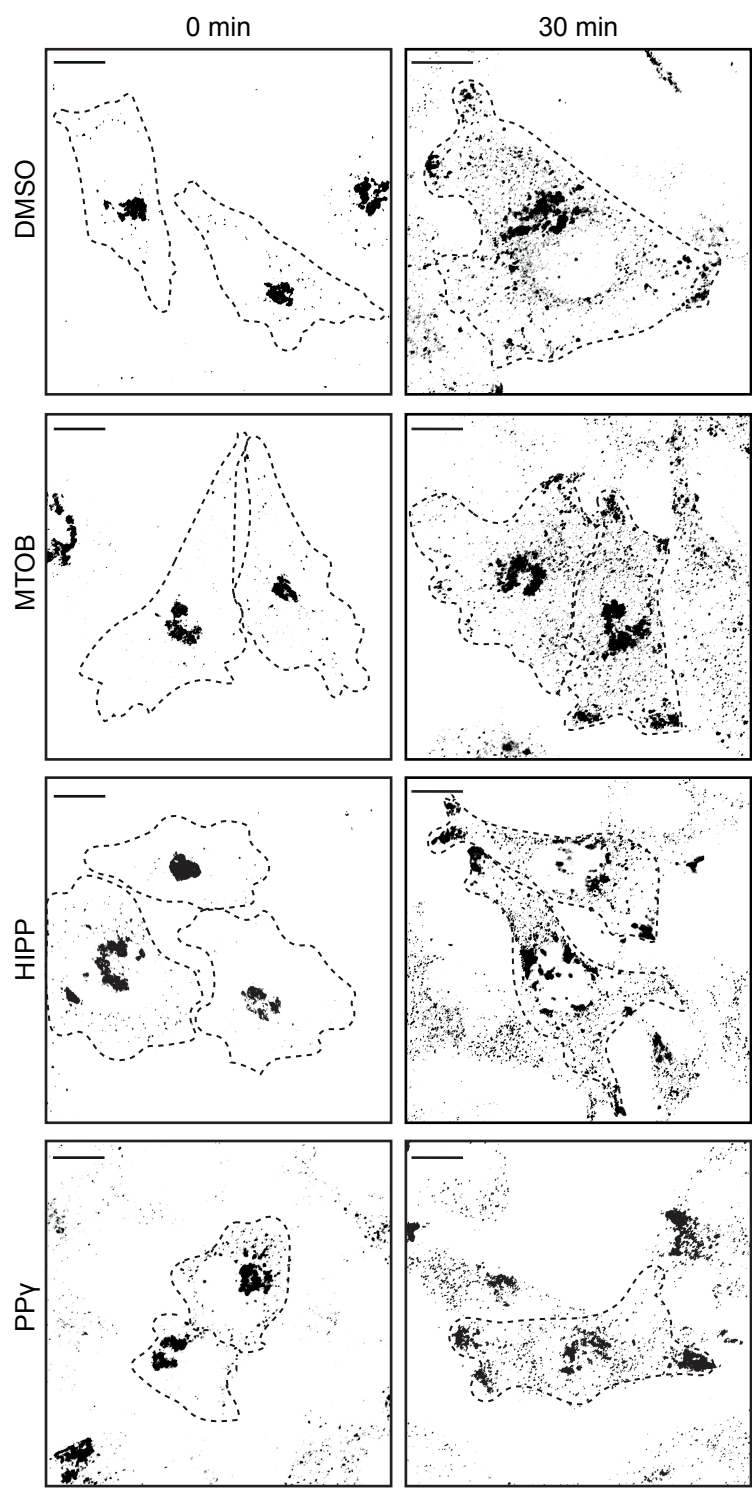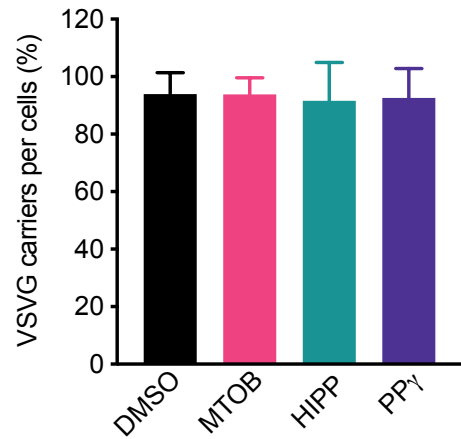

Supplement: Supplementary file 7 — Additional file 7: Supplementary Fig. 7. Representative confocal microscopy images of A375MM cells infected with VSV and subjected to TGN-exit assay with 0.5% tannic acid. The cells were treated with DMSO (vehicle control) or with 5 mM MTOB or 50 µM HIPP or 5 µM PPγ or 15 μM Comp.11 for 2 h at the 20°C block during the TGN-exit assay (see Methods). The cells were fixed following the 20°C (0 min) or 30 min after the shift to 32°C, and processed for immunofluorescence with anti-VSVG (p5D4) antibody, to monitor formation of VSVG-containing carriers. Dotted lines show cell borders. Scale bars, 10 μm. Quantification of VSVG-positive carriers (right). Data are means ± SD of three independent experiments. These data are not statistically significant (Student’s t-tests). [file 13046_2024_3044_MOESM7_ESM.pdf]

**A**

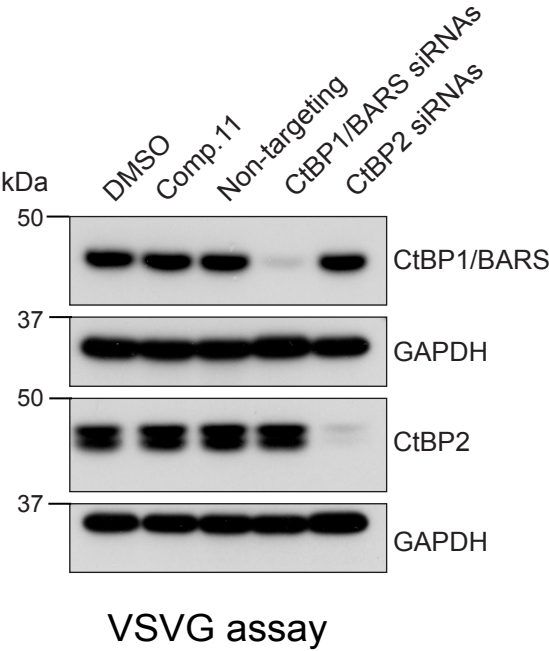

**B**

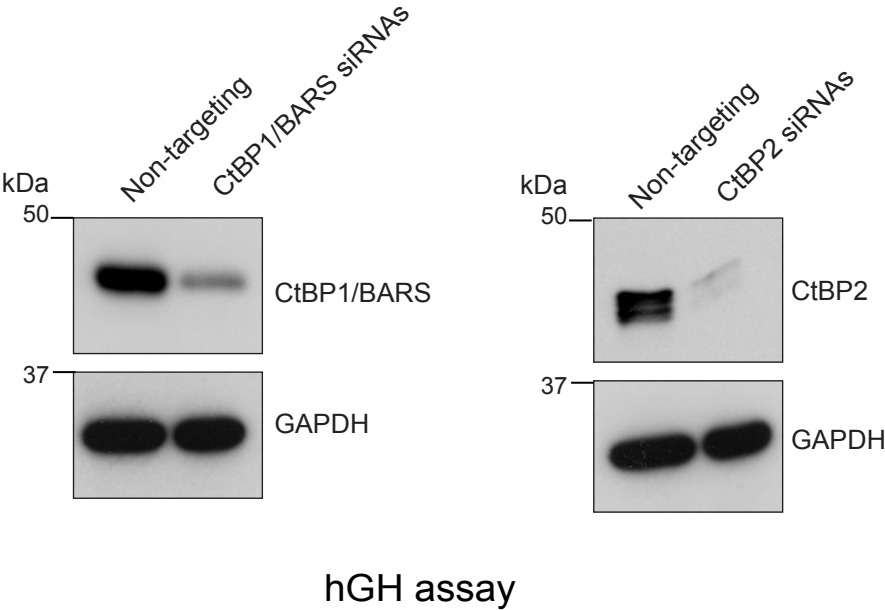

Supplement: Supplementary file 8 — Additional file 8: Supplementary Fig. 8. Analysis of CtBP1/BARS and CtBP2 depletion in A375MM cells subjected to the VSV-traffic pulse A, and in HeLa cells stably transfected with hGH-FM–GFP and subjected to a secretion assay B, as reported in Fig. 4. Representative Western blotting with anti-CtBP1/BARS, anti-CtBP2 and anti-GAPDH antibodies (as indicated). Molecular weight standards (kDa) are indicated on the left of each panel. [file 13046_2024_3044_MOESM8_ESM.pdf]

**A**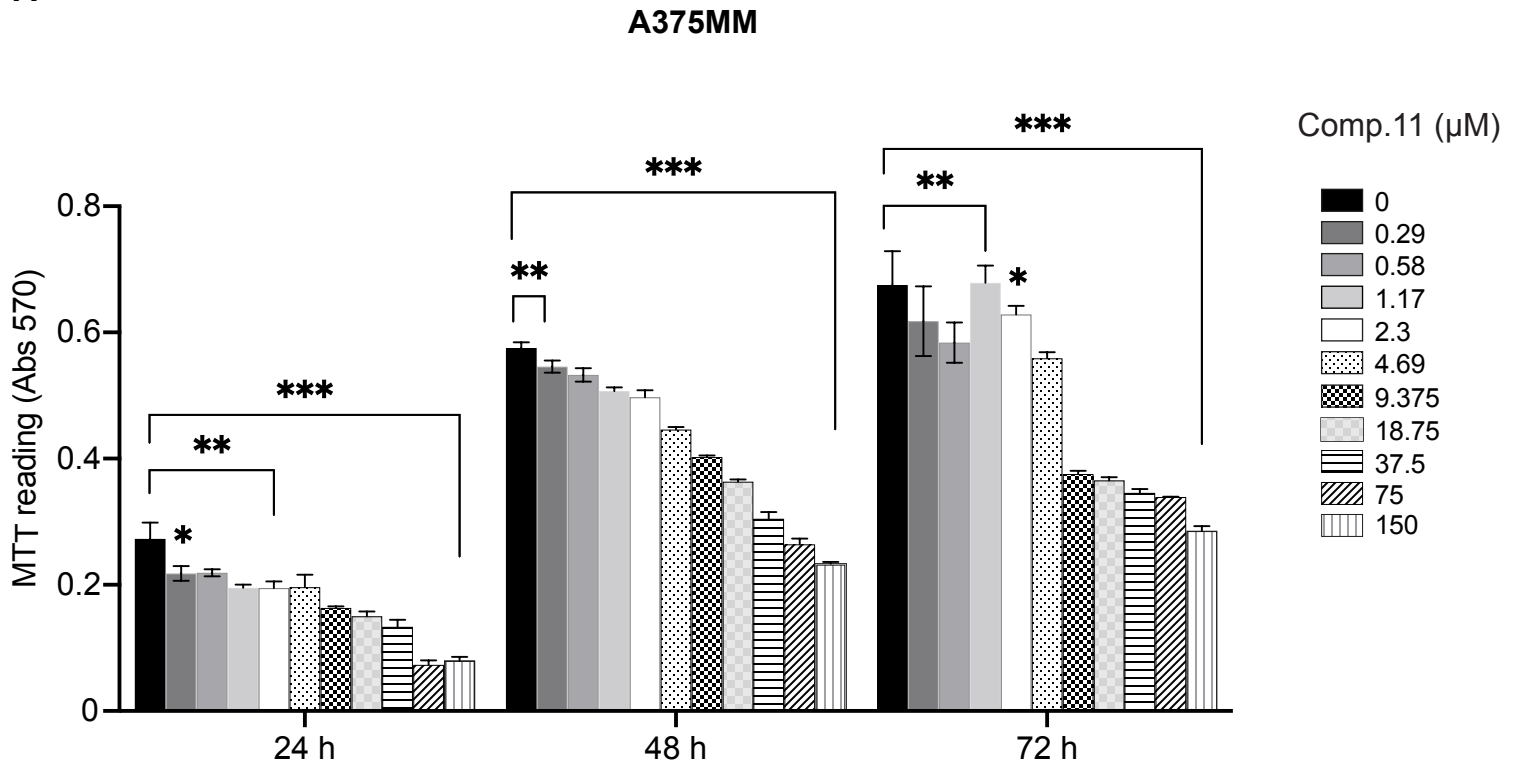**B**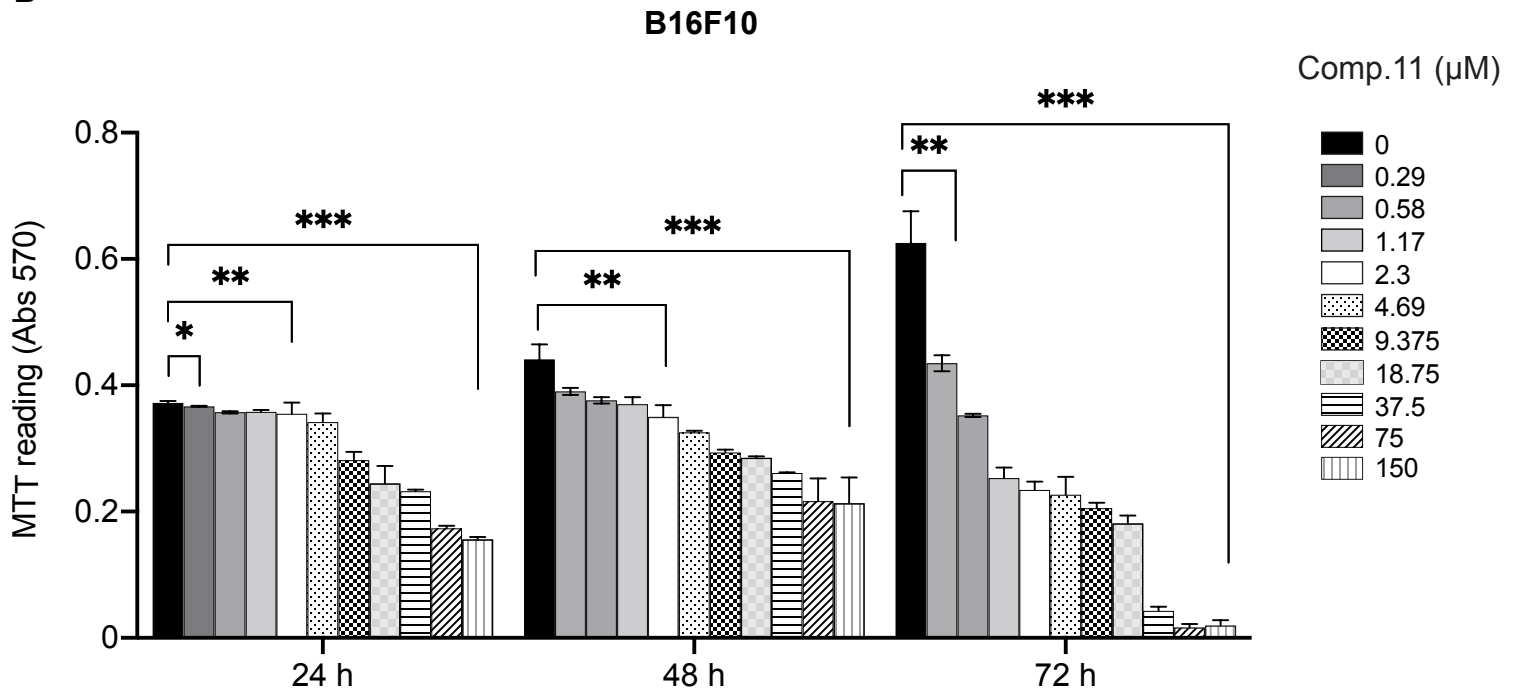

Supplement: Supplementary file 9 — Additional file 9: Supplementary Fig. 9. Comp.11 inhibits cell proliferation in melanoma cell lines. A, A375MM cells and B, B16F10 cells were treated with increasing concentrations of Comp.11 (from 0 to 150 µM) for 24 h, 48 h and 72 h and their viability was evaluated according to MTT assay (as reported in Fig. 5A and 5E). Absorbance was detected at 570 nm with a microplate reader. Data are expressed in MTT Reading and are means ± SD of three independent experiments performed in duplicate. *P ≤ 0.05, **P ≤ 0.01, ***P ≤ 0.001 versus Ctr (Student’s t-tests). [file 13046_2024_3044_MOESM9_ESM.pdf]

A375MM

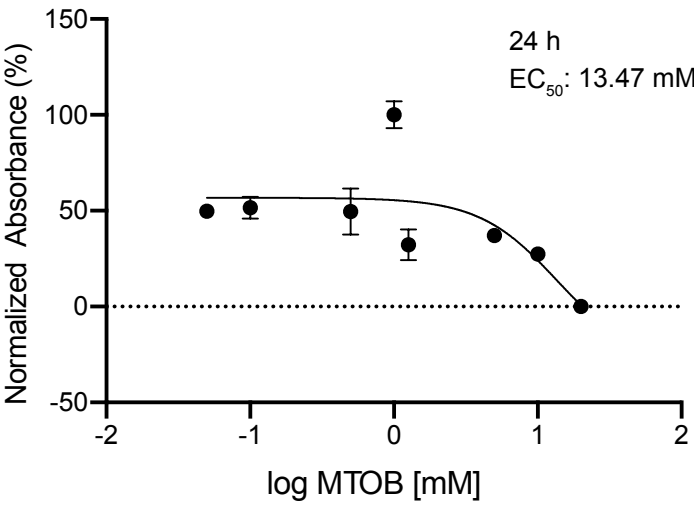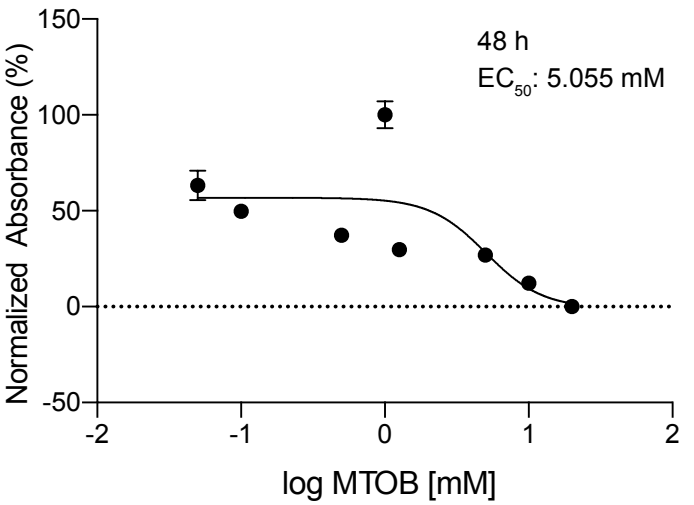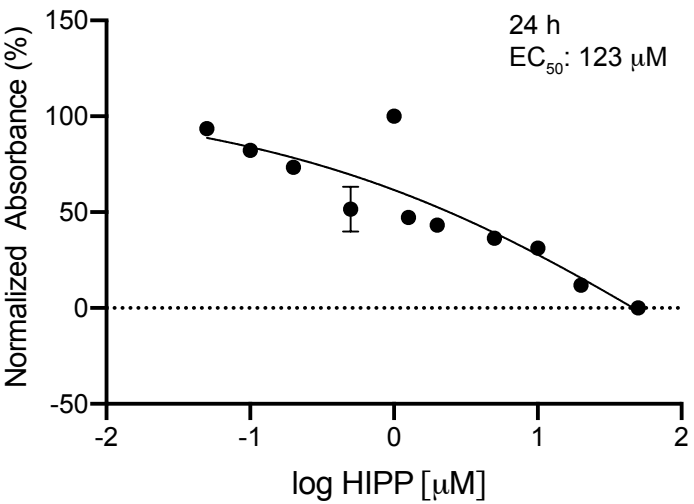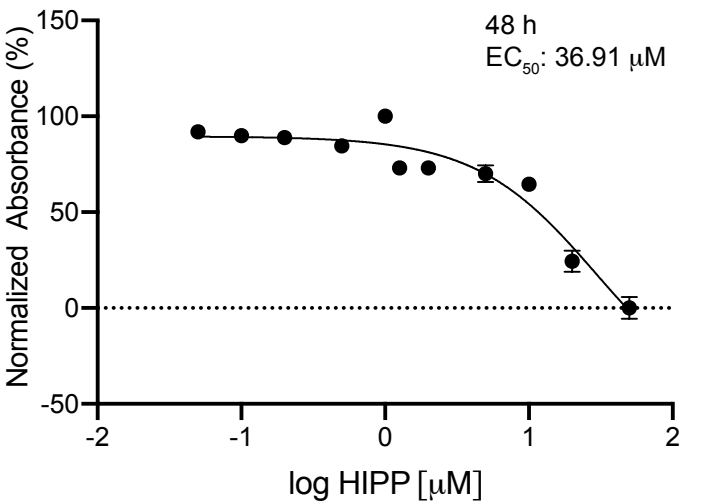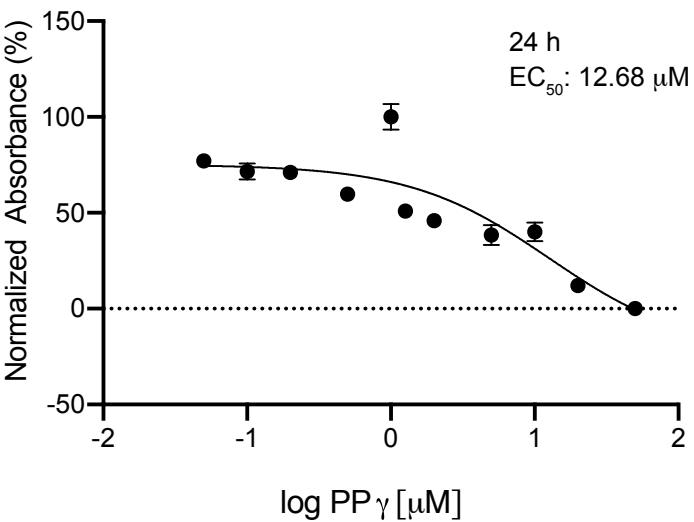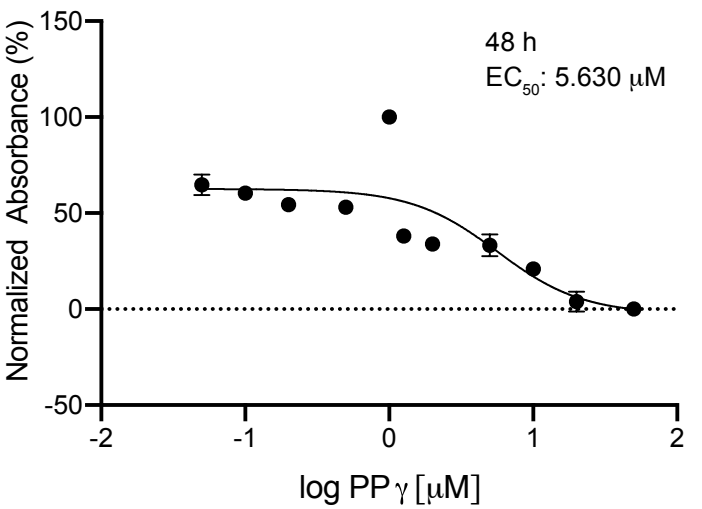

Supplement: Supplementary file 10 — Additional file 10: Supplementary Fig. 10. A375MM cells were treated with increasing concentrations of MTOB (from 0 to 20 mM) or HIPP (from 0 to 150 µM) or PPγ (from 0 to 150 µM) for 24 h and 48 h and their viability was evaluated according to MTT assay. The graphs represent the dose-response of log10 concentrations of MTOB, HIPP or PPγ (as indicated) versus normalized optical intensity at 570 nm. EC50 values of MTOB, HIPP or PPγ were calculated and reported as indicated. [file 13046_2024_3044_MOESM10_ESM.pdf]

B16F10

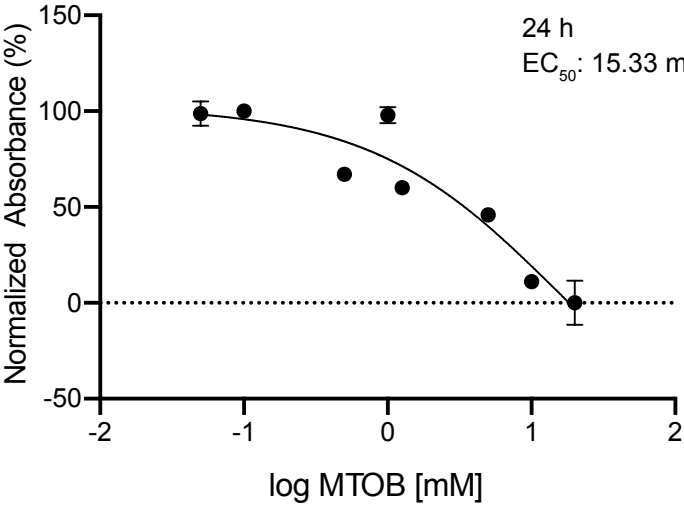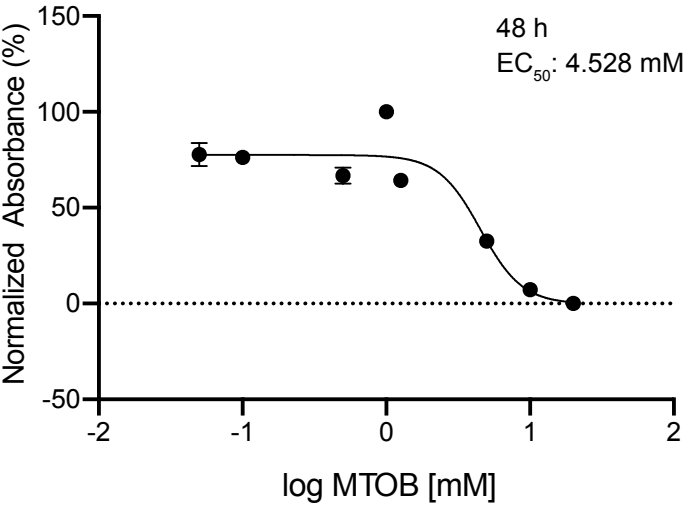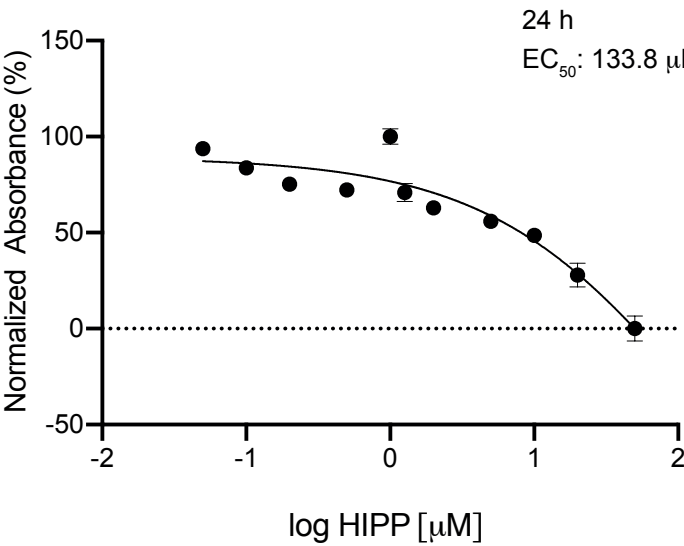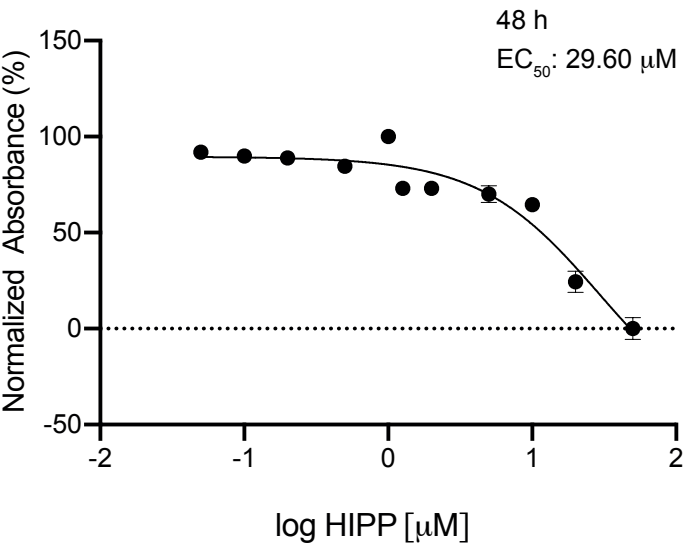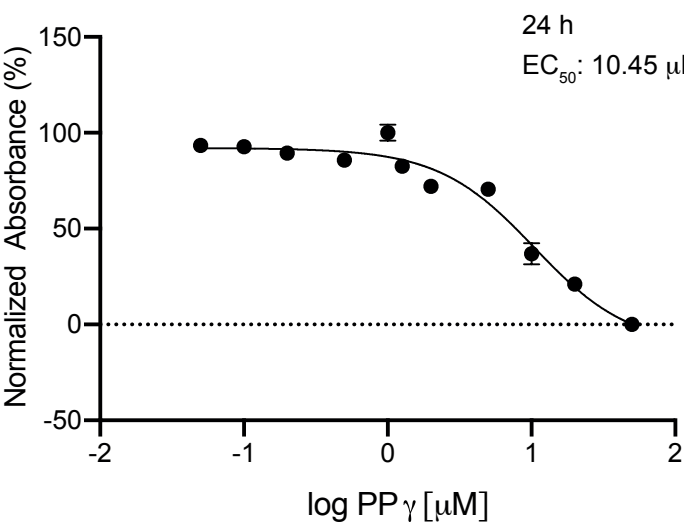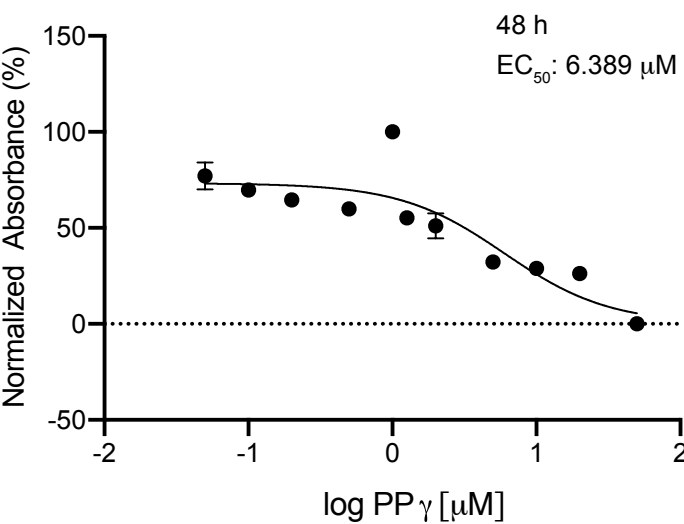

Supplement: Supplementary file 11 — Additional file 11: Supplementary Fig. 11. B16F10 cells were treated with increasing concentrations of MTOB (from 0 to 20 mM) or HIPP (from 0 to 150 µM) or PPγ (from 0 to 150 µM) for 24 h and 48 h and their viability was evaluated according to MTT assay. The graphs represent the dose-response of log10 concentrations of MTOB, HIPP or PPγ (as indicated) versus normalized optical intensity at 570 nm. EC50 values of MTOB, HIPP or PPγ were calculated and reported as indicated. [file 13046_2024_3044_MOESM11_ESM.pdf]

**A** Cell Cycle Analysis

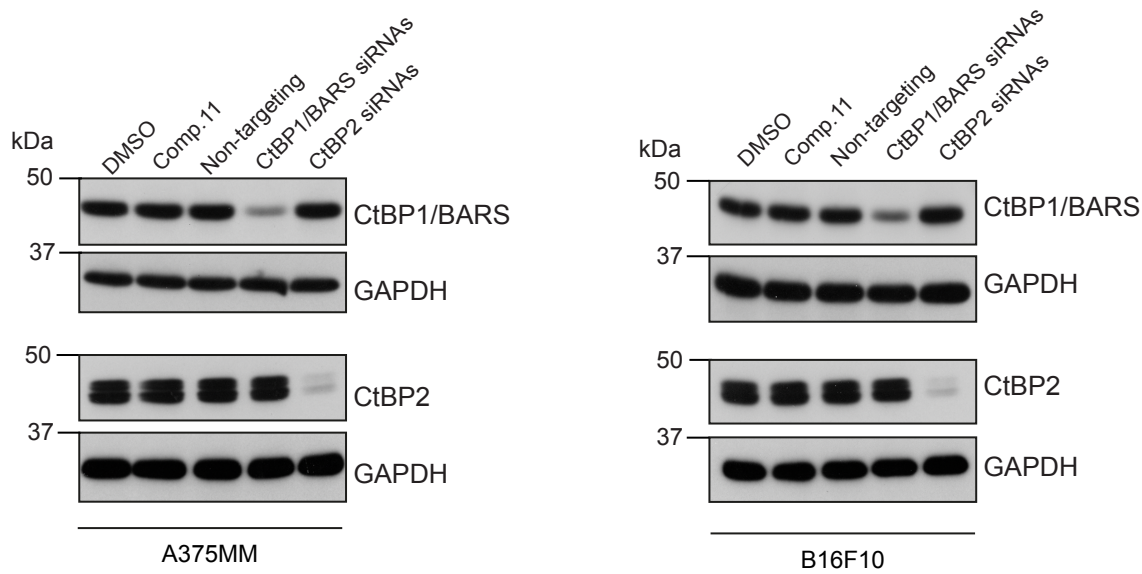

**B** Apoptosis Analysis

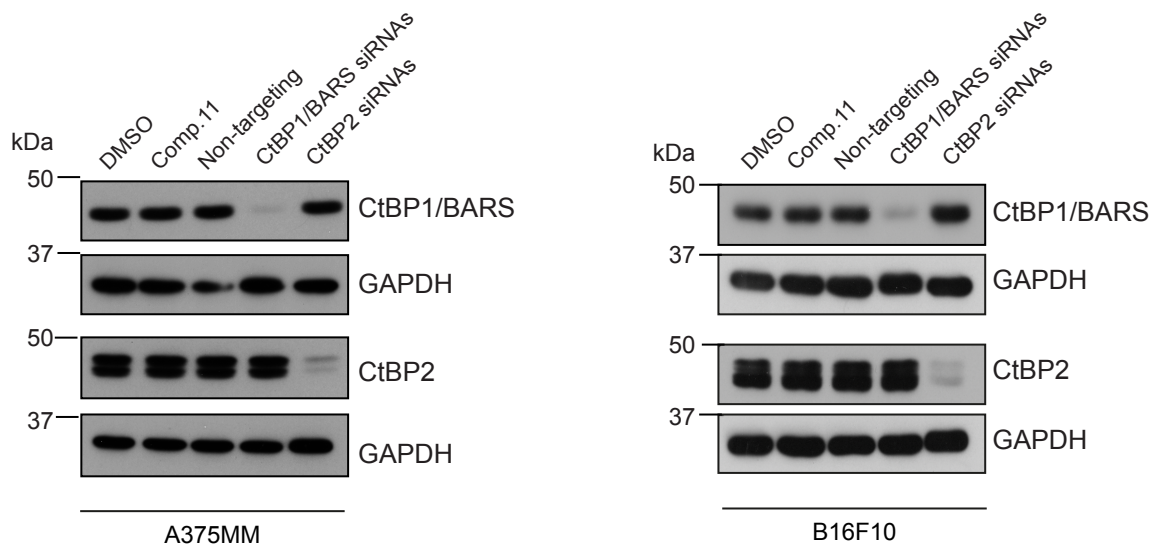

Supplement: Supplementary file 12 — Additional file 12: Supplementary Fig. 12. Analysis of CtBP1/BARS and CtBP2 depletion in A375MM and B16F10 melanoma cells subjected to the cell cycle analysis (A) and to the apoptosis analysis (B) as reported in Fig. 5 and Fig. 6. Representative Western blotting with anti-CtBP1/BARS, anti-CtBP2 and anti-GAPDH antibodies (as indicated) of A375MM cells and B16F10 cells treated for 24 h with DMSO (vehicle control) or Comp.11 (15 μM) or transfected for 48 h with non-targeting or with CtBP1/BARS siRNAs or CtBP2 siRNAs. Molecular weight standards (kDa) are indicated on the left of each panel. [file 13046_2024_3044_MOESM12_ESM.pdf]

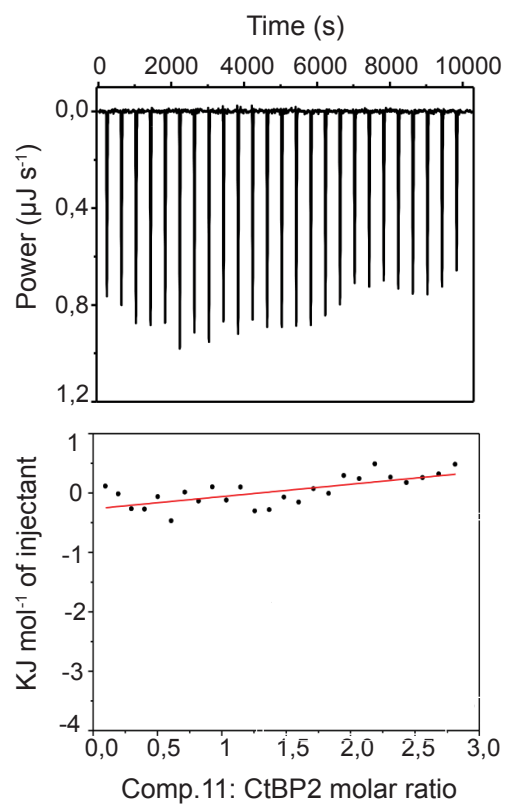

Supplement: Supplementary file 13 — Additional file 13: Supplementary Fig. 13. ITC experiments of binding isotherm curve obtained from the titration of a solution of CtBP2 with Comp.11. Bottom: no binding is detected. Top: raw data of the measurements. [file 13046_2024_3044_MOESM13_ESM.pdf]

**A**

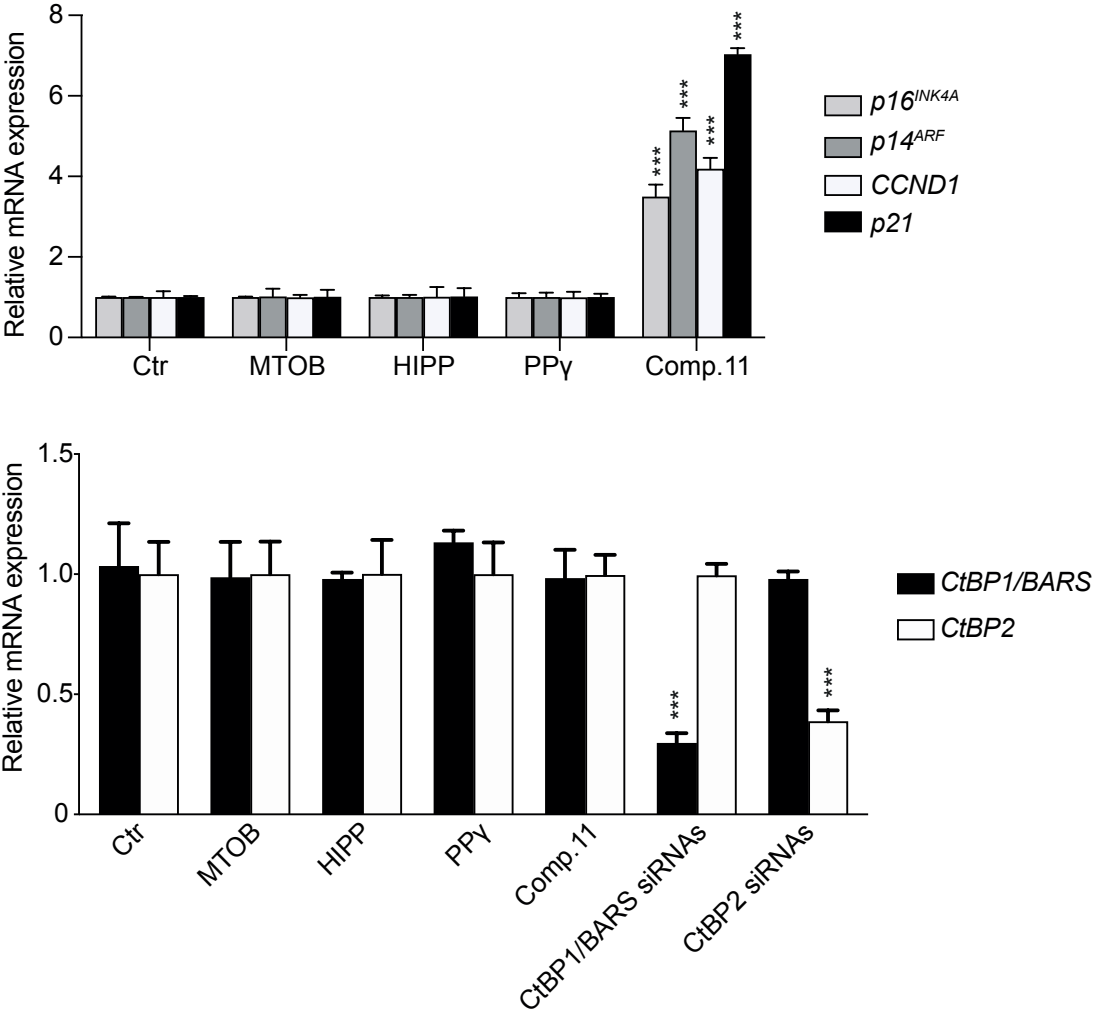

**B**

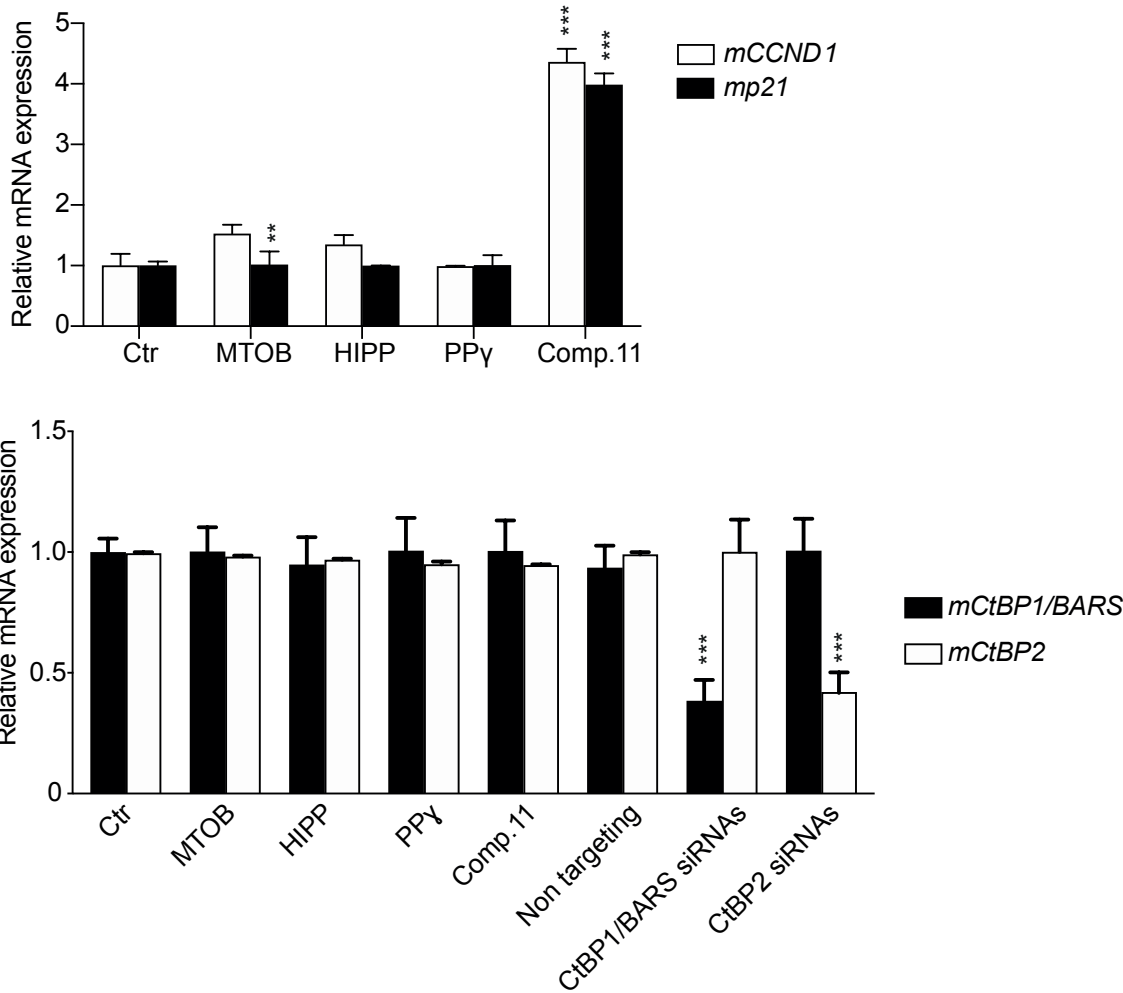

Supplement: Supplementary file 14 — Additional file 14: Supplementary Fig. 14. Relative mRNA levels of p16INK4a, p14ARF, p21, and CCND1 (upper panel), and CtBP1/BARS and CtBP2 (lower panel), in A375MM cells A, and of murine p21 and CCND1 (mp21 and mCCND1, upper panel), CtBP1/BARS and CtBP2 (lower panel), in B16F10 cells B, measured by real time PCR after 24 h of treatment with DMSO (Ctr) or MTOB (5 mM) or HIPP (50 μM) or PPγ (5 μM) or Comp.11 (15 μM). GAPDH is used as housekeeping gene. Data are means ± SD of three independent experiments. **P ≤ 0.01, ***P ≤ 0.001 versus DMSO (Ctr) or non-targeting (Student’s t-tests). [file 13046_2024_3044_MOESM14_ESM.pdf]

A

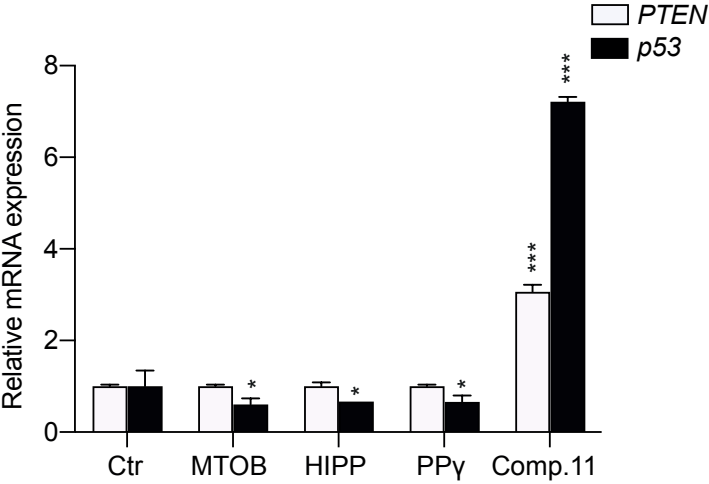

B

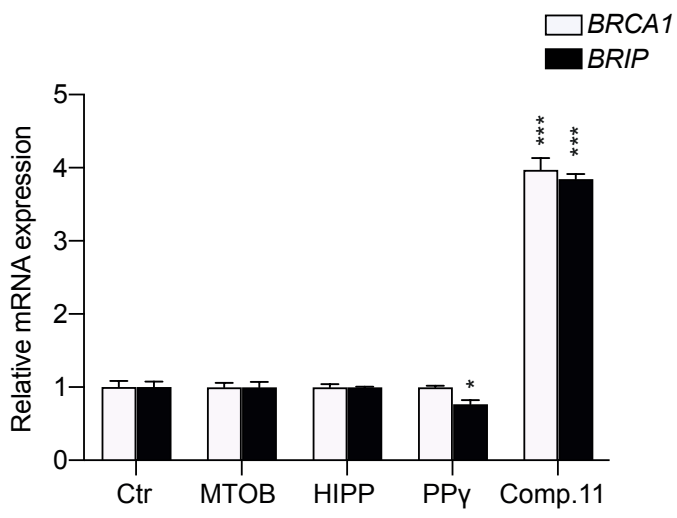

C

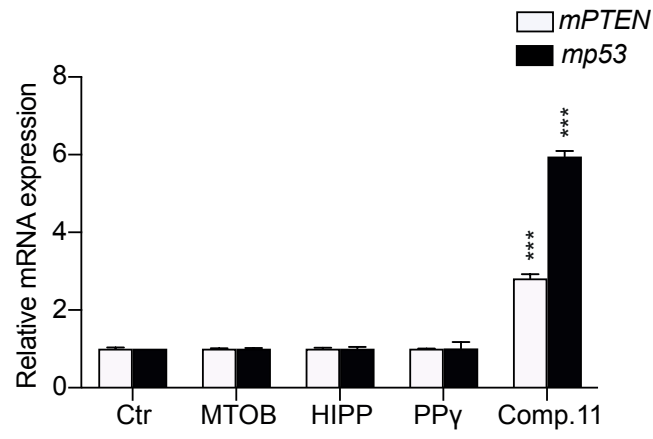

D

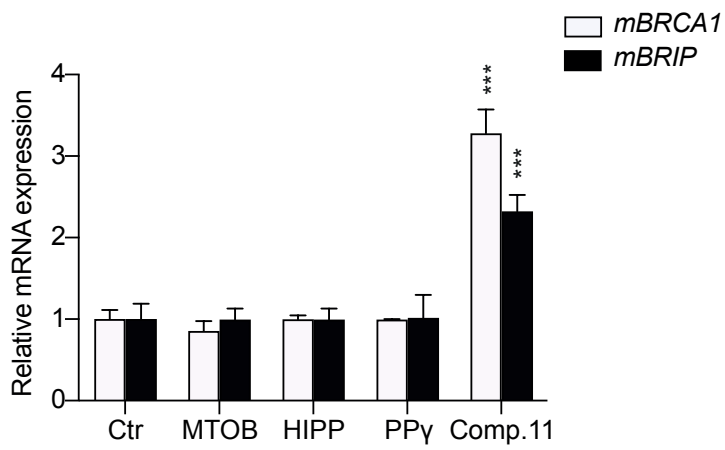

Supplement: Supplementary file 15 — Additional file 15: Supplementary Fig. 15. Relative mRNA levels of the reported genes measured by real time PCR in A375MM cells A and B, and in B16F10 cells C and D, after 24 h of treatment with DMSO (Ctr) or MTOB (5 mM) or HIPP (50 μM) or PPγ (5 μM) or Comp.11 (15 μM). GAPDH is used as housekeeping gene. Data are means ± SD of three independent experiments. *P ≤ 0.05, ***P ≤ 0.001 versus DMSO (Ctr) or non-targeting (Student’s t-tests) [file 13046_2024_3044_MOESM15_ESM.pdf]

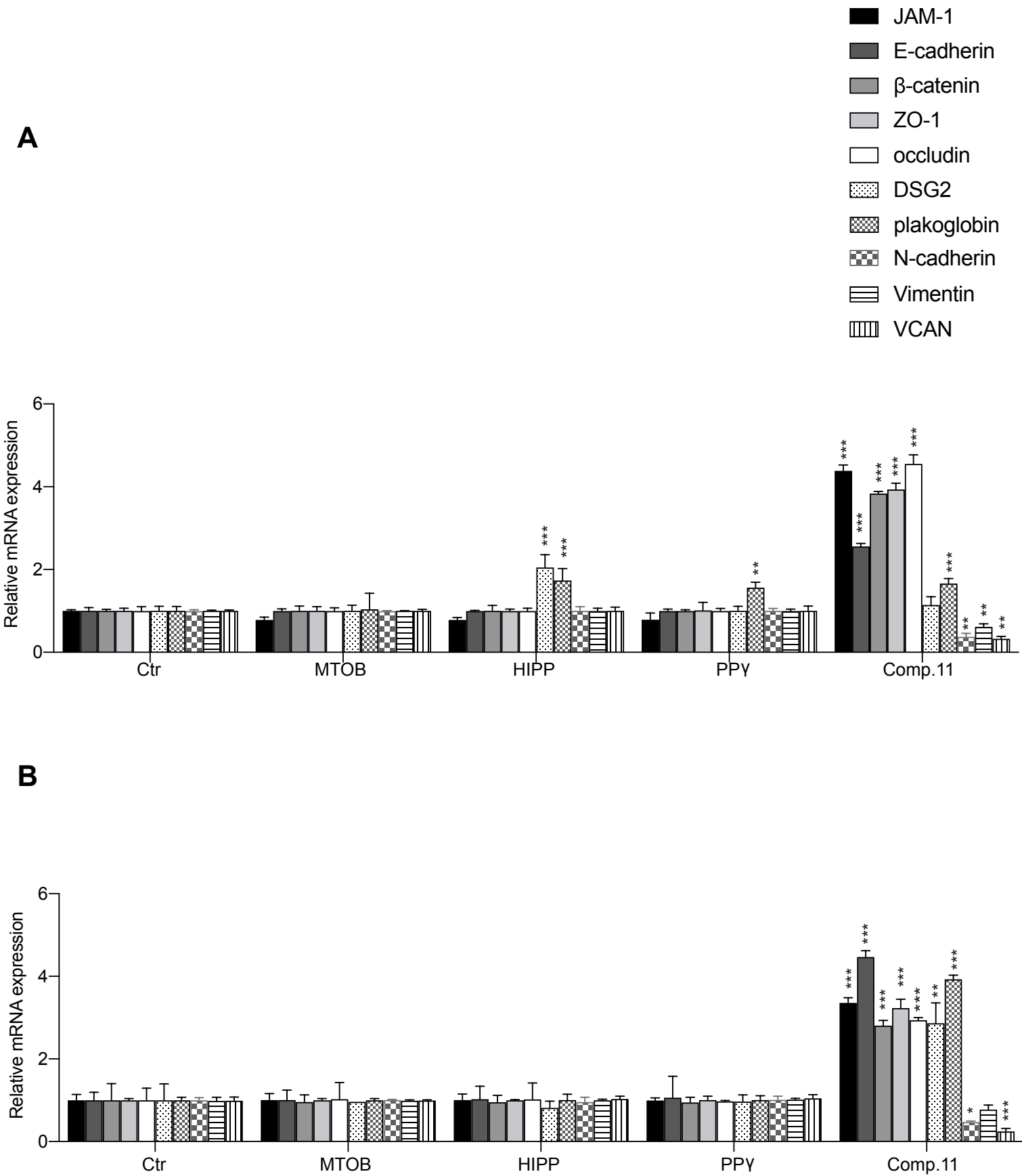

Supplement: Supplementary file 16 — Additional file 16: Supplementary Fig. 16. Relative mRNA levels of epithelial markers (E-cadherin, plakoglobin, β-cathenin, Desmoglein 2, Occludin, JAM-1, ZO1) and mesenchymal markers (N-cadherin, Vimentin and Versican) in A375MM cells A, and in B16F10 cells B, measured by real time PCR after 24 h of treatment with DMSO (Ctr) or MTOB (5 mM) or HIPP (50 μM) or PPγ (5 μM) or Comp.11 (15 μM). GAPDH is used as housekeeping gene. Data are means ± SD of three independent experiments performed in triplicate. *P ≤ 0.05, **P ≤ 0.01, ***P ≤ 0.001 versus DMSO (Ctr) or non-targeting (Student’s t-tests). [file 13046_2024_3044_MOESM16_ESM.pdf]

Supplementary Fig. 17

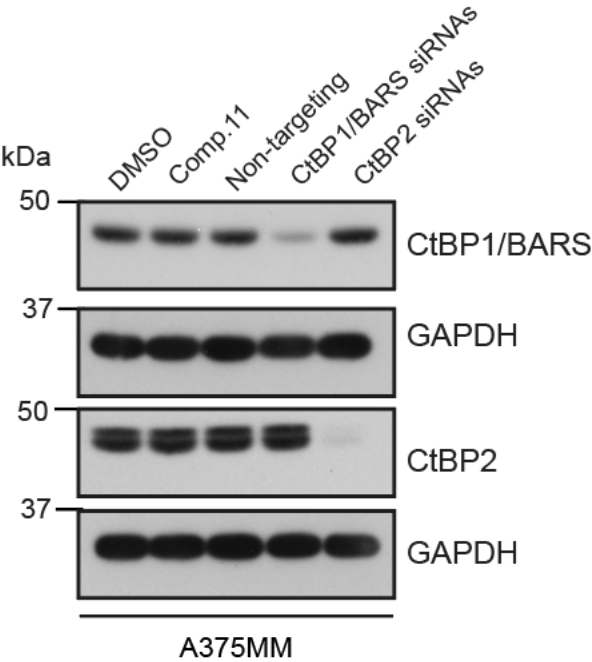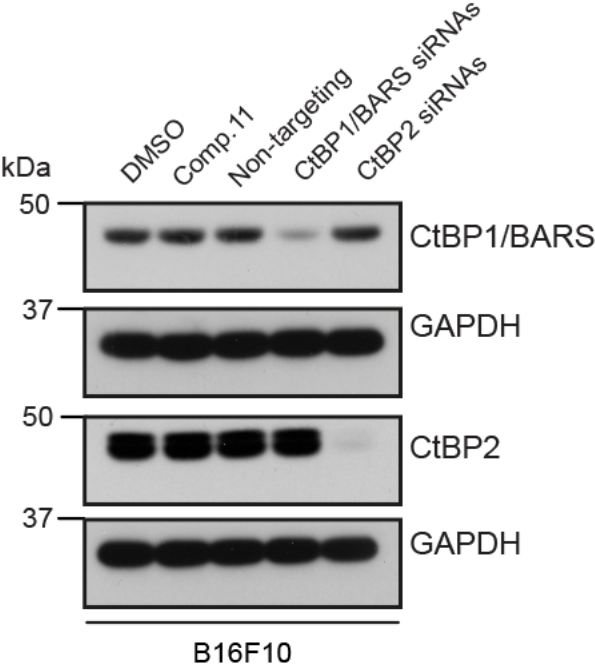

Supplement: Supplementary file 17 — Additional file 17: Supplementary Fig. 17. Analysis of CtBP1/BARS and CtBP2 depletion in A375MM and B16F10 melanoma cells subjected to wound closure assays and to Matrigel invasion assays reported in Fig. 8. Representative Western blotting with anti-CtBP1/BARS, anti-CtBP2 and anti-GAPDH antibodies (as indicated) of A375MM cells and B16F10 cells treated for 24 h with DMSO (vehicle control) or Comp.11 (15 μM) or transfected for 48 h with non-targeting or with CtBP1/BARS siRNAs or CtBP2 siRNAs. Molecular weight standards (kDa) are indicated on the left of each panel. [file 13046_2024_3044_MOESM17_ESM.pdf]

**A**

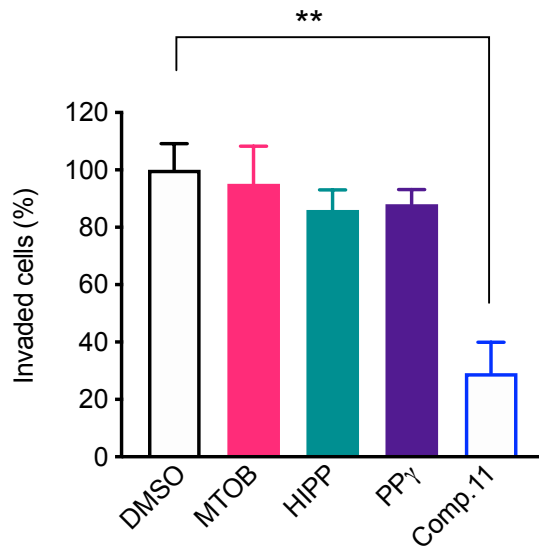

**B**

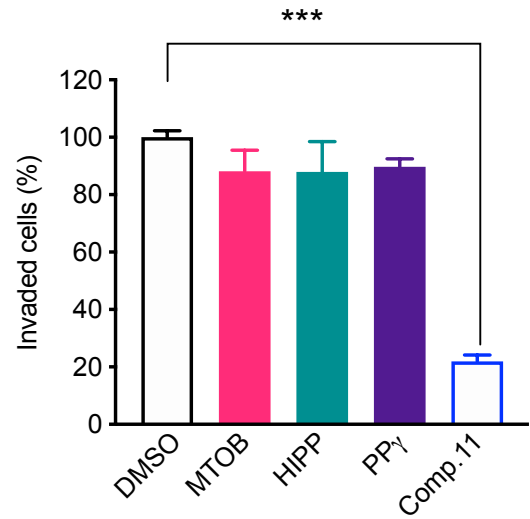

**C**

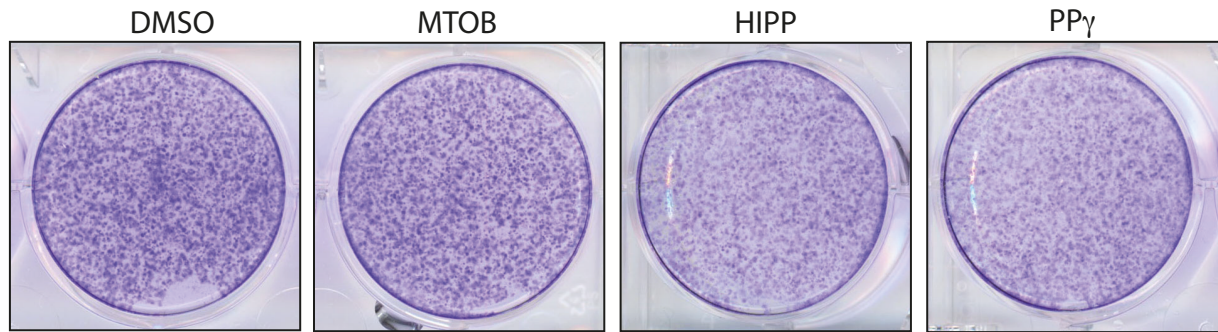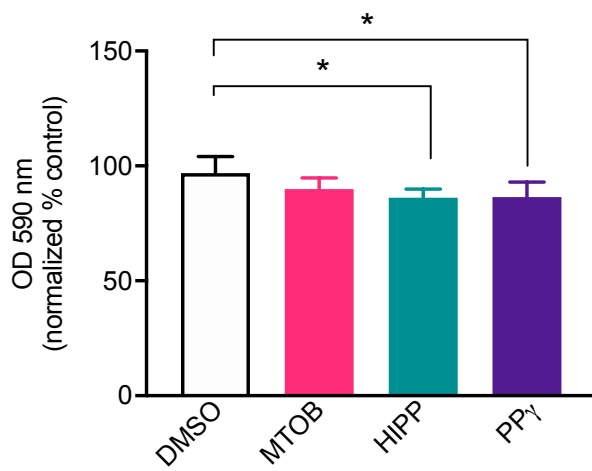

Supplement: Supplementary file 18 — Additional file 18: Supplementary Fig. 18. Analysis of CtBP1/BARS and CtBP2 depletion in A375MM and B16F10 melanoma cells subjected to wound closure assays and to Matrigel invasion assays reported in Fig. 8. Representative Western blotting with anti-CtBP1/BARS, anti-CtBP2 and anti-GAPDH antibodies (as indicated) of A375MM cells and B16F10 cells treated for 24 h with DMSO (vehicle control) or Comp.11 (15 μM) or transfected for 48 h with non-targeting or with CtBP1/BARS siRNAs or CtBP2 siRNAs. Molecular weight standards (kDa) are indicated on the left of each panel. [file 13046_2024_3044_MOESM18_ESM.pdf]

A

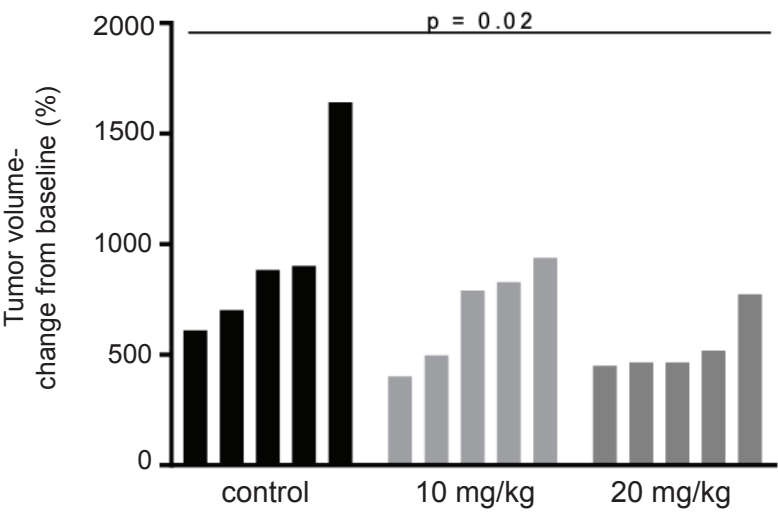

B

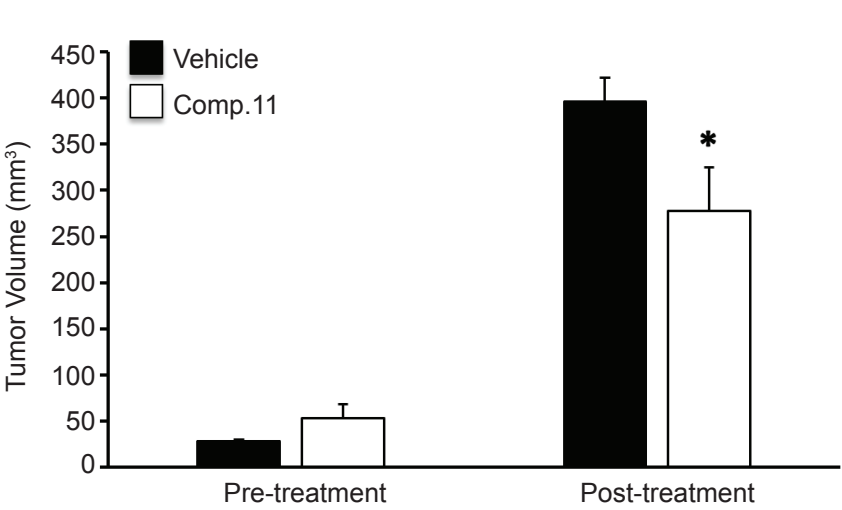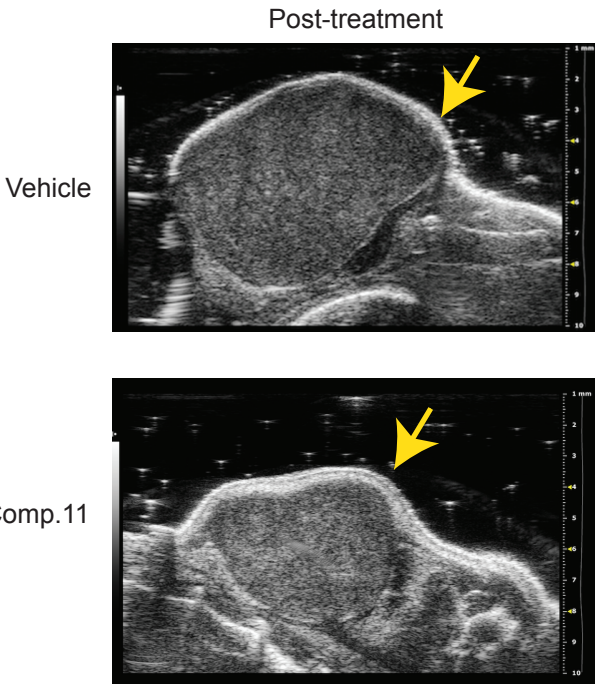

Supplement: Supplementary file 19 — Additional File 19: Supplementary Fig. 19. A. Waterfall plot of the fold-change in tumor volume compared with baseline [determined by caliper and calculated as (length $\times$×width2)/2] of A375MM xenograft tumors after 2 week of Comp.11 treatment. B. Representative High Frequency Ultrasound (HFUS) images of tumors (yellow arrow) at 2 weeks of Comp.11 (20 mg/kg body weight) or vehicle treatment (3 mice for each treatment group). Tumor volumes are expressed as mean ± SEM, *P=0.04, Test Kruskal-Wallis followed by post hoc Tukey. [file 13046_2024_3044_MOESM19_ESM.pdf]
