# Supplementary material for: Identification and characterization of a new potent inhibitor targeting CtBP1/BARS in melanoma cells
Source: J Exp Clin Cancer Res. 2024 May 6;43:137. doi: 10.1186/s13046-024-03044-5 (PMC11071220; doi:10.1186/s13046-024-03044-5)
Supplement: Supplementary file 20 — Additional File 20: Supplementary Table 1. List of oligonucleotides used in this study (human genes). [file 13046_2024_3044_MOESM20_ESM.docx]

**Supplementary Table 1**. List of oligonucleotides used in this study (human genes).

| **Cellular function** | **Human Primer name** | **Sequence 5’-3’** |
| --- | --- | --- |
| Cell Cycle progression | P16^INK4a^_fw | CACCAGAGGCAGTAACCATG |
|  | P16I^NK4a^_rev | TGATCTAAGTTTCCCGAGGTTTC |
|  | P21_fw | CTTGTACCCTTGTGCCTCG |
|  | P21_rev | GGCGTTTGGAGTGGTAGAA |
|  | P14^ARF^_fw | TGATGCTACTGAGGAGCCAGC |
|  | P14^ARF^_rev | AGGGCCTTTCCTACCTGGTC |
|  | CCND1_fw | CCTCACACGCTTCCTCTC |
|  | CCND1_rev | GCTTCGATCTGCTCCTGG |
| Cell survival | P53_fw | AGAGTCTATAGGCCCACCCC |
|  | P53_rev | GCTCGACGCTAGGATCTGAC |
|  | PTEN_fw | GCACAAGAGGCCCTAGATTTC |
|  | PTEN_rev | GCCTCTGACTGGGAATAGTTAC |
| Apoptosis | BRCA1_fw | GGACAGAGGACAATGGCTTC |
|  | BRCA1_rev | TCCTGGCACTGGTAGAGT |
|  | BRIP1_fw | GCTTAGCCTTACTTTGTTCTGC |
|  | BRIP1_rev | TTTCACTTACGCCCTCATCTG |
| EMT  EMT  EMT | JAMA1_fw | ACCGCCTATCATCTGCATTTGCCTTACTCA |
|  | JAMA1_rev | ACCCCCGCCATTTTTGCTGTCTACTTA |
|  | E-cadherin_fw  E-cadherin_rev | CCGAGAGCTACACGTTCAC  AATAGGCTGTCCTTTGTCGAC |
|  | β-catenin_fw | AGCTTCCAGACACGCTATCAT |
|  | β-catenin_rev | CGGTACAACGAGCTGTTTCTAC |
|  | ZO1_fw | GGACTCTCGCTGGTCTACCT |
|  | ZO1_rev | GGGCACAATATGCAGGCAGA |
|  | occludin_fw | TCAGGGAATATCCACCTATCACTTCAG |
|  | occludin_rev | CATCAGCAGCAGCCATGTACTCTTCAC |
|  | DSG2_fw | TGTTACCAGCATTCTTGATCGAG |
|  | DSG2_rev | GCGTAGCTCTAAGGGTTTCTC |
|  | plakoglobin_fw | CTCTGTGCGTCTCAACTATGG |
|  | plakoglobin_rev | AGATTCCTGATCAAGCCGATG |
|  | N-cadherin_fw | CAGACCTGAGTTCTTACACCAG |
|  | N-cadherin_rev | TGGGATCGTCAGCATCAATTG |
|  | Vimentin_fw | CCAAGACCTGCTCAATGTTAAG |
|  | Vimentin_rev | GGAAAAGTTTGGAAGAGGCAG |
|  | VCAN_fw | TGGATTTGAGCAGTGTGACG |
|  | VCAN_rev | CCTGCCTTTCCCATCTTATCTC |
|  | GAPDH_fw | CCACATCGCTCAGACACCAT |
|  | GAPDH_rev | AGTTAAAAGCAGCCCTGGTGAC |
|  | CtBP1/BARS_fw | CTGGAGAAGTTCAAAGCC |
|  | CtBP1/BARS_rev | CCATCCGACAAGTAAGGG |
|  | CtBP2_fw | AAGAGACAGCGATTGGACAG |
|  | CtBP2_rev | CATCTCCACAGTGCAGTCG |
