# Supplementary material for: Identification and characterization of a new potent inhibitor targeting CtBP1/BARS in melanoma cells
Source: J Exp Clin Cancer Res. 2024 May 6;43:137. doi: 10.1186/s13046-024-03044-5 (PMC11071220; doi:10.1186/s13046-024-03044-5)
Supplement: Supplementary file 21 — Additional File 21: Supplementary Table 2. List of oligonucleotides used in this study (murine genes). [file 13046_2024_3044_MOESM21_ESM.docx]

**Supplementary Table 2**. List of oligonucleotides used in this study (murine genes).

| **Application** | **Mouse Primer name** | **Sequence 5’-3’** |
| --- | --- | --- |
| Cell Cycle progression | mP21_fw | CGAGAACGGTGGAACTTTGAC |
|  | mP21_rev | CAGGGCTCAGGTAGACCTTG |
|  | mCCND1_fw | TAGGCCCTCAGCCTCACTC |
|  | mCCND1_rev | CCACCCCTGGGATAAAGCAC |
| Cell survival | mP53_fw | GCTTCCACCTGGGCTTCCTG |
|  | mP53_rev | CCACAACTGCACAGGGCAC |
|  | mPTEN_fw | AAGACCATAACCCACCACAGCT |
|  | mPTEN_rev | ACACCAGTCCGTCCCTTTCCAG |
| Apoptosis | mBRCA1_fw | GCTTGACACGGGAATGCAGCTT |
|  | mBRCA1_rev | CTGGATGATCGACGCCTCCTCA |
|  | mBRIP1_fw | AAGCTTTGCAAGTGCCCTGGAG |
|  | mBRIP1_rev | GCCTACTCTGGAAGTGGCCTGT |
| EMT | mJAM1_fw | GGCAGCACAACTGCACTTGTGT |
|  | mJAM1_rev | CTGACCTCCCCGTAGTTCTGGC |
|  | mE-cadherin_fw | CCTGGAGAGAGGCCATGTCCTG |
|  | mE-cadherin rev | ATTCAAAGTGGCGACAGACGGC |
|  | mβ-catenin_fw | TGCCACACGTGCAATTCCTGAG |
|  | mβ-catenin_rev | GCGCATGATGGCATGTCTGGAA |
|  | mZO1_fw | GGAACAGCACACAGTGACGCTT |
|  | mZO1_rev | TCCCACTCTTCCTTAGCTGCTGA |
|  | moccludin_fw | GCAGCCTCGGTACAGCAGCA |
|  | moccludin_rev | AGGACTCCCCACCTGTCGTG |
|  | mDSG2_fw | TGTTACCAGCATTCTTGATCGAG |
|  | mDSG2_rev | GCGTAGCTCTAAGGGTTTCTC |
|  | mplakoglobin_fw | CTCTGTGCGTCTCAACTATGG |
|  | mplakoglobin_rev | AGATTCCTGATCAAGCCGATG |
|  | mN-cadherin_fw | GAGCCAACCCTGACTGAGGAGC |
|  | mN-cadherin_rev | TGGCGGGATGACCCAGTCTCTC |
|  | mVimentin_fw | ATGCTCCAGAGAGAGGAAGCCG |
|  | mVimentin_rev | CCGTTCAAGGTCAAGACGTGCC |
|  | mVCAN_fw | CGGACCTATGGATTCCGCTCTC |
|  | mVCAN_rev | CTTGTACACTCTGCTTCGGCCT |
|  | mGAPDH_fw | CCTCGTCCCGTAGACAAAATG |
|  | mGAPDH_rev | CAATCTCCACTTTGCCACTG |
|  | mCtBP1/BARS_fw | CTGGGGATCTAGGCATCGCAGT |
|  | mCtBP1/BARS_rev | TAGCCAGGTGGTTCGTCGGTAC |
|  | mCtBP2_fw | GTATCGGCGGAACACATGGCTC |
|  | mCtBP2_rev | TGTGCGACCAAAGCCAATGAGG |
